# Risk Factors for Mortality from Acute Lower Respiratory Infections (ALRI) in Children under Five Years of Age in Low and Middle-Income Countries: A Systematic Review and Meta-Analysis of Observational Studies

**DOI:** 10.1371/journal.pone.0116380

**Published:** 2015-01-30

**Authors:** Michela Sonego, Maria Chiara Pellegrin, Genevieve Becker, Marzia Lazzerini

**Affiliations:** 1 WHO Collaborating Centre for Maternal and Child Health, Institute for Maternal and Child Health IRCCS Burlo Garofolo, Trieste, Italy; 2 Department of Medicine, Surgery, and Health Science, University of Trieste, Trieste, Italy; INDEPTH Network, GHANA

## Abstract

**Objective:**

To evaluate risk factors for death from acute lower respiratory infections (ALRI) in children in low- and middle-income countries.

**Design:**

Systematic review and meta-analysis.

**Study selection:**

Observational studies reporting on risk factors for death from ALRI in children below five years in low- and middle income countries.

**Data sources:**

Medline, Embase, Global Health Library, Lilacs, and Web of Science to January 2014

**Risk of bias assessment:**

Quality In Prognosis Studies tool with minor adaptations to assess the risk of bias; funnel plots and Egger's test to evaluate publication bias.

**Results:**

Out of 10655 papers retrieved, 77 studies from 39 countries (198359 children) met the inclusion criteria. Host and disease characteristics more strongly associated with ALRI mortality were: diagnosis of very severe pneumonia as per WHO definition (odds ratio 9.42, 95% confidence interval 6.37‒13.92); age below two months (5.22, 1.70‒16.03); diagnosis of *Pneumocystis Carinii* (4.79, 2.67 ‒ 8.61), chronic underlying diseases (4.76, 3.27‒ 6.93); HIV/AIDS (4.68, 3.72‒5.90); and severe malnutrition (OR 4.27, 3.47‒5.25). Socio-economic and environmental factors significantly associated with increased odds of death from ALRI were: young maternal age (1.84, 1.03‒3.31); low maternal education (1.43, 1.13‒1.82); low socio-economic status (1.62, 1.32‒2.00); second-hand smoke exposure (1.52, 1.20 to 1.93); indoor air pollution (3.02, 2.11‒4.31). Immunisation (0.46, 0.36‒0.58) and good antenatal practices (0.50, 0.31‒0.81) were associated with decreased odds of death.

**Conclusions:**

Host and disease characteristics as well as socio-economic and environmental determinants affect the risk of death from ALRI in children. Together with the prevention and treatment of chronic diseases, interventions to modify underlying risk factors such as poverty, lack of female education, and poor environmental conditions, should be considered among the strategies to reduce ALRI mortality in children in low- and middle-income countries.

## Introduction

Acute lower respiratory infections (ALRI), such as pneumonia and bronchiolitis, are the leading cause of morbidity and mortality in children under five years of age. According to recent estimates, every year about 120–156 million cases of ALRI occur globally with approximately 1.4 million resulting in death. More than 95% of these deaths occur in low and middle income countries (LMIC) [1–3].

ALRI are caused by a number of infective agents, with *Streptococcus pneumoniae* being generally the most frequently identified bacterial agent, and *Respiratory Syncytial Virus* being the most frequent viral agent [4]. A large number of factors determine whether the contact with an etiologic agent will result in a severe episode of ALRI, and whether the episode will result in death. Some of these factors are related to the child (e.g. age, sex, underlying diseases), others to the disease (e.g. type of infection), others may be related to the environment, the family and its socio-economic status, or to the health system and type of care [5], [6].

A recent systematic review has evaluated the risk for severe outcomes and death from influenza according to age and comorbidity [7]. Previous systematic reviews have reported on the association between pneumonia mortality in children and single risk factors, such as hypoxemia [8], inadequate breastfeeding [9–11], malnutrition [12], [13], indoor air pollution from solid fuels [14], [15]. To our knowledge, no recent systematic review has synthesised the evidence on the wide range of factors associated with an increased risk of death from ALRI among children. The aim of this work is to systematically review and meta-analyse the evidence on the association between potential risk factors and ALRI mortality in children in LMIC. A better understanding of these risk factors will help develop strategies to reduce the global burden of mortality from ALRI in children.

## Methods

### Search strategy and eligibility criteria

In conducting this review we followed the guidelines reported in the PRISMA (Preferred Reporting Items for systematic reviews and meta-analyses) [16] and the MOOSE (Meta-analysis of Observational Studies) [17]. A protocol including detailed methods of the review was developed before starting the review (S1 Protocol).

We searched to January 2014 the following databases: MEDLINE through Pubmed (from 1956); Embase through OVID (from 1974); Global Health Library (WHO web site, no date restrictions), LILACS through the Virtual Health Library (no date restrictions); Science Citation Index Expanded (SCI-EXPANDED) through Web of Science (from 1992); Social Sciences Citation Index (SSCI) through Web of Science (from 1992). The search strategy is reported in S1 Protocol. Manual searches of reference lists were also performed. We did not apply any language restrictions.

Observational studies were eligible for inclusion if they satisfied the following three criteria: 1) the study reported on children under 5 years of age in LMIC, as defined by the World Bank [18]; 2) the outcome of interest was death from ALRI, as defined by the study authors; 3) the study reported the association between death from ALRI and at least one possible risk factor.

Both studies at hospital level and in the community were included. The following studies were excluded: studies reporting only on long-term post-discharge follow up (i.e. more than one year); studies reporting selectively on children with very specific co-morbidities, such as studies on children with cancer, organ transplant, burns, ventilator-acquired pneumonia, or very low birth weight; studies reporting selectively on specific diseases, such as avian influenza, SARS or H1N1; studies on single micronutrients; and studies reporting less than five events (i.e. five deaths). Signs and symptoms of the disease were not included in this review, as they have been considered elsewhere [19], [20].

### Data collection

Two authors (ML and MS) independently selected potentially eligible studies for inclusion. Disagreements were solved by discussion. The full text of all eligible citations was examined in detail, except for two articles in Chinese, which were not available in full text. Twelve authors were contacted for additional information and eight provided the full text, additional data, and/or clarifications on the published data.

Two authors (MS and MCP) extracted data from included studies, using a pre-piloted data-extraction form. Disagreements were solved by discussion between the two authors and consensus with a third author (ML).

We extracted information regarding: the definition for ALRI; study design; sample size, characteristics of the population; setting; risk factors evaluated; type of analysis performed (univariate or multivariate); confounders. Risk factors were classified as for Wonodi at al. [6], with minor modifications.

We extracted both unadjusted and adjusted OR, risk ratios (RR), case fatality ratios, hazard ratios (HR), and crude numbers. When possible, we converted RR/HR to OR, using a formula for computing OR from RR [21]. To avoid mistakes due to data manipulation, data were first extracted as in the original paper, and then converted as needed.

When studies used different definitions or different cut-off points for the same risk factor we noted these differences in the forest plots.

In studies including also children over five years of age, only data on children under five were extracted. If sorting was not possible, we included the study only if at least 80% of the children were under five years.

### Assessment of risk of bias in included studies

Two review authors (MS and MCP) independently assessed the risk of bias using the Quality In Prognosis Studies (QUIPS) tool with minor adaptations (see S1 Protocol) [22].

### Statistical analyses

When meta-analysis was possible and appropriate, for each risk factor we generated a pooled OR using the inverse-variance weighting method. As we expected high heterogeneity in the population, the definition of ALRI, the definition of risk factors, and the methodology, we selected a priori the DerSimonian and Laird random effect model [23], which accounts for intra- and inter-study variability. Pooled data were presented in forest plots; data that could not be meta-analyzed were presented in tables and text.

We tested the null hypothesis that all studies evaluate the same true effect by the Cochran’s Q test, with p<0.05 considered statistically significant. The degree of heterogeneity between studies was assessed by visual inspection of the forest plots and I-squared (I^2^) statistic with its 95% confidence intervals. Heterogeneity was considered low for I^2^ values between 25%-50%, moderate for 50%-75%, and high for ≥75% [24].

We performed sensitivity analyses to examine the effect of removing the studies with high risk of bias and substituting the adjusted OR for the crude OR, when the former was available.

Using subgroup and metaregression analysis, we explored the effect of the following study-level factors: i) different definitions of ALRI used (WHO definition [25], [26] versus other definitions); ii) HIV country prevalence (high HIV prevalence versus non-high HIV prevalence), where a high HIV prevalence was defined by us as greater than 5% in the population of 15–49 years-olds at the time of the study, as reported by the World Bank [27]; iii) risk of female mortality (countries with “female under-five mortality higher-than-expected” versus countries without), where ”higher-than-expected female under-five mortality” is defined by Alkema et al. [28] as the ratio of estimated to expected female under-five mortality from all causes higher than one. The effect of the country risk of female mortality was evalutated only on sex as a risk factor.

Subgroup analyses were performed when at least two studies for each subgroup were available.

We assessed potential publication bias and small study effects with funnel plots and Egger test [29]. Statistical analysis was performed using Stata version 12 [30].

## Results

**Figure 1:**
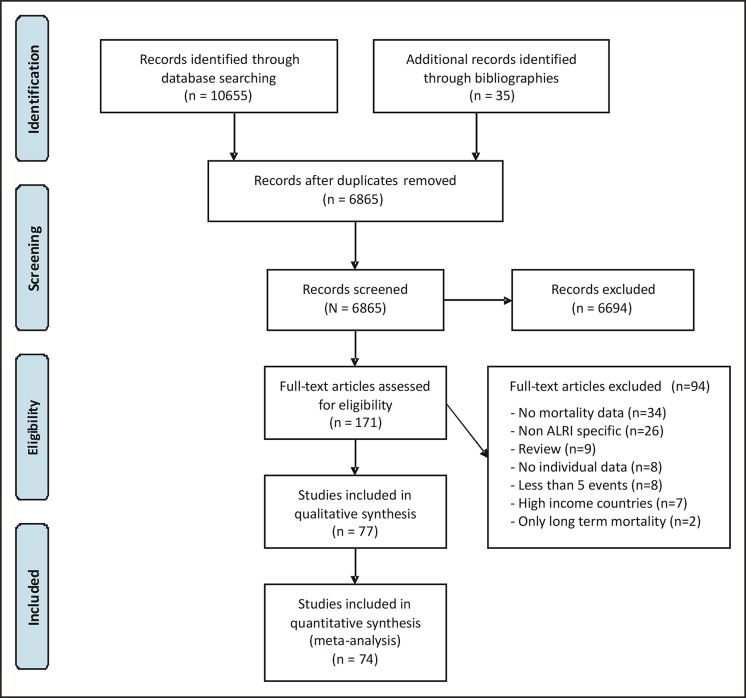
Flow chart for bibliographic search.

The systematic search yielded 10655 records, plus 35 records acquired from bibliographies of retrieved studies, of which 6865 records remained after removal of duplicates (Fig. 1). Of these, 77 studies met our inclusion criteria, for a total of 198359 children (see S1 References for the reference list of the included studies).

**Figure 2:**
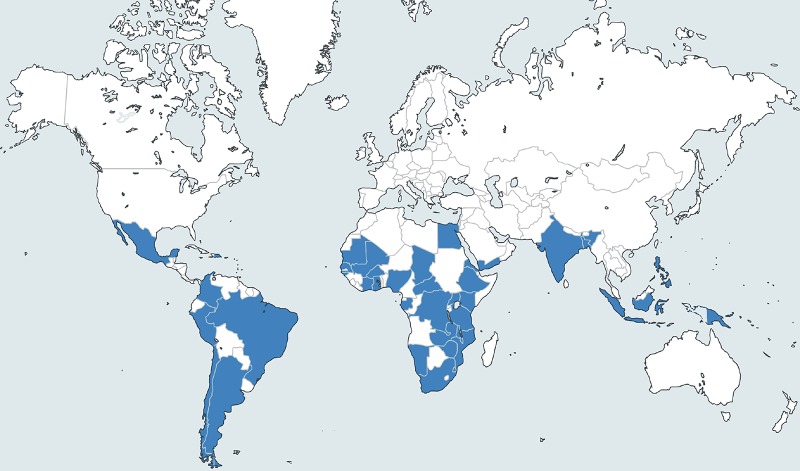
Location of the 77 included studies.

The studies reported on children in 39 LMIC. There were two multi-country studies: one took place in India, Peru and Ghana; the other included 16 countries in the sub-Saharan Africa. Of the remaining 75 studies, 34 (45.3%) were held in the African Region, 16 (21.3%) in the region of the Americas, 13 (17.3%) in South-East Asia, nine (12% in the Western Pacific) and three (4%) in the Eastern Mediterranean (Fig. 2). There were 58 (75.3%) hospital-based studies and 19 (24.7%) community-based studies. The study settings was urban in 48 (62.3%) studies, rural in 17 (22.1%), mixed in 10 (13%), and unknown in two studies. In 32 (41.6%) studies, the WHO definition for pneumonia, with minor modifications, was used (see S1 Table for the detailed characteristics of each study).

Table 1 reports the synthesis of the main results for 30 risk factors that could be meta-analysed and 20 risk factors reported by only one study each (forest plots for each factor and further details for each study are reported in S1 Forest Plots). Among 30 risk factors included in the meta-analysis, 22 (73%) were associated with significant higher odds of mortality from ALRI.

**Table 1:** Synthesis of the main results from this systematic review.

**Category of risk factor**	**Risk factors (nº of studies reporting on the risk factor)**	**Comparisons**	**Nº of studies included in the meta-analysis**	**Nº of children included in the meta-analysis**	**Pooled OR**	**I^2^ (%)**	**Non meta-analysed studies**
Child factors	Age (42)	Age <1y vs >1y	28	63629	2.35 (1.72–3.21)	84.4	6
		Age <6m vs >12m	9	7390	2.22 (1.59–3.10)	55.5	0
		Age <6m vs 7–59m	4	2337	1.97 (0.98–3.98)	78.3	1
		Age <2m vs >12m	7	12279	5.22 (1.70–16.03)	87.3	2
	Female sex (23)	Female vs male	23	20385	1.15 (1.03–1.28)	11.7	0
	Prematurity (7)	Yes vs no	6	2983	2.43 (1.65–3.57)	0.0	1
	Birth order (6)	Higher vs lower	6	1783	1.39 (0.92–2.11)	65.3	0
	Low birth weight (12)	<2500g vs >2500g	9	7973	2.78 (2.03–3.82)	39.2	3
	Malnutrition (39)	Severe malnutrition vs non-malnourished	21	13478	4.27 (3.47–5.25)	42.9	6
		Moderate malnutrition vs non-malnourished	18	13608	2.46 (1.89–3.19)	64.4	
	Breastfeeding (15)	Inadequate breastfeeding vs adequate	12	3941	1.79 (1.18–2.70)	75.9	3
	HIV/AIDS (14)	Yes vs no	14	4171	4.68 (3.72–5.90)	0.0	0
	Chronic diseases (12)*	Yes vs no	12	7473	4.76 (3.27–6.93)	44.9	0
	Malaria (4)	Yes vs no	3	3991	1.46 (1.02–2.11)	0.0	1
	Measles (6)	Yes vs no	6	2420	3.78 (1.81–7.87)	73.9	0
	Diarrhoea/dehydration (6)	Yes vs no	6	5570	2.82 (1.80–4.43)	65.3	0
	Previous ALRI (5)	Yes vs no	5	3517	2.78 (1.55–4.98)	57.8	0
	Severity of pneumonia as per WHO definition (14)	Severe pneumonia vs pneumonia	6	7581	3.24 (2.27–4.61)	0.0	0
		Very severe peumonia vs severe pneumonia or pneumonia	12	7232	9.42 (6.37–13.92)	48.7	0
	Respiratory Syncytial Virus (6)	Yes vs no	6	7595	0.46 (0.29–0.74)	0.0	0
	Pneumocystis Carinii Jirovecii (5)	Yes vs no	5	952	4.79 (2.67–8.61)	33.4	0
Mother factors	Mother’s age (5)	Younger vs older	4	1225	1.84 (1.03–3.31)	66.9	1
	Mother’s educational level (15)	Lower vs higher	14	26130	1.43 (1.13–1.82)	42.3	1
	Mother’s paid job (3)	Yes vs no	3	520	1.23 (0.55–2.73)	71.7	0
	Other significant maternal factors (2)	Diseases in pregnancy: 1 study, 231 children, OR 2.62 (1.41–4.85); Maternal tuberculosis: 1 study, 358 children, OR 4.36 (1.42–13.24);					
	Other non significant maternal factors (4)	Maternal parity ≤2: 1 study, 1314 children, OR = 0.70 (0.49–1.0); Black race of the mother vs white: 1 study, 303 children, OR 1.41(0.73–2.70); Cesarean delivery vs vaginal: 1 study, 381 children, OR 0.72 (0.42–1.25); Maternal height ≥150 cm vs < 145 cm: 1 study, 1677 children, RR 0.72 (0.46–1.12); Unwanted pregnancy: 1 study, 231 children, OR 0.60 (0.35–1.0)					
Socio-economic and environmental factors	Socio-economic status (9)	Low income or low social class of the father vs higher	9	13908	1.62 (1.32–2.00)	0.0	0
	Other non significant socioeconomic factors (1)	Sleeping in a wooden bed with no covering: 1 study, 184 children, OR 1.7 (0.5–6.36); Humidity in the house, 1 study, 258 children, OR: 2.75 (0.92–8.16)					
	Water, sanitation and hygiene (4)	Lack of sewerage/latrine vs presence	3	904	1.82 (1.30–2.54)	0.0	0
		Lower quality of drinking water	3	1922	2.85 (1.28–6.36)	80.2	
	Other non significant hygiene factors (1)	Dirty latrine: 1 study, 304 children, OR 1.83 (0.91–3.60); No agent used for washing hands: 1 study, 304 children,OR 1.27 (0.58–2.80); Water used for washing hands (other vs tube well): 1 study, 304 children OR 1.41 (0.76–2.63)					
	Crowding (9)	More people vs less	9	3285	1.33 (0.86–2.05)	77.3	0
	Second-hand smoke exposure (8)	Yes vs no	8	3044	1.52 (1.20–1.93)	0.0	0
	Indoor pollution (6)	Yes vs no	6	32635	3.02 (2.11–4.31)	42.9	0
	Setting of residence (3)	Rural vs urban	3	78752	2.10 (0.95–4.66)	85.0	0
	Seasonality (4)	Wet vs dry season	4	18727	1.06 (0.65–1.72)	63.8	0
Health-care-related factors	Routine immunisation (12)	Yes vs no	12	6006	0.46 (0.36–0.58)	11.1	0
	Good ante-natal practices (3)	Antenatal care and birth-spacing vs no	3	646	0.50 (0.31–0.81)	23.3	0
	Other significant preventive factors (2)	Maternal child card present, 1 study, 1314 children, OR 0.02 (0.01–0.04); Child ever visited welfare clinic: 1 study, 248 children, OR 0.13 (0.05–0.29)					
	Pre-hospital care (3)	Pre-hospital antibiotics or community health worker consultation vs no	3	4824	1.75 (0.72–4.25)	79.0	0
	Other significant health-care factors (1)	Lack of identification of ALRI by caretakers: 1 study, 236 children, OR 2.13 (1.15–3.94); Non-institutional barriers to access to care: 1 study, 236 children, OR 3.12 (1.41–6.93); Late referral by caretakers: 1 study, 236 children, OR 20.0 (2.70–148.60); Late referral from primary care to hospital: 1 study, 236 children, OR 7.56 (3.78–15.13)					
	Others non-significant health-care factors (2)	Poor understanding of ALRI by caretakers, 1 study, 195 children, OR 0.60 (0.24–1.49); Dispensary ≤3km: 1 study, 1314 children, OR 0.65 (0.60–1.19)					

Congenital heart disease, other congenital diseases, rickets, bronchopulmonary dysplasia, developmental delay.

Abbreviations: OR = Odds Ratio; I^2^= I squared statistic; y = years; m = months; g = grams

### Child-related factors

**Figure 3:**
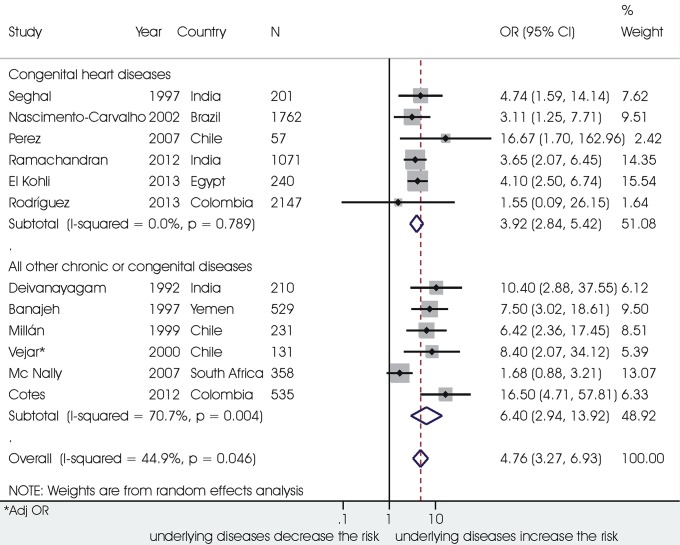
Association between comorbidity with chronic diseases and death from ALRI.

**Figure 4:**
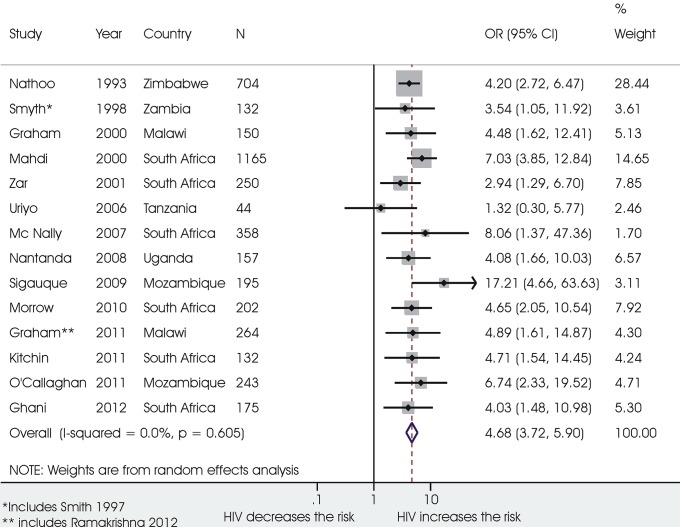
Association between HIV/AIDS and death from ALRI.

Child-related factors showing the stronger association with mortality were: diagnosis of very severe pneumonia as defined by WHO (odds ratio 9.42, 95% confidence interval 6.37 ‒ 13.92; 7232 children, I^2^ 48.7%); age <2 months (5.22, 1.70 **‒**16.03; 12279 children, I^2^ 87.3%); diagnosis of *Pneumocystis Carinii* (4.79, 2.67 ‒ 8.61, 952 children, I^2^ 33.4%); co-morbidity with chronic diseases (4.76, 3.27 ‒ 6.93; 7473 children, I^2^ 44.9%) (Fig. 3); HIV/AIDS (4.68, 3.72 ‒ 5.90; 4171 children, I^2^ 0%) (Fig. 4), and severe malnutrition (4.27, 3.47 ‒ 5.25; 13478 children, I^2^ 42.9%). An increased risk of death was also associated with: prematurity (2.43, 1.65 ‒ 3.57; 2983 children; I^2^ 0%); low birth weight (2.78, 2.03 ‒ 3.82; 7973 children; I^2^ 39.2%) inadequate breastfeeding practices (1.79, 1.18 ‒ 2.70; 3491 children; I^2^ 75.9%); co-morbidity with malaria (1.46, 1.02 ‒ 2.11; 3991 children; I^2^ 0%); co-morbidity with diarrhoea (2.82, 1.80 ‒ 4.43; 5570 children; I^2^ 65.3%), co-morbidity with measles (3.78, 1.81 ‒ 7.87, 2420 children, I^2^ 73.9%), and a previous episode of ALRI (2.78, 1.55 ‒ 4.98; 3517 children; I^2^ 57.8%). Female sex was associated with a 15% increase in the odds for mortality in 23 studies on 20385 children, with low heterogeneity between studies (I^2^ 11.7%). A diagnosis of *Respiratory Syncytial Virus* was significantly associated with a decreased odds of mortality (0.46 (0.29 ‒ 0.74; 7595 children), with high heterogeneity among studies (I^2^ 73.9%).

### Mother-related factors

**Figure 5:**
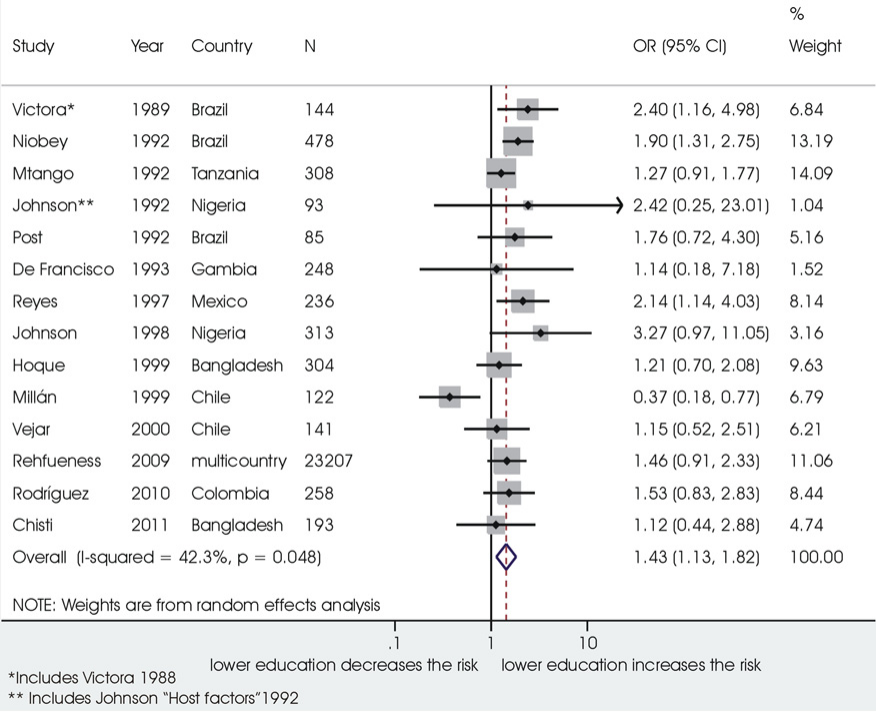
Association between mother’s low educational level and death from ALRI.

In 14 studies on 26130 children, low maternal education level was associated with significantly increased odds in ALRI mortality (1.43, 1.13 ‒ 1.82, I^2^ 42.3%) (Fig. 5). Mother’s young age was associated in four studies to an increased risk of death (1.84, 1.03 ‒ 3.31; 1225 children, I^2^ 66.9%); three small studies reported on the effect of the mother having a paid job, without significant association with ALRI deaths. Other maternal factors were investigated in single studies only.

### Socioeconomic and environmental factors

**Figure 6:**
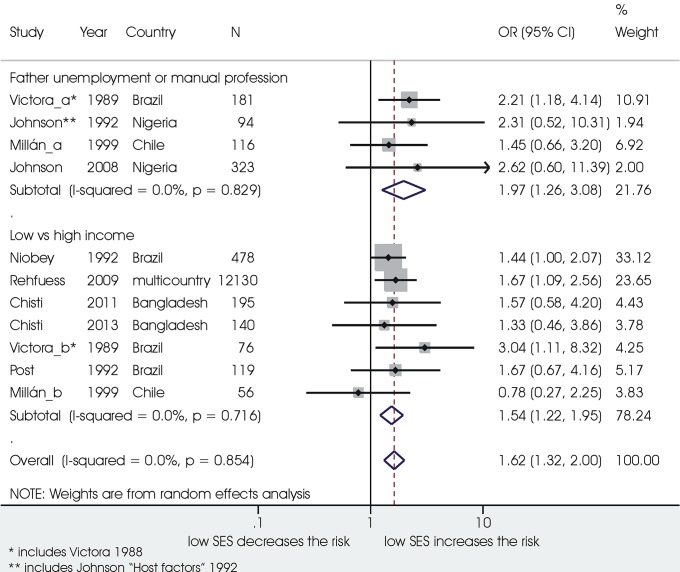
Association between low socioeconomic status and death from ARLI.

Low socioeconomic status was associated in nine studies on 13908 children with a significant 62% increased odds of mortality compared with higher socioeconomic status and no heterogeneity between the studies (Fig. 6). A significant association with death from ALRI was also found for other factors related to the socioeconomic status, such as lack of sewerage/latrines (1.82, 1.30 ‒ 2.54; 904 children, I^2^ 0%) and low quality of drinking water (2.85, 1.28 ‒ 6.36; 1922 children, I^2^ 80.2%).

Indoor pollution from solid fuels was associated with a significantly increased risk of ALRI mortality in six studies on 32635 children (3.02, 2.11 ‒ 4.31, I^2^ 42.9%). Second-hand smoke was associated with an increased mortality in eight studies on 3044 children (1.52 1.20 ‒ 1.93; I^2^ 0%).

### Health-care-related factors

Few studies reported on the association between health care related factors and risk of death from ALRI in children. Preventive practices, such as routine vaccinations (0.46, 0.36 ‒ 0.58; 6006 children, I^2^ 11.2%) and good pre-natal care (0.50, 0.31 ‒ 0.81; 646 children, I^2^ 23.3%), were significantly associated with decreased odds of death. Antibiotics or consultation before hospitalisation showed inconsistent findings in three studies (1.75, 0.72 ‒ 4.25; children I^2^ 79%). Other health care related factors were explored only by one single study each. (Table 1)

### Risk of bias, subgroup analyses and sensitivity analyses

S2 Table synthesises the risks of bias for the studies included in the review: overall, 36 (46.7%) studies were considered at high risk of bias, with lack of adjustment for confounding being the most frequent risk of bias.

Only a limited number of risk factors had a sufficient number of studies to perform subgroup and meta-regression analysis. Subgroup analysis did not substantially changed results (S3 Table). Some comparisons with a low number of contributing studies lost statistical signicance. The strength of the association between diarrhoea and ALRI mortality was significantly higher in studies using a non-WHO definition of ALRI and in studies performed in low HIV setting (S3 Table).

Female sex was associated with higher odds for ALRI mortality in the subgroup of studies realized in countries with higher-than-expected female mortality (from all causes): OR 1.22, (1.02–1.45; I2 6.1%), while no association between sex and ALRI mortality was found for the subgroup of studies performed in countries without higher-than-expected female mortality (see third figure in S1 Forest Plots).

Results of sensitivity analyses are reported in S4 Table. Overall, for factors significantly associated with mortality, the direction of results did not change in the sensitivity analyses. After removing the studies with high risk of bias, two risk factors lost statistical significance: second-hand smoke exposure (1.34, 0.85 ‒ 2.13) and female sex (1.13, 0.96 ‒ 1.34 for all countries and 1.17, 0.92 ‒1.49 for countries with higher-than-expected female child mortality).

Substituting the adjusted OR for the crude OR led to a non-significant pooled OR for malaria (1.26, 0.86 ‒ 1.85) and to a marginally non-significant pooled OR for young mother age (1.78, 0.99 ‒ 3.20).

### Publication bias

Egger tests (S4 Table) and funnel plots (S1 Funnel Plots) did not suggest a publication bias for most risk factors examined with the exception of: co-morbidity with a chronic disease; measles; or severe pneumonia. Egger’s test suggested a risk of bias also for severe malnutrition, although the test became not significant after the exclusion of one single small study identified as an outlier.

## Discussion

This systematic review synthesises the available evidence on risk factors for death from ALRI in children in LMIC. Overall, many factors were associated with an increased odds for ALRI mortality, including host factors, maternal, socioeconomic, environmental, and health-care factors.

### Strengths and limitations

The strengths of this review include the comprehensive search strategy performed—with no language limitation—and the extensive amount of data reviewed.

A major limitation of the review is the quality of existing evidence. Nearly half of the studies were classified as “at high risk of bias”, with the lack of accounting for confounders being the most frequent risk of bias. However, the sensitivity analyses did not show major changes in the review findings after excluding studies with high risk of bias, suggesting that most results were not affected by the study quality.

Another limitation of this review is the completeness of the existing evidence. We made an effort to include all existing studies independently from the language of publication. Overall, data from 39 LMIC are captured in this review; however, not all risk factors have been explored in each country. Publication bias was suggested for a few risk factors. Generalisability can be considered acceptable only for factors analysed by an adequate number of studies.

Heterogeneity in results was moderate to high for many risk factors considered in the meta-analysis (Table 1), and this limits the strength of the evidence for these risk factors. Heterogeneity in study findings may be explained by heterogeneity in the characteristics of the studies, such as design, setting, type of population included, definition of ALRI, definition of the single risk factor. Subgroup analyses explained part of the heterogeneity among studies, but other characteritstics of the studies may influence the association between risk factors and ALRI mortality.

We were aware of the possibility of introducing bias at every stage of the review process; we tried to minimise this in a number of ways, following strictly methods and standards suggested for systematic reviews [16], [17].

### Interpretation of the results with respect to the existing literature

Results from this review confirm those from previous systematic reviews in regards to the association of inadequate breastfeeding [10], [11], malnutrition [12], [13], and indoor air pollution [14], [15] with ALRI mortality.

Many other findings from this review represent a novelty and deserve to be discussed in further details.

Among child related factors, female sex was associated with a significant 15% increased odds of mortality from ALRI in 23 studies with little heterogeneity of findings; in countries with higher than expected female mortality, girls had an a 22% increased odds of mortality. Other systematic reviews have shown that ALRI in children are overall both more frequent and more severe in males compared to females [1], [31]; results from our review, with an increased mortality risk in girls rather than boys, may be explained by gender inequities in quality of health care provided. More high quality studies are needed to confirm this hypothesis. Literature on gender related quality of care is still relatively limited and findings may well be setting-specific [32–34].

Comorbidity with chronic conditions was one of the factors more strongly associated with increased odds of death from ALRI. This is consistent with a recent review reporting a higher mortality from viral infections in congenital hearth diseases and bronchopulmonary dysplasia [35]. These findings are particularly important when considering the increased survival of children with chronic conditions [36].

Low maternal educational level, low socio-economic status and other related factors (i.e. lack of sewerage and latrine and poor quality of water) were significantly associated with ALRI mortality in this review. The effect of socioeconomic determinants on overall child mortality, both at within-country and between-countries levels, have been documented in several other studies [37], [38]. For example, a systematic analysis estimated that about half the reduction in child mortality in 175 countries between 1970 and 2009 could be attributed to the improvement of the educational level in women [39]. Our review adds evidence on the association between these factors and specific ALRI mortality in children. These results are also consistent with the distribution of the global burden of ALRI, with hospitalizations and deaths concentrating in the poorest countries [1], [3]. Low socioeconomic status is likely to increase the mortality risk through several factors, such as, among others, poor nutritional status, poor housing conditions, and reduced access to health care and preventive programmes [40].

Another new finding from this review is that second-hand smoke, which is a known risk factor for acquiring pneumonia [41], was associated with a significantly increased risk of death. Although further high quality studies are needed, this finding is quite relevant to public health if we consider that 40% of children world-wide are exposed to second-hand smoke [42].

This review showed that general preventive practices, such as antenatal care and routine immunisation, are strongly associated with decreased odds of ALRI death. The efficacy and effectiveness of single vaccines, such as pneumococcal and Haemophilus Influenzae vaccines, have been evaluated elsewhere [43], [44], and were not included in this review.

### Implication for further research

There is some evidence that second-hand smoke and gender inequities play a role in the global burden of ALRI mortality, although existing studies are insufficient to confirm these findings. Further high quality studies are needed to confirm the role of second-hand smoke and to evaluate if, in some countries, female discrimination could explain higher risk of dying from ALRI in girls.

This systematic review highlighted the lack of research on the association between health care related factors, including delayed access to care, and ALRI mortality. More research is needed in this area to better understand how health care related factors influence ALRI mortality in different settings [45].

### Implication for policies

Evidence from this review supports the global policies for pneumonia control, such as the recommendations from the Global Action Plan for the Prevention and Control of Pneumonia (GAPP) [46] and from the Integrated Global Action Plan for Pneumonia and Diarrhoea (GAPPD) [47] initiatives, launched by WHO and UNICEF respectively in 2009 and 2013. The key strategies recommended by GAPP and GAPPD focus on improved nutrition and adequate breastfeeding, reduction of low birth weight, prevention and management of HIV infection, immunization, control of indoor air pollution and supply of water and sanitation; on the other hand, the guidelines for case management take into account the age of the child, the severity of the pneumonia, and the possible co-morbidities such as HIV [19].

Besides the above cited recommendations, this review provides evidence for considering the need for additional interventions among the strategies with a specific potential for reducing the global burden of ALRI mortality, such as interventions aiming at preventing and treating chronic conditions other than HIV and interventions to promote socio-economic development.

Promotion of education and socioeconomic development should be included in the list of interventions to reduce deaths from ALRI not only because these are fundamental human rights, but also because a multisectoral approach that looks beyond the purely medical aspect and involves multiple sectors and policies is now being increasingly recognised as an efficient way to address health inequalities at global level, and to increase the number of lives saved [48].

### Conclusions

This review suggests that interventions to improve maternal education and socioeconomic status should be included among the existing strategies to reduce mortality from ALRI in children in low and middle income countries. Prevention and treatment of chronic conditions is also likely to reduce the burden of ALRI mortality. Further studies should evaluate the role of gender inequities and second-hand smoke exposure on the burden of ALRI mortality.

## Supporting Information

S1 Protocol Protocol of the study. (PDF)

S1 Forest Plots Forest plots of the association between different risk factors and death from ALRI, and results from studies that could not be meta-analysed. (PDF)

S1 Funnel Plots Funnel plots of risk factors with significant Egger’s test. (PDF)

S1 Table Characteristics of included studies. (PDF)

S2 Table Appraisal of the risk of bias of the included studies. (PDF)

S3 Table Subgroup and metaregression analysis. (PDF)

S4 Table Sensitivity analyses and results of Egger’s test. (PDF)

S1 References References of the included studies. (PDF)

S1 PRISMA Checklist (PDF)
