# Supplementary material for: Risk Factors for Mortality from Acute Lower Respiratory Infections (ALRI) in Children under Five Years of Age in Low and Middle-Income Countries: A Systematic Review and Meta-Analysis of Observational Studies
Source: PLoS One. 2015 Jan 30;10(1):e0116380. doi: 10.1371/journal.pone.0116380 (PMC4312071; doi:10.1371/journal.pone.0116380)
Supplement: S1 Protocol — (PDF) [file pone.0116380.s001.pdf]

S1 Protocol

Risk factors for mortality from acute lower respiratory infections in children in low and middle income countries: protocol for a systematic review and meta-analysis of observational studies

Contents

	Page
Protocol	1
Table 1: Factors that may modify the risk of death in children with ALRI	6
Annex 1: Search strategy	7
Annex 2: Modified Quality In Prognosis Studies (QUIPS) tool	8
Annex 3: Data Extraction Sheet	11

BACKGROUND

Acute lower respiratory infections (ALRI), such as pneumonia and bronchiolitis, are the leading cause of morbidity and mortality in children under five years of age. According to the most recent estimates, every year about 120-156 millions cases of ALRI occur globally with approximately 1.4 million children dying annually due to pneumonia, and more than 95% of these deaths occurring in children in low and middle income countries (LMIC).[1-4]

A large numbers of potential risk factors has been associated with either an increased incidence or an increased mortality from ALRI in children.[5-7] These include: a) child-related risk factors (such as malnutrition, HIV, and many others); b) parental risk factors (e.g. tuberculosis, age of the mother, etc) c) disease-related risk factors (e.g. duration and severity of the diseases); d) environmental factors (e.g. indoor smoke pollution, etc); e) socio-economical factors; f) health-services factors, and others (**Table 1**).

To our knowledge no recent systematic review has synthesised the evidence on a wide range of factors that may increase the risk of death in children with ALRI. Systematic reviews have reported on the association between pneumonia mortality in children and single risk factors, respectively breastfeeding,[8] hypoxia,[9] malnutrition,[10,11] and indoor air pollution.[12] Most of these reviews were published several years ago. A review published nearly twenty years ago reported on overall determinants of negative outcomes in pneumonia in adults.[13]

The objective of this review is to synthesise the evidence on all potential risk factors for death from ALRI in children in LMIC.

A better understanding of the ALRI mortality risk factors in children will help shaping more effective interventions. The results of this systematic review may also help in developing predictive models to identify cases at higher risk of death and to treat them more promptly.

METHODS

This review will be conducted following the guidelines reported in the PRISMA (Preferred reporting items for systematic reviews and meta-analyses) and the MOOSE (Meta-analysis of Observational Studies)[14,15]

Eligibility

Studies will be eligible for inclusion if they will satisfy all the following four criteria.

1) Population: the study reports on children under 5 years of age with ALRI in LMIC (as for the World Bank definition of LMIC at the time of the study).[16] Both studies in the community and in hospital will be included.

2) Risk factors: the study reports the association between deaths in children with ALRI and any possible risk factors, including a) child-related risk factors, b) maternal and paternal risk factors, c) disease related risk factors, d) environmental, e) socio-economical, f) health-services factors, g) others risk factors.
3) Outcome: the outcome of interest is mortality, defined as mortality in children during an episode of ALRI.
4) Design: Observational studies - Cohort studies, case-control and cross-sectional.

In consistency with other authors[5] we will use the term ALRI rather than more specific diagnoses such as pneumonia or bronchiolitis, because in many young children these syndromes are clinically indistinguishable.

We will exclude studies with the following characteristics: a) studies reporting on long-term post-discharge follow up (i.e. more than one year); b) studies reporting selectively on children with very specific co-morbidities such as children with cancer, organ transplant, burns, ventilator-acquired pneumonia, very low birth weight, and articles focusing selectively on new diseases such as avian influenza, SARS or H1N1; c) studies on single micronutrients, as these factors have been explored by intervention trials;[17] d) studies reporting less than five events (i.e five deaths). We will not consider as risk factors signs and symptoms of the disease, except for selected signs (hypoxia, cyanosis, and wheezing).

Search strategy

The following electronic databases will be searched:

- MEDLINE, through Pubmed (from 1956);
- Embase, through OVID (from 1974);
- Global Health Library (WHO web site, no dates restrictions).
- LILACS, through the Virtual Health Library (no dates restrictions);
- Science Citation Index Expanded (SCI-EXPANDED), through Web of Science (from 1992);
- Social Sciences Citation Index (SSCI) through Web of Science (from 1992);

The search strategy has been be verified by an expert librarian, and it is reported in **Annex 1**.

Manual searches of reference lists will also be performed. We will not apply any language restrictions. The team has capacities to assess relevant articles in English, French, Spanish, Portuguese, Slovenian, and German. Attempt will be made to translate any other relevant articles written in other foreign languages, using translation software or other means as more appropriate.

Study selection

Two reviewers (ML and MS) will independently selected potentially eligible studies for inclusion. Disagreements will be solved by discussion. The full text of all eligible citations will be examined in detail.

Assessment of risk of bias in included studies

Two review authors will independently assessed the risk of bias of studies, using the Quality In Prognosis Studies (QUIPS) tool developed by Hayden et al,[18,19] with minor adaptation to our clinical question (**Annex 2**). The tool includes thirty-one items divided in six domains: 1) Study participation; 2) Study attrition; 3) Prognostic factor measurement; 4) Outcome measurement; 5) Study Confounding; 6) Statistical Analysis and reporting.

For each study, each individual item will be graded as a) yes, b) partly, c) no, d) unknown, based on the whether the study will fully complied, partly complied, not comply, or will not report in respect to the characteristic expressed by the item. Based on this evaluation by individual item, each of the six domains will be rated in four categories of risk of bias: 1) Low; 2) Moderate; 3) High; 4) Unknown. The evaluation of the risk of bias will not be based on the study itself, but on the risk of bias in respect of answering our clinical question.

Data Extraction and management

Two review authors will extract data from included studies. Disagreements will be solved by discussion between two review authors, and consensus with a third author. Data from studies will be extracted in a pre-defined form (**Annex 3**), that will be pilot-tested on ten randomly-selected studies, and refined accordingly. The data extraction form contains information regarding: a) the case- definition for ALRI; b) study design; c) sample size, characteristics of the population, setting, and risk factors evaluated; d) confounders considered in the study; e) type of analysis performed (univariate or multivariate); f) study results. Risk factors will be classified as per Wonody et al,[20] with minor modifications.

To avoid mistakes due to data manipulation, we will first collect the data as they are reported and, if any transformation is needed, we will transform them subsequently. Data on measures of relative effect, such as odds ratios (OR) risk ratio or rate ratios (RR), hazard ratios (HR), means and standard deviations (SD), or crude numbers will be extracted.

For studies reporting only RR or HR, when possible we will convert them to OR using a formula to compute OR from RR.[21,22] For studies reporting mean and SD we will calculate the standardized mean difference (SMD) and convert it to OR using a formula to compute OR from SDM.[23] If available in the original studies, we will extract both crude and adjusted OR (or RR/HR). When both univariate and multivariate adjusted models are available, both will be extracted. When more than one adjusted models will be available, we will use the model judged as of better quality, based on a) whether the multivariate analysis was based on a logical

framework and b) whether a hierarchical model was used for the analysis, as for Victora et al.[24,25] Covariates used for adjustment (e.g. age, sex etc) will be recorded in the "data-extraction form" (**Annex 3**). If studies use different categorizations or different cut-off points for the same risk factor, we will note these differences in the forest plots. If a study includes children both under and over 5 years of age data will be extracted only for the group of children under 5 years, whenever this data is available. If sorting is not be possible and at least 80% of the children in a study were under 5 years old, the study will be included; if not it will be excluded. When data is not detailed or clear in the studies, we will make efforts to contact the authors of the studies for all papers published in the lasts five years.

Data analysis and synthesis

When data is available, and meta-analysis is appropriate, we will perform a quantitative synthesis of ORs for mortality across studies for each risk factor. Pooled data will be presented in forest plots. Data that could not be meta-analyzed will be presented in tables and text.

Data will be pooled using the inverse-variance method, in which weight is given to each study according to the inverse of the variance of the effect, to minimise uncertainty about the pooled effect estimates. The random effects model described by Der Simonian and Laird[26] will be used to synthesize data rather than the fixed effect model because it incorporates intra- and inter-study variability. This model was selected a priori as the meta-analysis is expected to include studies with high heterogeneity in both the population and the methods.

Statistical analyses will be performed using Stata v.12; p-values less than 0·05 will be considered statistically significant.

Evaluation of heterogeneity

We will assess the degree of heterogeneity between studies by using visual inspection of the forests plots, and the I-squared (I^2) statistic, with its 95% CI. When $P < 0{\cdot}05$, the presence of heterogeneity will be considered statistically significant, and when $I^2 > 50\%$, the magnitude of heterogeneity will be considered substantial. We will discuss the possible reasons for any heterogeneity. We may use subgroup analyses or meta-regression fo further investigate heterogeneity.

Investigation of heterogeneity

Conducting a large number of subgroup analyses increases the likelihood of false positive results, and therefore it is important to carefully select in advance the relevant characteristics to be investigated.[21]

We will investigate the following potential sources of heterogeneity by subgroup analysis or meta-regression:

1. Definition of ALRI: WHO definition of pneumonia versus other definitions.
2. Setting: community versus hospital.
3. Countries: low income versus lower middle income versus upper middle income countries (as defined by the World Bank[16]).
4. HIV status: studies in populations with low HIV prevalence versus studies in population with high HIV prevalence.
5. Type of study: case-control versus cohort design.
6. Time: studies before and after 2000

These analyses will be considered as secondary analysis, and will not be included in the primary report of this systematic review.

Sensitivity analysis

We will perform the following sensitivity analysis: 1) including in the meta-analyses only the studies with low or moderate risk of bias; 2) substituting the crude ORs with the adjusted ORs (as provided by each study).

Publication bias

We will check for potential publication and small study effects by funnel plot[27] integrating visual inspection of the plot with the test proposed by Egger et al,[28] which, although not completely ideal, it is considered a conservative test, as it can only produce false-positive results.

REFERENCES

1 Liu L, Johnson HL, Cousens S, Perin J, Scott S, et al. (2012) Global, regional, and national causes of child mortality: An updated systematic analysis for 2010 with time trends since 2000. Lancet 379: 2151-61.
2 Nair H, Simoes EA, Rudan I, Gessner BD, Azziz-Baumgartner E, et al. (2013) Global and regional burden of hospital admissions for severe acute lower respiratory infections in young children in 2010: a systematic analysis. Lancet 381: 1380-90.
3 Rudan I, Boschi-Pinto C, Biloglav Z, Mulholland K, Campbell H (2008) Epidemiology and etiology of childhood pneumonia. Bull World Health Organ 86: 408-16.
4 Walker CL, Rudan I, Liu L, Nair H, Theodoratou E, et al. (2013) Global burden of childhood pneumonia and diarrhoea. Lancet 381: 1405-16.
5 Unicef (2012) Pneumonia and diarrhoea. Tackling the deadliest diseases for the world's poorest children. New York. Available at http://www.childinfo.org/files/Pneumonia_Diarrhoea_2012.pdf, accessed 2th July 2013.
6 Jackson S, Mathews KH, Pulanic D, Falconer R, Rudan I, et al. (2013) Risk factors for severe acute lower respiratory infections in children: a systematic review and meta-analysis. Croat Med J 54: 110-21.
7 Wonodi CB, Deloria-Knoll M, Feikin DR, DeLuca AN, Driscoll AJ, et al. (2012) Evaluation of risk factors for severe pneumonia in children: The pneumonia etiology research for child health study. Clinical Infectious Diseases 54 Suppl 2: S124-S131.
8 WHO Collaborative Study Team on the Role of Breastfeeding on the Prevention of Infant Mortality (2000) Effect of breastfeeding on infant and child mortality due to infectious diseases in less developed countries: a pooled analysis. Lancet 355: 451-5.
9 Lozano JM (2001) Epidemiology of hypoxaemia in children with acute lower respiratory infection. Int J Tuberc Lung Dis 5: 496-504.
10 Chisti MJ, Tebruegge M, La Vincente S, Graham SM, Duke T (2009) Pneumonia in severely malnourished children in developing countries - mortality risk, aetiology and validity of WHO clinical signs: a systematic review. Trop Med Int Health 14: 1173-89.
11 Rice AL, Sacco L, Hyder A, Black RE (2000) Malnutrition as an underlying cause of childhood deaths associated with infectious diseases in developing countries. Bull World Health Organ 78: 1207-21.
12 Dherani M, Pope D, Mascarenhas M, Smith KR, Weber M, et al. (2008) Indoor air pollution from unprocessed solid fuel use and pneumonia risk in children aged under five years: a systematic review and meta-analysis. Bull World Health Organ 86: 390-398C.
13 Gilbert K, Fine MJ (1994) Assessing prognosis and predicting patient outcomes in community-acquired pneumonia. Semin Respir Infect 9: 140-52.
14 Liberati A, Altman DG, Tetzlaff J, Mulrow C, Gotzsche PC, et al. (2009) The PRISMA statement for reporting systematic reviews and meta-analyses of studies that evaluate health care interventions: explanation and elaboration. PLoS Med 6: e1000100.
15 Stroup DF, Berlin JA, Morton SC, Olkin I, Williamson GD, et al. (2000) Meta-analysis of observational studies in epidemiology: a proposal for reporting. Meta-analysis Of Observational Studies in Epidemiology (MOOSE) group. JAMA 283: 2008-12.
16 The World Bank (2013) Country and Lending Groups. Historical classification. Available: http://data worldbank org/about/country-classifications/a-short-history Accessed 6 September 2013.
17 Roth DE, Caulfield LE, Ezzati M, Black RE (2008) Acute lower respiratory infections in childhood: opportunities for reducing the global burden through nutritional interventions. Bull World Health Organ 86: 356-64.
18 Hayden JA, Cote P, Bombardier C (2006) Evaluation of the quality of prognosis studies in systematic reviews. Ann Intern Med 144: 427-37.
19 Hayden JA, van der Windt DA, Cartwright JL, Cote P, Bombardier C (2013) Assessing bias in studies of prognostic factors. Ann Intern Med 158: 280-6.

20 Wonodi CB, Deloria-Knoll M, Feikin DR, DeLuca AN, Driscoll AJ, et al. (2012) Evaluation of risk factors for severe pneumonia in children: The pneumonia etiology research for child health study. Clinical Infectious Diseases 54 Suppl 2: S124-S131.
21 Deeks JJ, Higgins JP, Altman DG (2011) Analysing data and undertaking meta-analyses. In: Higgins JP, Green S, editors. Cochrane Handbook for Systematic Reviews of Interventions Version 5.1.0. [updated March 2011]. The Cochrane Collaboration 2011. Available at www.cochrane-handbook.org, accessed 3rd July 2013.
22 Schunemann HJ, Oxman AD, Vist GE, Higgins JP, Deeks JJ, et al. (2011) Interpreting results and drawing conclusions. In: Higgins JP, Green S, editors. Cochrane Handbook for Systematic Reviews of Interventions Version 5.1.0. [updated March 2011]. The Cochrane Collaboration, 2011. Available at www.cochrane-handbook.org, accessed 3rd July 2013
23 Chinn S (2000) A simple method for converting an odds ratio to effect size for use in meta-analysis. Stat Med 19: 3127-31.
24 Victora CG, Fuchs SC, Flores JA, Fonseca W, Kirkwood B (1994) Risk factors for pneumonia among children in a Brazilian metropolitan area. Pediatrics 93: 977-85.
25 Victora CG, Huttly SR, Fuchs SC, Olinto MT (1997) The role of conceptual frameworks in epidemiological analysis: a hierarchical approach. Int J Epidemiol 26: 224-7.
26 DerSimonian R, Laird N (1986) Meta-analysis in clinical trials. Control Clin Trials 7: 177-88.
27 Sterne J, Egger M, Moher D (2011) Addressing reporting biases. In: Higgins JP, Green S, editors. Cochrane Handbook for Systematic Reviews of Interventions Version 5.1.0. The Cochrane Collaboration, 2011 [updated March 2011]. Available at www.cochrane-handbook.org, accessed 3rd July 2013
28 Egger M, Davey SG, Schneider M, Minder C (1997) Bias in meta-analysis detected by a simple, graphical test. BMJ 315: 629-34.

TABLE 1. Factors that may modify the risk of death in children with ALRI *

**CHILD-RELATED**	▪ Age<br>▪ Sex<br>▪ Birth -weight<br>▪ Gestational age/prematurity<br>▪ Mode/place of delivery<br>▪ Birth order<br>▪ Breastfeeding practices<br>▪ Nutritional status<br>▪ Vaccinations<br>▪ Co-morbidities, acute and chronic
**FAMILY-RELATED**	▪ Parental age<br>▪ Parental education<br>▪ Parental health status<br>▪ Mother access to antenatal care<br>▪ Family planning, birth spacing<br>▪ Unwanted pregnancy<br>▪ Respiratory infections in others members of the family, including exposure to tuberculosis in household<br>▪ Vaccinations<br>▪ Family structure<br>▪ Care-seeking behaviours
**DISEASE-RELATED**	▪ Disease clinical severity<br>▪ Disease duration<br>▪ Disease aetiology<br>▪ Hypoxia<br>▪ Chest X-ray characteristics<br>▪ Laboratory characteristics<br>▪ others
**ENVIRONMENTAL**	▪ Indoor air pollution<br>▪ Passive smoke exposure<br>▪ Home crowding level<br>▪ Water, sanitation and hygiene<br>▪ Seasonality, clime<br>▪ Setting (rural vs urban)
**SOCIO-ECONOMICAL**	▪ Income<br>▪ Occupation<br>▪ Type of house and commodities<br>▪ Water, sanitation and hygiene practices
**HEALTH-SERVICES FACTORS**	▪ Access to health care<br>▪ Quality and type of the health service<br>▪ Availability of drugs and supplies<br>▪ Quality of the case-management (including case identification, referral, treatment, discharge and follow up)<br>▪ Antibiotics prior of hospitalisation<br>▪ others

* from Wonody et al.[20] with minor modifications

ANNEX 1: Search strategy

MEDLINE (Pubmed)
((("pneumonia"[MeSH Terms] OR pneumonia[Text Word] OR "respiratory infection"[All Fields] OR "respiratory infections"[All Fields] OR "lower respiratory infection"[All Fields] OR "lower respiratory tract infections"[All Fields] OR "Bronchiolitis"[Mesh] OR "Bronchiolitis, Viral"[Mesh] OR bronchiolitis [All Fields]) AND ("child"[MeSH Terms] OR children[Text Word] OR "pediatrics"[MeSH Terms] OR "paediatrics"[All Fields] OR "paediatric"[All Fields] OR "Infant"[Mesh] OR infant [Text Word] OR newborn[Text Word] OR newborns [Text Word] OR neonate[Text Word] OR neonates [Text Word] OR infants [Text Word]) AND ("risk factors"[MeSH Terms] OR "risk factor"[Text Word] OR "risk factors"[All Fields] OR determinant[All Fields] OR determinant[All Fields] OR determinants[All Fields] OR predictor[All Fields] OR predictors[All Fields] OR "predictive value"[All Fields] OR "risk"[MeSH Terms] OR "risk"[All Fields]) AND ("mortality"[Subheading] OR "mortality"[MeSH Terms] OR mortality[Text Word] OR "death"[MeSH Terms] OR "death"[All Fields] OR fatality[All Fields] OR fatal[All Fields] OR ("death"[MeSH Terms] OR "death"[All Fields] OR "deaths"[All Fields])))

EMBASE
1 pneumonia/co, dm, dt, ep, et, pc, th [Complication, Disease Management, Drug Therapy, Epidemiology, Etiology, Prevention, Therapy]
2 (children or infant*OR childhood or preschool*).mp. [mp=title, abstract, subject headings, heading word, drug trade name, original title, device manufacturer, drug manufacturer, device trade name, keyword]
3 limit 2 to human
4 1 and 3
5 developing countries.mp. or developing country/
6 (Asia* or Africa* or South America).mp. [mp=title, abstract, subject headings, heading word, drug trade name, original title, device manufacturer, drug manufacturer, device trade name, keyword]
7 LMIC.mp.
8 "low and middle income countries".mp.
9 5 or 6 or 7 or 8
10 4 and 9

GLOBAL HEALTH LIBRARY (WHO web site)
1 pneumonia OR "respiratory infection" OR "respiratory infections" OR Neumoni$ OR Infeccao Respiratoria OR Infeccion Respiratoria OR Infeccoes Respiratorias OR Infecciones Respiratorias
2 child$ OR infant$ OR pediatric$ OR paediatric$ OR Nino$ OR Crianca$ OR infant$ OR Pediatric$ OR newborn$ OR neonat$ OR "recien nacido" OR "recem nacido"
3 "risk factors" OR "risk factor" OR determinant$ OR predict$ OR risk$
4 (mortality OR death$ OR fatal$ OR letal$ OR outcome$ OR mortalidad OR mortalidade OR muerte OR Morte OR desenlace$ OR obito$
1 AND 2 AND 3 AND 4

LILACS (Virtual Health Library)
(pneumonia OR "respiratory infection" OR "respiratory infections" OR Neumoní$ OR Infecção Respiratoria OR Infección Respiratoria OR Infecçoes Respiratorias OR Infecciones Respiratorias OR bronchiolitis) (child$ OR infant$ OR pediatric$ OR paediatric$ OR Niño OR ninos OR Criança OR Crianças OR infantil OR infantiles OR Pediátrico OR Pediátricos) (mortality OR death$ OR fatal$ OR letal$ OR outcome$ OR mortalidad OR mortalidade OR muerte OR Morte OR desenlace$) 3009

SCI-EXPANDED and SSCI (Web of Science)

1 TS=(pneumonia OR “respiratory infection” OR “respiratory infections” OR "lower respiratory tract infections" OR "bronchiolitis")
2 TS=(child* OR pediatric* OR paediatric* OR infant* OR newborn* or neonat*)
3 TS=(“risk factors” OR “risk factor” OR predictor* OR risk* OR determinant* OR "predictive value")
4 TS=(mortality OR death* OR fatal*)
5 1 AND 2 AND 3 AND 4

ANNEX 2: Modified Quality In Prognosis Studies (QUIPS) tool

**ASSESSMENT FOR RISK OF BIAS**				
First author	Reviewer.......................			
**Biases**	**Issues to consider for judging overall rating of "Risk of bias"**	**Study Methods and Comments**	**Rating of reporting**	**Rating of risk of bias**
Assess the risk of each potential bias	These issues will guide your thinking and judgement about the overall risk of bias within each of the 6 domains.	Provide comments or excerpts to facilitate the consensus process that will follow	Y: yes<br>N: no<br>P: partial<br>U: unknown<br>NA: not applicable	HIGH<br>MODERATE LOW<br>UNKNOWN
**1) STUDY PARTICIPATION**	The study sample adequately represents the population of interest<br>(i.e. The relationship between RF and death from ALRI should be the same for participants and eligible non-participants)			SUMMARY
a. Adequate participation in the study by eligible persons (>80%)				
b. Description of the source population or population of interest				
c. Description of the baseline study sample				
d. Adequate description of the sampling frame and recruitment.				
e. Adequate description of the period and place of recruitment				
f. Adequate description of inclusion and exclusion criteria				
**2) STUDY ATTRITION**	The study data available (i.e. participants not lost to follow-up) adequately represent the study sample<br>(i.e. The relationship between RF and death from ALRI should be the same for completing			SUMMARY

	and non-completing participants)		
a. Adequate response rate for study participants *(> 80%)*			
b. Description of attempts to collect information on participants who dropped out			
c. Reasons for loss to follow-up are provided			
d. Adequate description of participants lost to follow-up			
e. There are no important differences between participants who completed the study and who did not			
**3) PROGNOSTIC FACTORS MEASUREMENT**	The PF is measured in a similar way for all participants (i.e. The measurement of the RF should be the same for children who died and for those who survived)		SUMMARY
a. A clear definition or description of the PF is provided			
b. Method of PF measurement is adequately valid and reliable (i.e. direct ascertainment; secure record, hospital record)			
c. Continuous variables are reported or appropriate cut points are used			
d. The method and setting of measurement of PF is the same for all study participants			
e. Adequate proportion of the study sample has complete data for the PF *(> 80%)*			
f. Appropriate methods of imputation are used for missing PF data			
**4) OUTCOME MEASUREMENT**	The outcome of interest is measured in a similar way for all participants (i.e. The definition and ascertainment of ALRI should be the same for children who died and survivors, and for children with and without RF;		SUMMARY

	for case-control studies treat the case definition as the outcome measure)		
a. A clear definition of the outcome of interest is provided (including time of death)			
b. Method of outcome measurement used is adequately valid and reliable (i.e. independent blind assessment, hospital record or record linkage)			
c. The method and setting of outcome measurement is the same for all study participants			
**5) STUDY CONFOUNDING**	Important potential confounder are appropriately accounted for (The observed effect of the RF on the death from ALRI should not be distorted by another factor related to the RF and the outcome)		SUMMARY
a. *Most* important confounders are measured			
b. Clear definitions of the important confounders measured are provided			
c. Measurement of all important confounders is adequately valid and reliable			
d. The method and setting of confounding measurement are the same for all study participants			
e. Appropriate methods are used if imputation is used for missing confounder data			
f. Important potential confounders are accounted for in the study design (by limiting the study to specific population groups, or by matching)			
g. Important potential confounders are accounted for in the analysis *(by stratification, multivariate regression)*			
**6) STATISTICAL**	The statistical analysis is appropriate, and all		SUMMARY

**ANALYSIS AND PRESENTATION**	primary outcomes are reported		
a. Sufficient presentation of data to assess the adequacy of the analytic strategy			
b. Strategy for model building is appropriate and is based on a conceptual framework or model			
c. The selected statistical model is adequate for the design of the study			
d. There is no selective reporting of results (*based on the study protocol, if available, or on the method section*)			

ANNEX 3

Data extraction sheet<br>RISK FACTORS FOR DEATH FROM ALRI IN CHILDREN LESS THAN 5 YEARS OF AGE

AUTHOR AND YEAR OF STUDY…………………………………………………….

Country..
Period of Study..................................

Objectives of the study (in authors' words)

____

____

STUDY DESIGN

- **Cohort/case control/Case series/Others: ________________________________**

Notes on methodology

____

____

SETTING

- **Community/ hospital**
- **Urban / rural**
- **Other characteristics:** ____

CASE DEFINITION

____

____

STUDY POPULATION

- Total N:
- N considered for ALRI:
- Inclusion Criteria:
- Exclusion criteria:
- Age:
- % males:
- Absolute number of death and Case Fatality Rate (CFR): ______________________
- Other characteristics: ____

ANALYZED RISK FACTORS Expand as needed

**#**	**Risk factor**	**Sample**	**Notes**
**1**			
**2**			
**3**			
**4**			
**5**			
**6**			

Confounders

1.____
2.____

3.____
4.____
5.____

Notes on possible source of bias:
1.____
2.____
3.____
4.____
5.____

Other notes:

RESULTS Expand as needed

RF	death	alive	total	u.or.rr	IC		a.or.rr		

	death	alive	total	u.or.rr	IC		a.or.rr		

	death	alive	total	u.or.rr	IC		a.or.rr		

	death	alive	total	u.or.rr	IC		a.or.rr		

	death	alive	total	u.or.rr	IC		a.or.rr		

	death	alive	total	u.or.rr	IC		a.or.rr		

	death	alive	total	u.or.rr	IC		a.or.rr		

	death	alive	total	u.or.rr	IC		a.or.rr		
