# Supplementary material for: Risk Factors for Mortality from Acute Lower Respiratory Infections (ALRI) in Children under Five Years of Age in Low and Middle-Income Countries: A Systematic Review and Meta-Analysis of Observational Studies
Source: PLoS One. 2015 Jan 30;10(1):e0116380. doi: 10.1371/journal.pone.0116380 (PMC4312071; doi:10.1371/journal.pone.0116380)
Supplement: S1 Forest Plots — (PDF) [file pone.0116380.s002.pdf]

S1 Forest plots

Forest plots of the association between different risk factors and death from ALRI, and results from studies that could not be meta-analysed

List of figures

		Page
Figure 1	Association between age less than one year and death from ALRI	3
Figure 2	Association between younger versus older age in infants and death from ALRI	4
Figure 3	Association between female sex and death from ALRI	5
Figure 4	Association between prematurity and death from ALRI	6
Figure 5	Association between birth order and death from ALRI	7
Figure 6	Association between low birth weight and death from ALRI	8
Figure 7	Association between severe malnutrition and death from ALRI	9
Figure 8	Association between moderate malnutrition and death from ALRI	10
Figure 9	Association between inadequate breastfeeding and death from ALRI	11
Figure 10	Association between HIV/AIDS and death from ALRI	12
Figure 11	Association between comorbidity with chronic diseases and death from ALRI	13
Figure 12	Association between malaria and death from ALRI	14
Figure 13	Association between diarrhea/dehydration and death from ALRI	15
Figure 14	Association between diagnosis of measles and death from ALRI	16
Figure 15	Association between previous ALRI and death from ALRI	17
Figure 16	Association between severe pneumonia and death from ALRI	18
Figure 17	Association between very severe pneumonia and death from ALRI	19
Figure 18	Association between diagnosis of Respiratory Syncytial Virus and death from ALRI	20
Figure 19	Association between diagnosis of Pneumocyistis Carinii and death from ALRI	21
Figure 20	Association between mother's age and death from ALRI	22
Figure 21	Association between mother's low educational level and death from ALRI	23
Figure 22	Association between mother's paid job and death from ALRI	24
Figure 23	Association between socioeconomic status and death from ALRI	25
Figure 24	Association between sanitation and water, and death from ALRI	26
Figure 25	Association between crowding and death from ALRI	27
Figure 26	Association between second-hand smoke and death from ALRI	28
Figure 27	Association between indoor pollution and death from ALRI	29
Figure 28	Association between seasonality and death from ALRI	30
Figure 29	Association between setting of residence and death from ALRI	31
Figure 30	Association between immunization and death from ALRI	32
Figure 31	Association between antenatal good practices and death from ALRI	33
Figure 32	Association between pre-hospital care and death from ALRI	34

List of abbreviations

ALRI	=	Acute Lower Respiratory Disease
CAR	=	Central African Republic
DRC	=	Democratic Republic of Congo
PNG	=	Papua New Guinea
WHO	=	World Health Organization
OR	=	Odds Ratio
aOR	=	adjusted Odds Ratio
HR	=	Hazard Ratio
RR	=	Risk Ratio
SD	=	Standard Deviation
IQR	=	Interquantile Range

Figure 1 Association between age less than one year and death from ALRI (28 studies; 63,629 children)

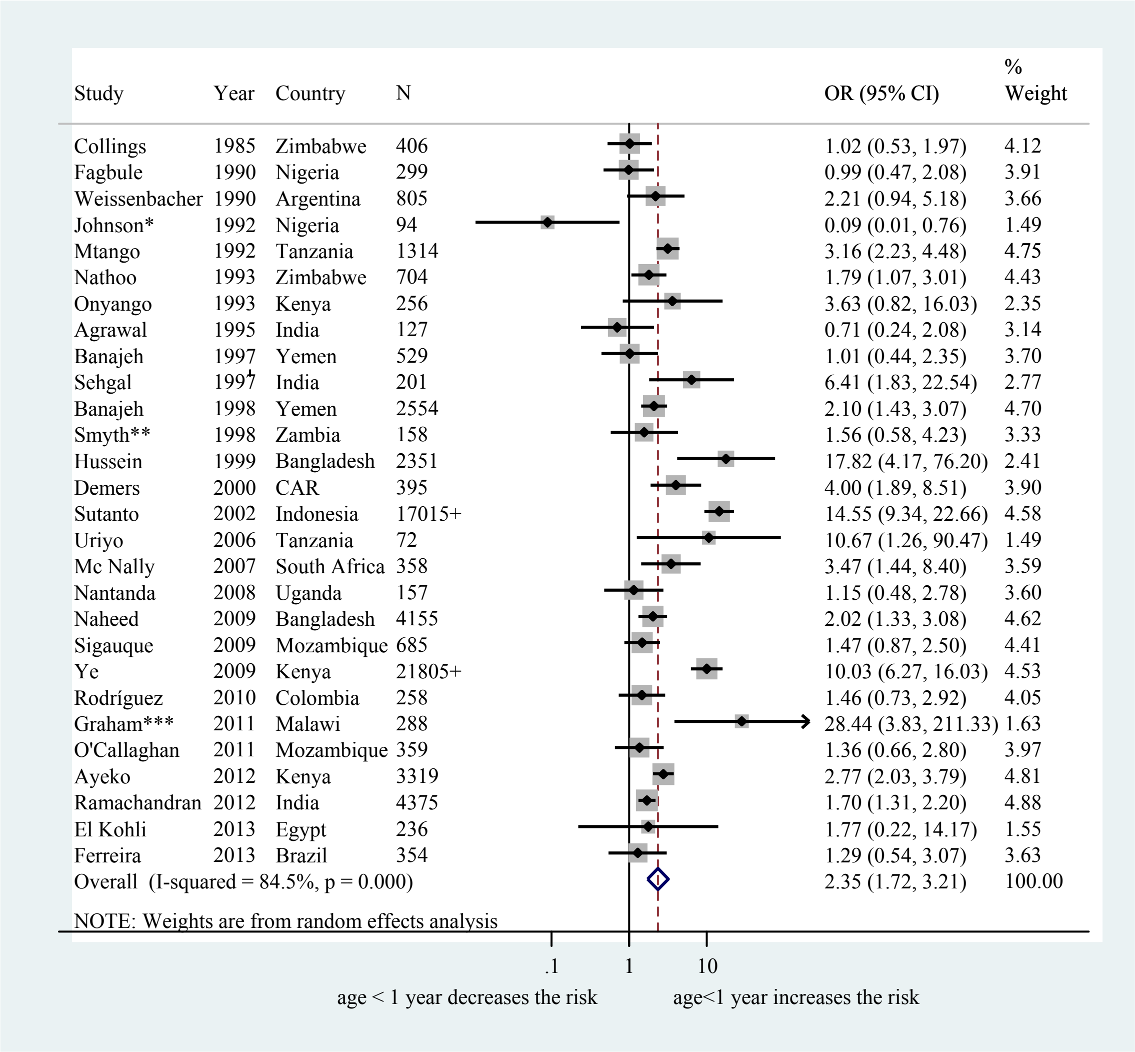

* includes Johnson "Host factors.." 1992
** includes Smyth 1997
*** includes Ramakrishna 2012
+ children-years

Other studies not included in the forest plot

- Bahwere 2004:<24 month vs 2-5 years: OR=1.57 (1.01-2.42)
- Djelantik: 2003: <4 months vs >4 months: OR: 4.18 (3.45-5.30)
- Chisti 2011: median age(IQ) 8.3 months (5.5-11.5) in children who died vs 5 months (2.9-12) in survivors
- Chisti 2013 median age(IQ): 8 months (4.9-11.0) in children who died vs 9.7 months (5.0-18.0) in survivors
- Delport 2002: median age: 15 weeks in children who died, 5 weeks in survivors
- Johnson 2008: non significant (p=0.65) difference of mean between children who died and survivors

Figure 2 Association between younger versus older age in infants and death from ALRI (20 studies; 22,006 children)

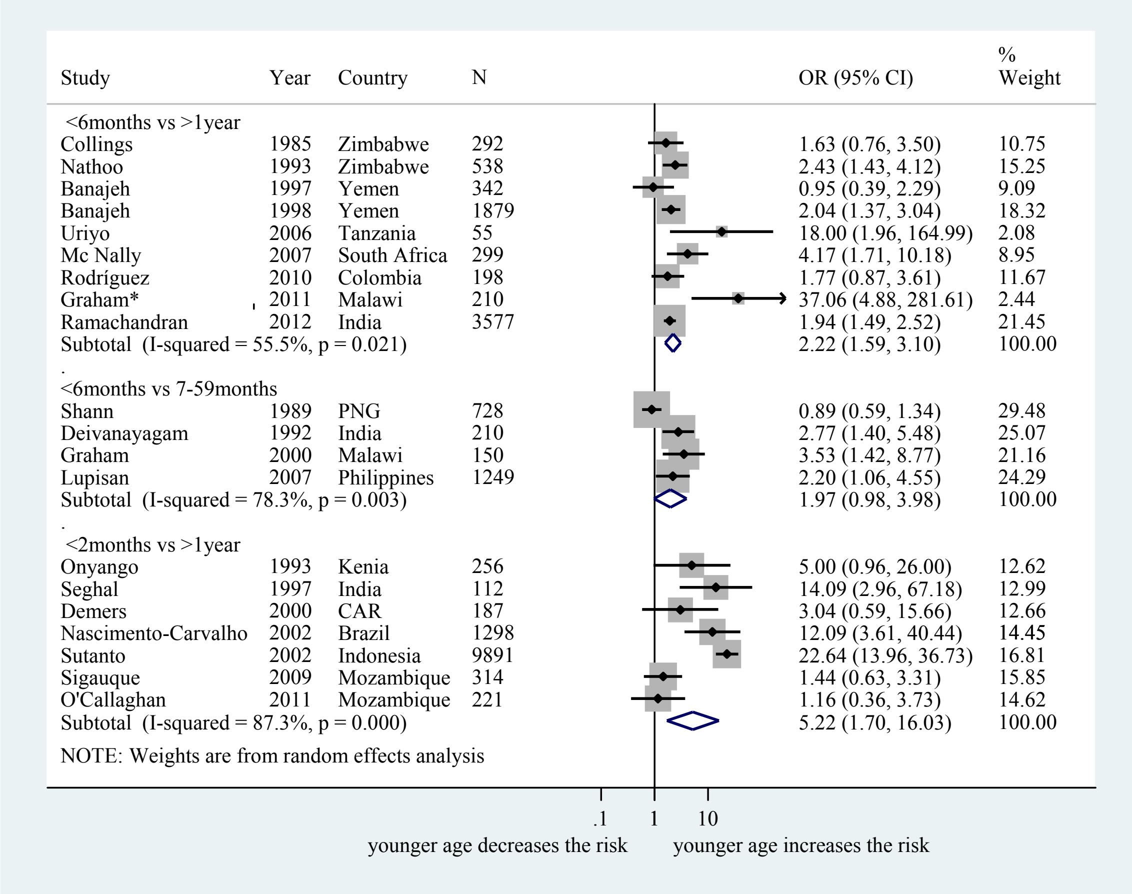

*includes Ramakrishna 2012

Figure 3 Association between female sex and death from ALRI (23 studies; 20,385 children)

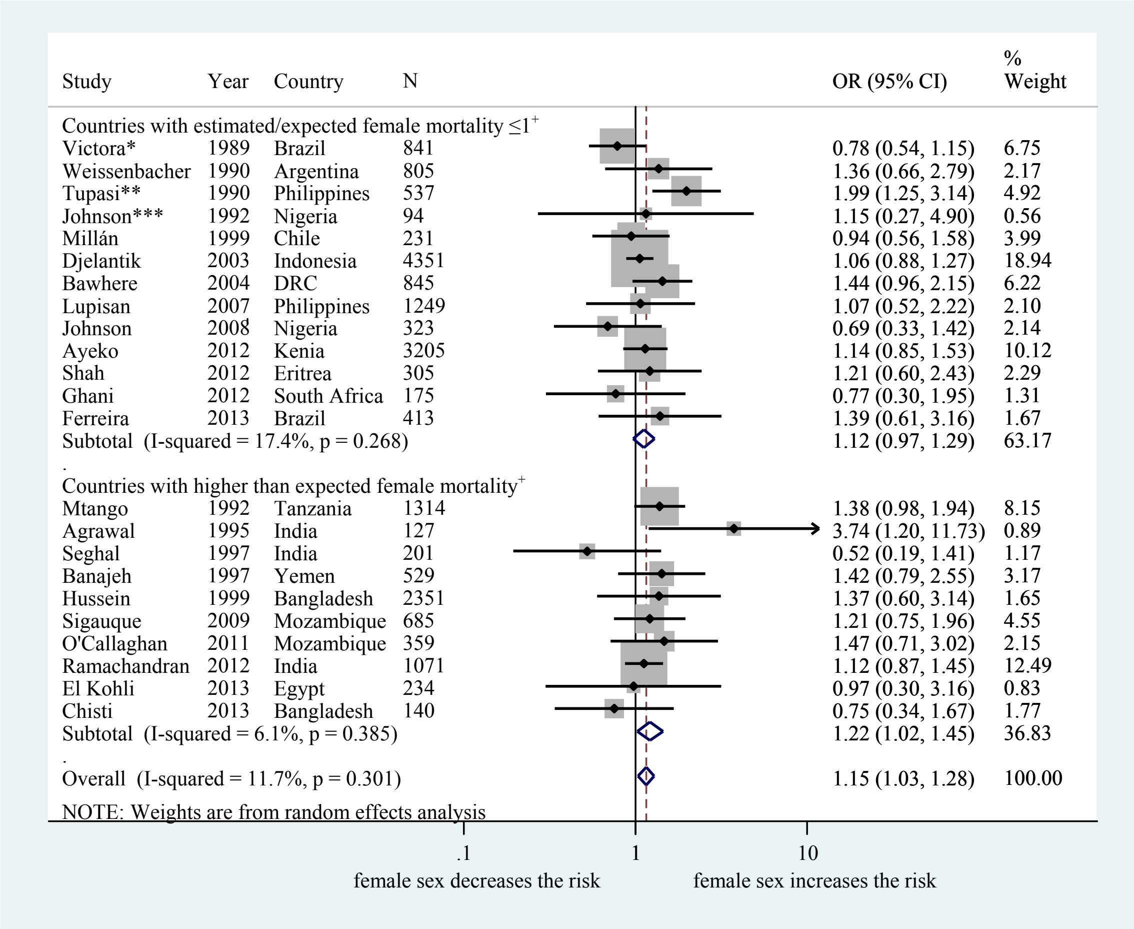

[+] as estimated by Alkema et al. (Alkema L, Chao F, You D, Pedersen J, Sawyer CC. National, regional, and global sex ratios of infant, child, and under-5 mortality and identification of countries with outlying ratios: a systematic assessment. Lancet Glob Health 2014; 2: e521-e530)

* includes Victora 1988

** includes Tupasi “Etiology...” 1990

*** includes Johnson “Host factors...” 1992

Figure 4 Association between prematurity and death from ALRI (6 studies; 2,983 children)

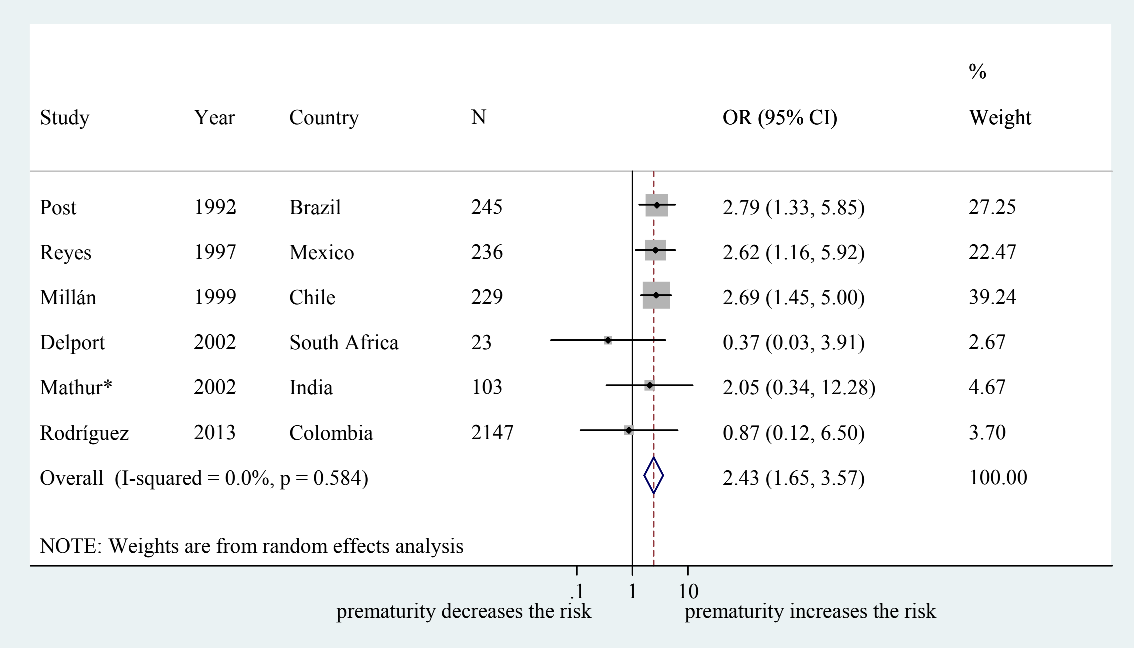

*adjusted OR

Other studies not included in the forest plot

Shah 2012: OR for each one week increase in gestational age: =0.31 (0.18-0.73)

Figure 5 Association between birth order and death from ALRI (6 studies; 1,783 children)

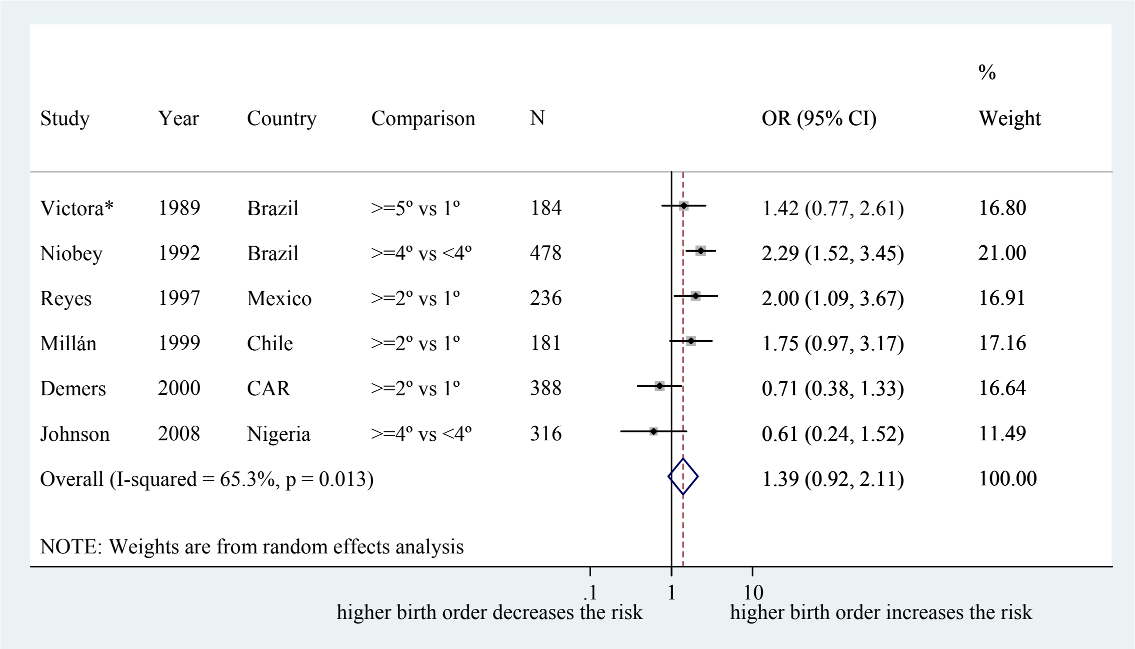

* includes Victora 1988

Figure 6 Association between low birth weight and death from ALRI (9 studies; 7973 children)

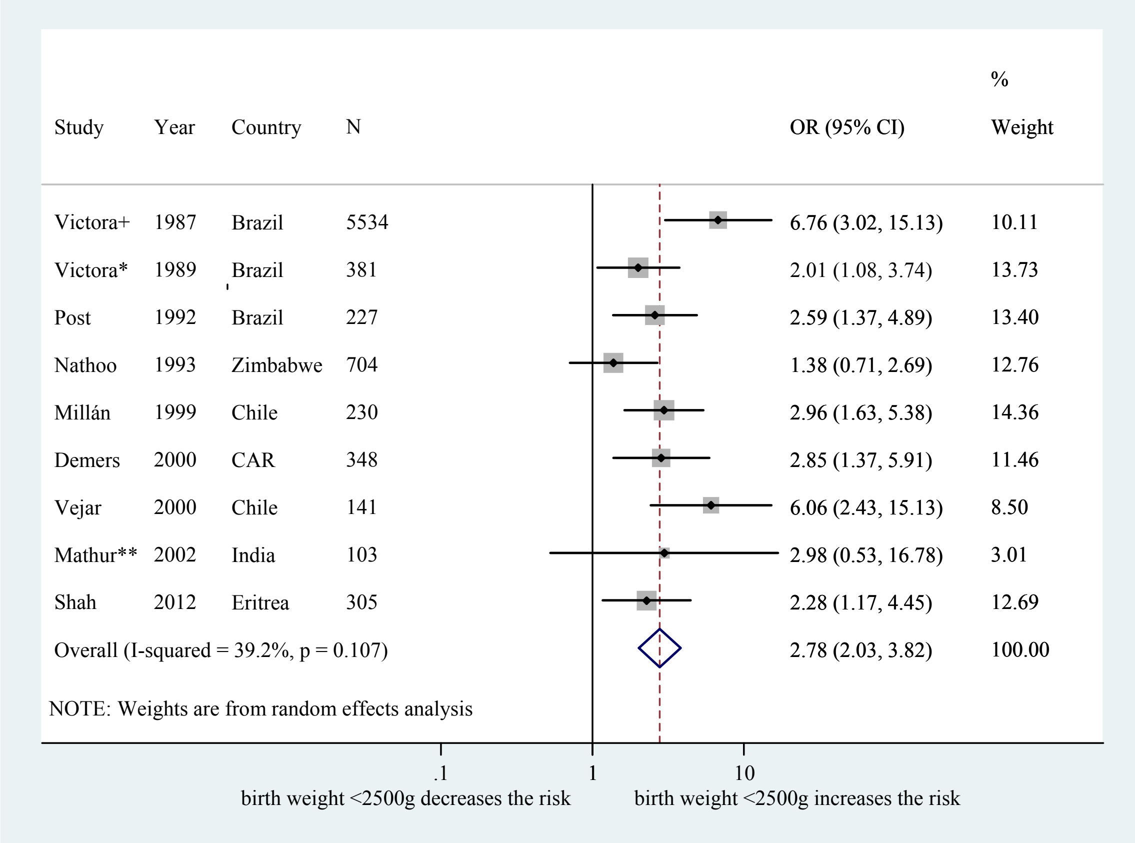

*includes Victora 1988
** adjusted OR
+ infant-years

Other studies not included in the forest plot
Arifeen 2001: birth-weight< 2500g vs >2500g: RR: 2.21 (1.54-3.17)
Niobey 1992: OR for each 100g increase in birth-weight: 0.36 (0.26-0.48)
Lehmann 1996: HR for each 1kg reduction in birth-weight: 2.1 (1.19-3.73)

Figure 7 Association between severe malnutrition and death from ALRI (21 studies; 13,478 children)

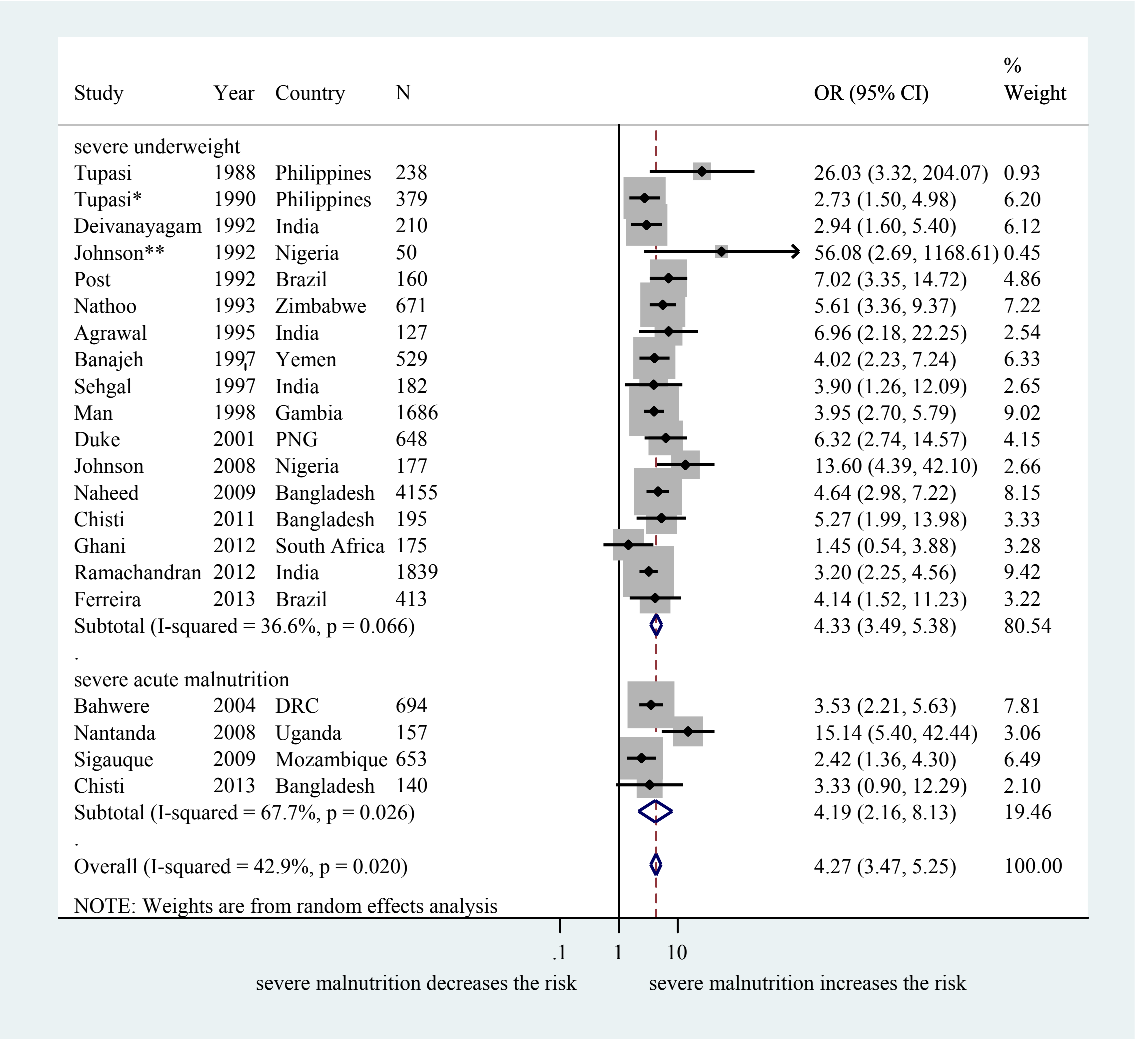

* includes Tupasi etiology 1990

** includes Johnson "Host factors.." 1992

Figure 8 Association between moderate malnutrition and death from ALRI (18 studies; 13,608 children)

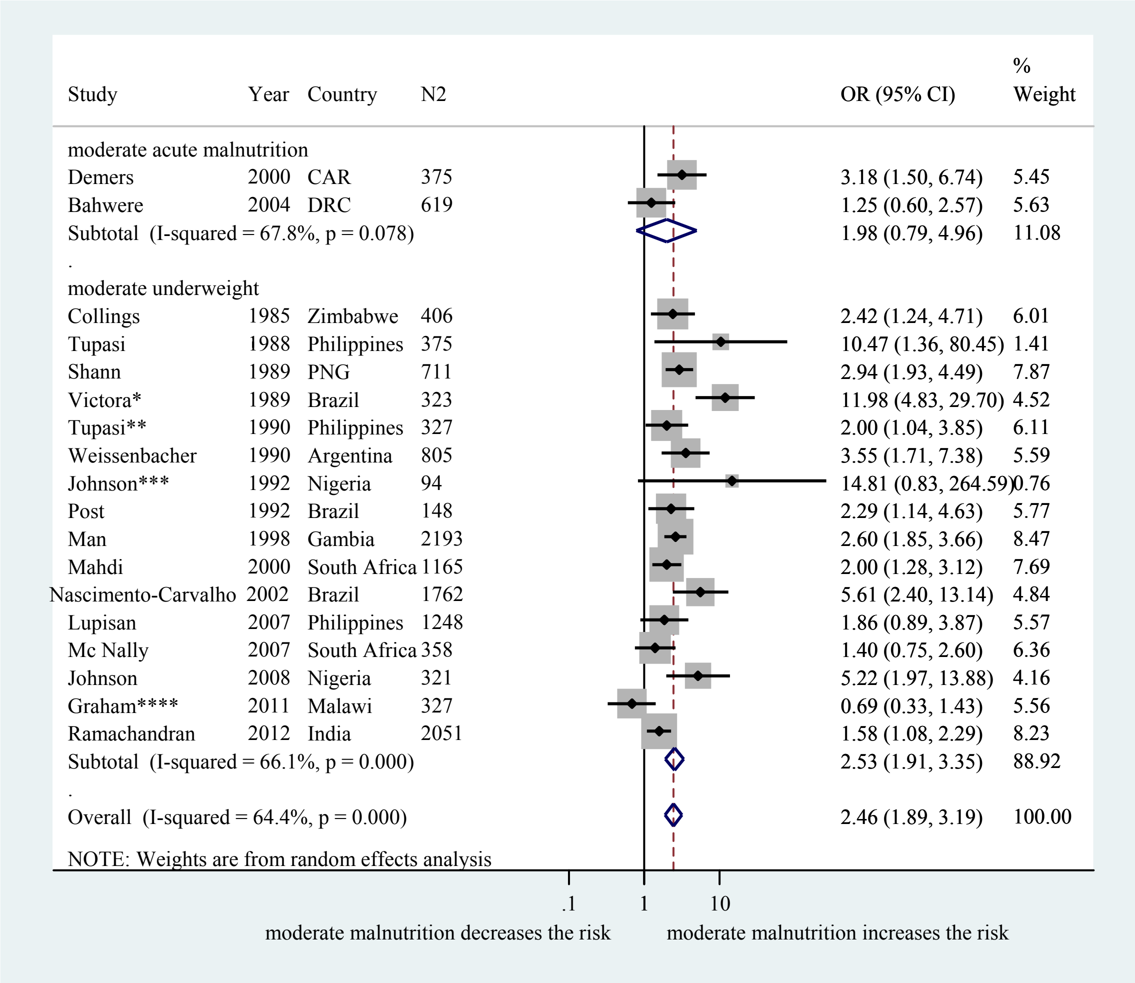

* includes Victora 1988
** includes Tupasi etiology 1990
*** includes Johnson “Host factors..” 1992
**** includes Ramakrishna 2012

Other studies not included in the forest plot

- De Francisco 1993: poor weight progress versus good weight progress: OR 5.26 (1.82-14.29)
- Smyth 1998: multivariate coefficient for weight for age: -0.652 (p=0.0073)
- Yoon 1997: HR for for 1standard deviation reduction in weight-for-age z score: 1.8 (1.4-2.4)
- Djelantik 2003: <5º percentile vs >5º: OR =1.12 (0.92-1.36)
- Vejar 2000: < -1SD vs >1SD: OR= 30.6 (3.9-64.5)
- Rodríguez 2010: <3º percentile vs >3º = OR= 1.69 (0.66-4.33)

Figure 9 Association between inadequate breastfeeding and death from ALRI (12 studies, 14 comparisons; 3,941 children)

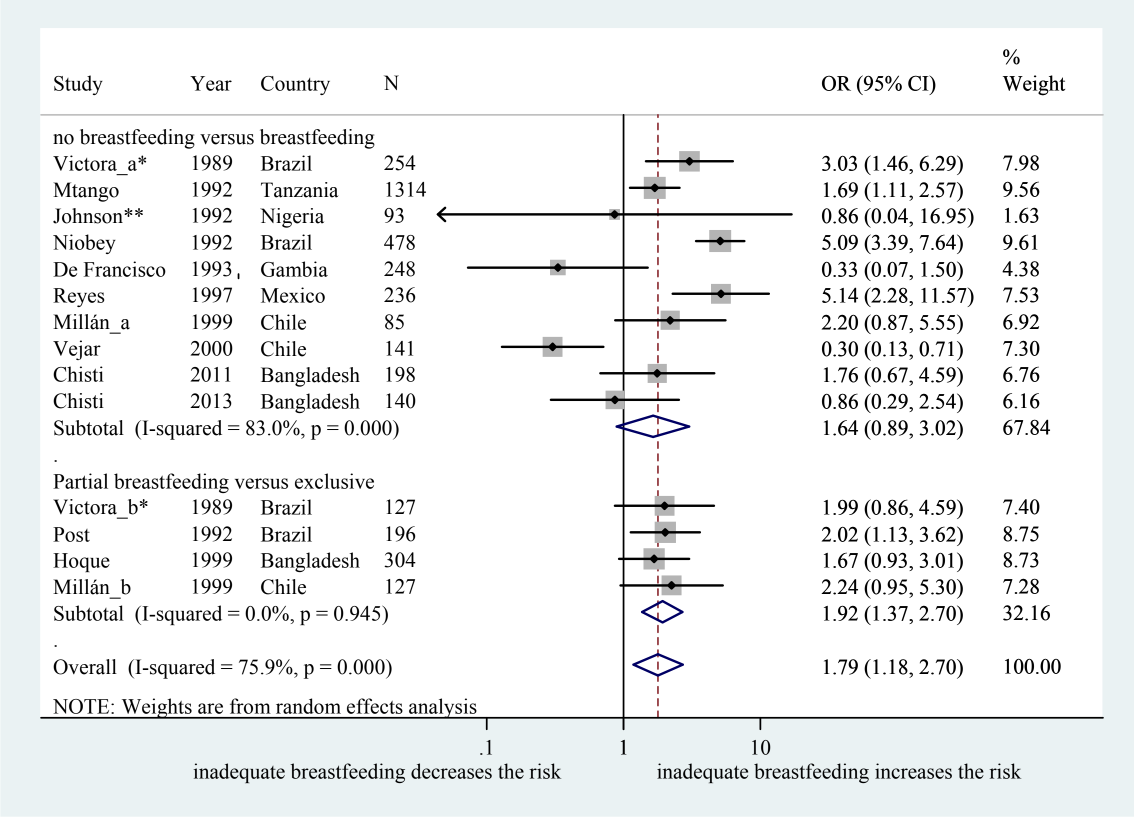

* includes Victora 1988
** includes Johnson "Host factors..." 1992

Other studies not included in the forest plot

Arifeen 2001: partial or no breastfeeding vs exclusive breastfeeding HR: 2.4 (1.14 – 5.04)
Bahl 2005: no breastfeeding vs predominant breastfeeding HR : 38.9 (8.37-180.4)
Yoon 1996: no breastfeeding vs breastfeeding (immediately prior to the illness) in ALRI

- 0-5 months: HR=0.4 (0.1 -1.9)
- 6-11 months: HR=2.2 (0.75-3.6)
- 12-23 months: HR=0.8 (0.15-4.6)

Yoon 1996: no breastfeeding vs breastfeeding (immediately prior to the illness) in ALRI associated with diarrhoea

- 0-5 months: HR=5.7 (1.8 -18.4)
- 6-11 months: HR=0.9 (0.35-2.3)
- 12-23 months: HR=7.2 (0.87-59.5)

Figure 10 Association between HIV/AIDS and death from ALRI (14 studies; 4,171 children)

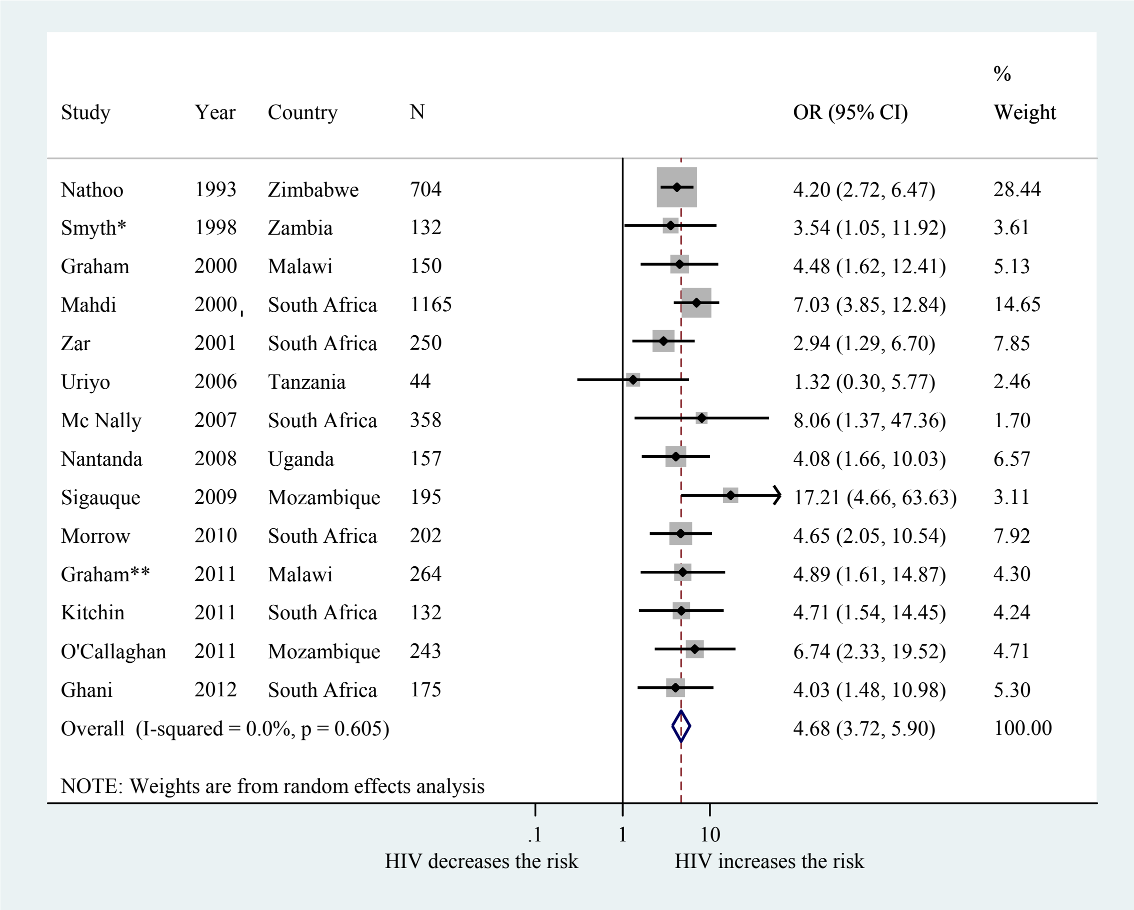

* includes Smyth 1997

** includes Ramakrishna 2012

Figure 11 Association between comorbidity with chronic diseases and death from ALRI (12 studies; 7,473 children)

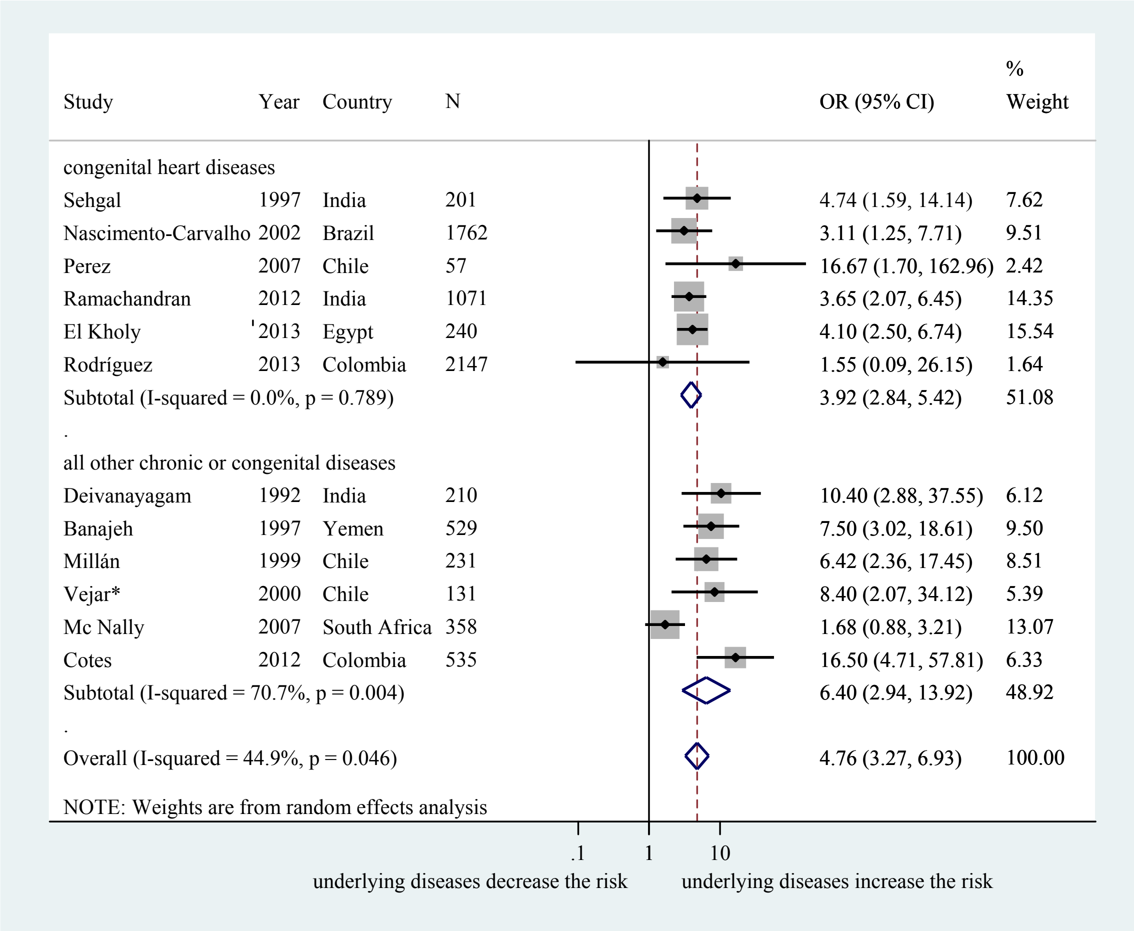

*Vejar: aOR

Figure 12 Association between malaria and death from ALRI (3 studies; 3,991 children)

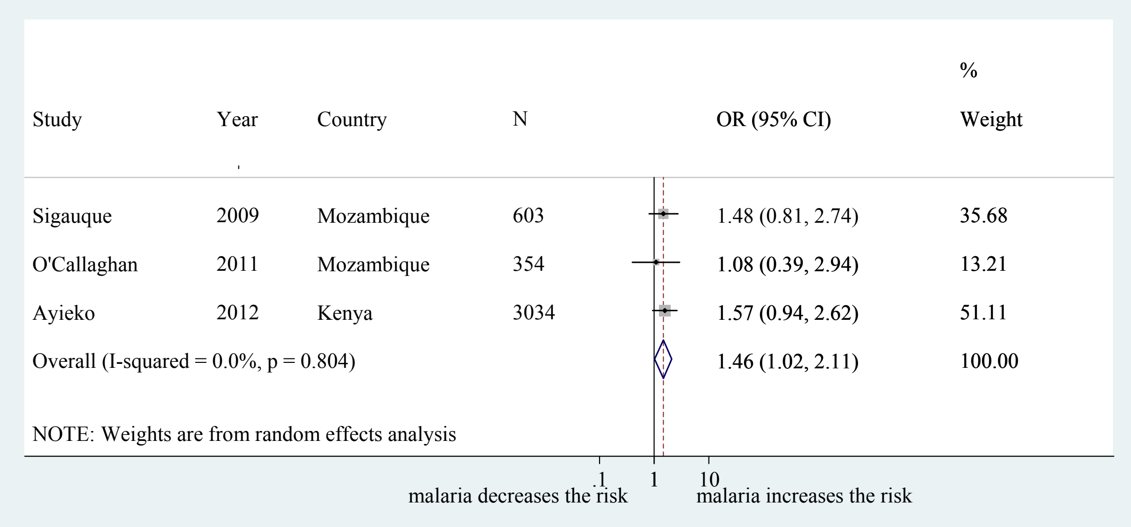

Other studies not included in the forest plot

Smyth 1998: malaria non significant for PN mortality (p= 0.3692)

Figure 13 Association between diarrhea/dehydration and death from ALRI (6 studies; 5,570 children)

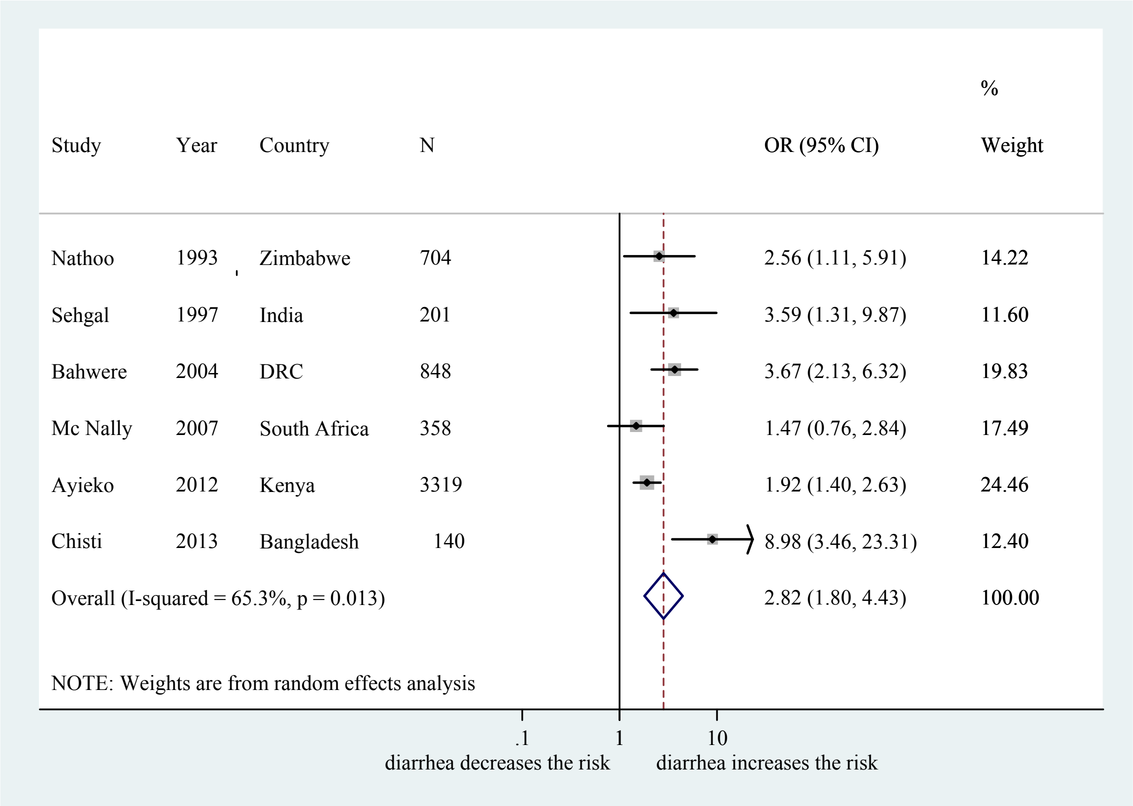

Figure 14 Association between diagnosis of measles and death from ALRI (6 studies; 2,420 children)

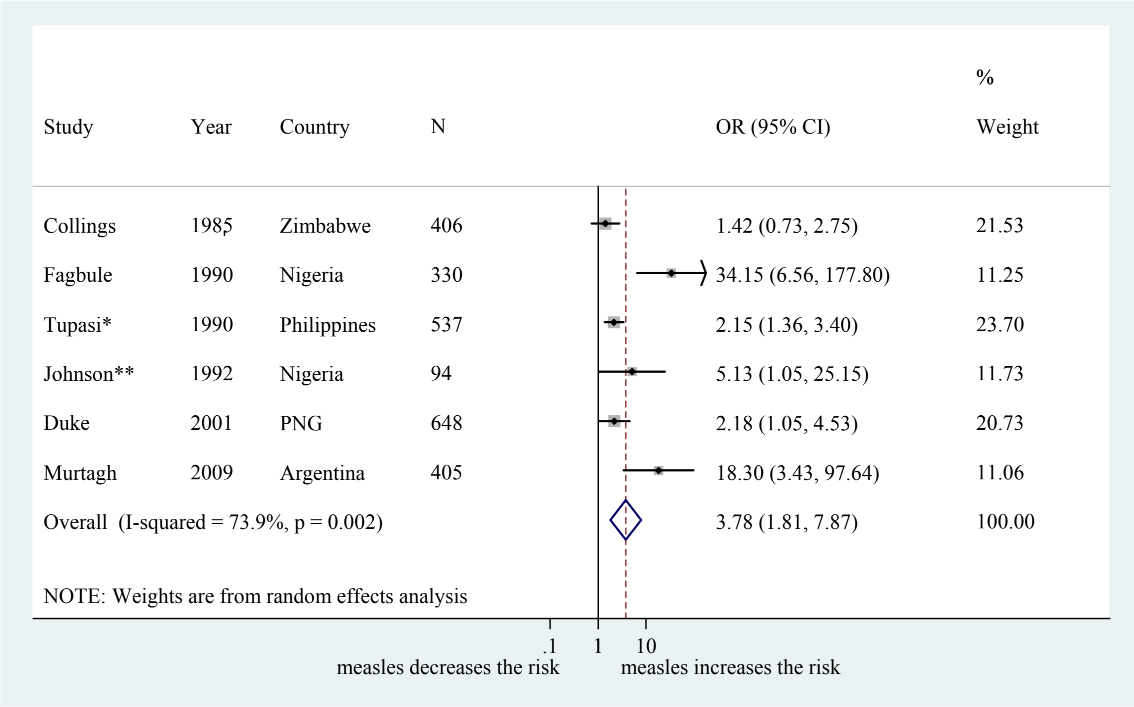

* includes Tupasi etiology 1990

** includes Johnson “Host factors...”1992

Figure 15 Association between previous ALRI and death from ALRI (5 studies; 3,517 children)

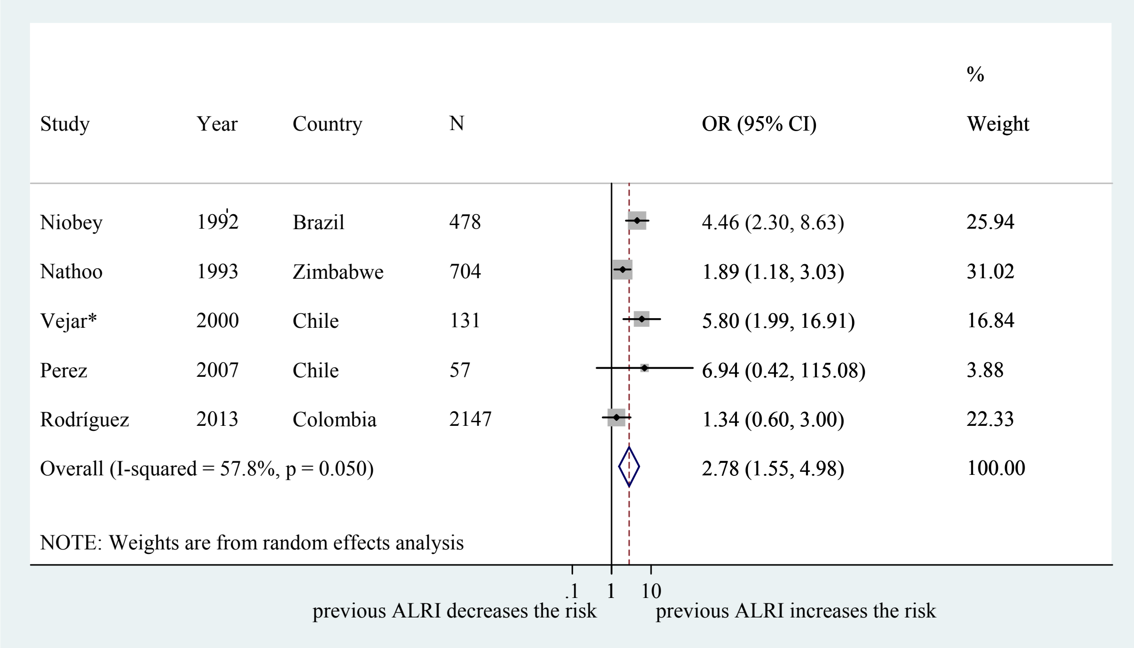

*Vejar: aOR

Figure 16 Association between severe pneumonia and death from ALRI (6 studies; 7,581 children)

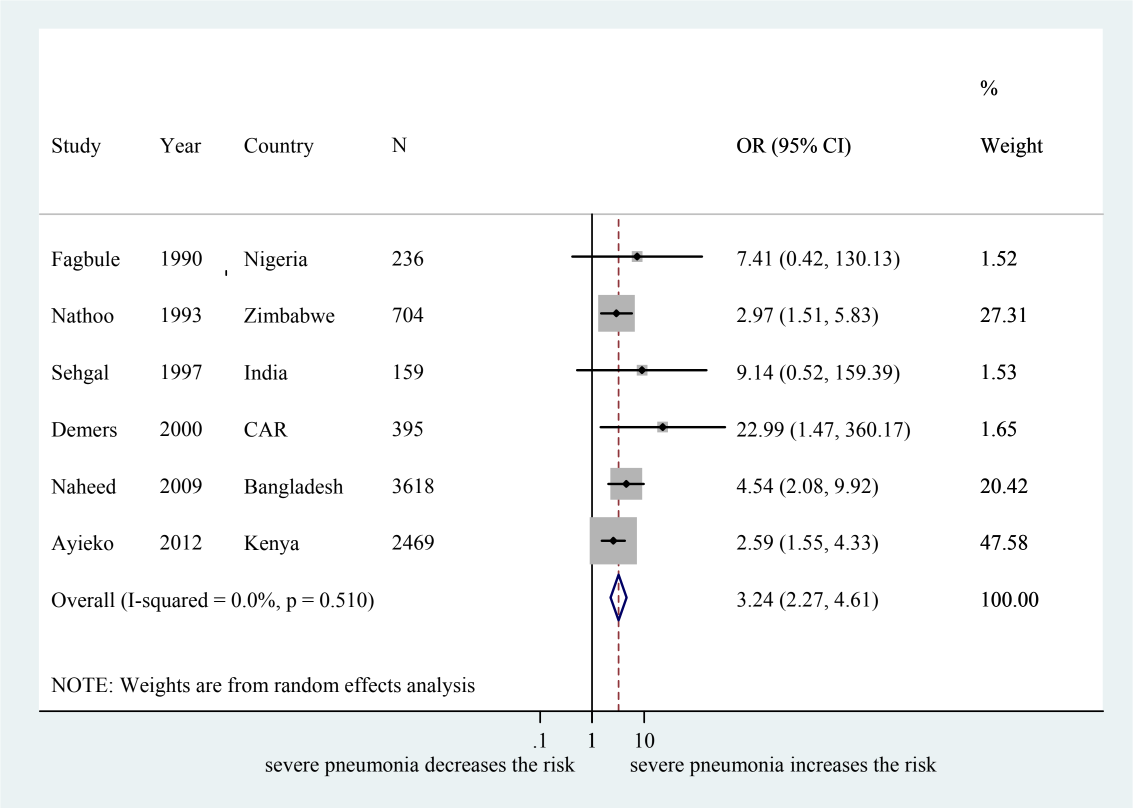

Figure 17 Association between very severe pneumonia and death from ALRI (12 studies; 7,232 children)

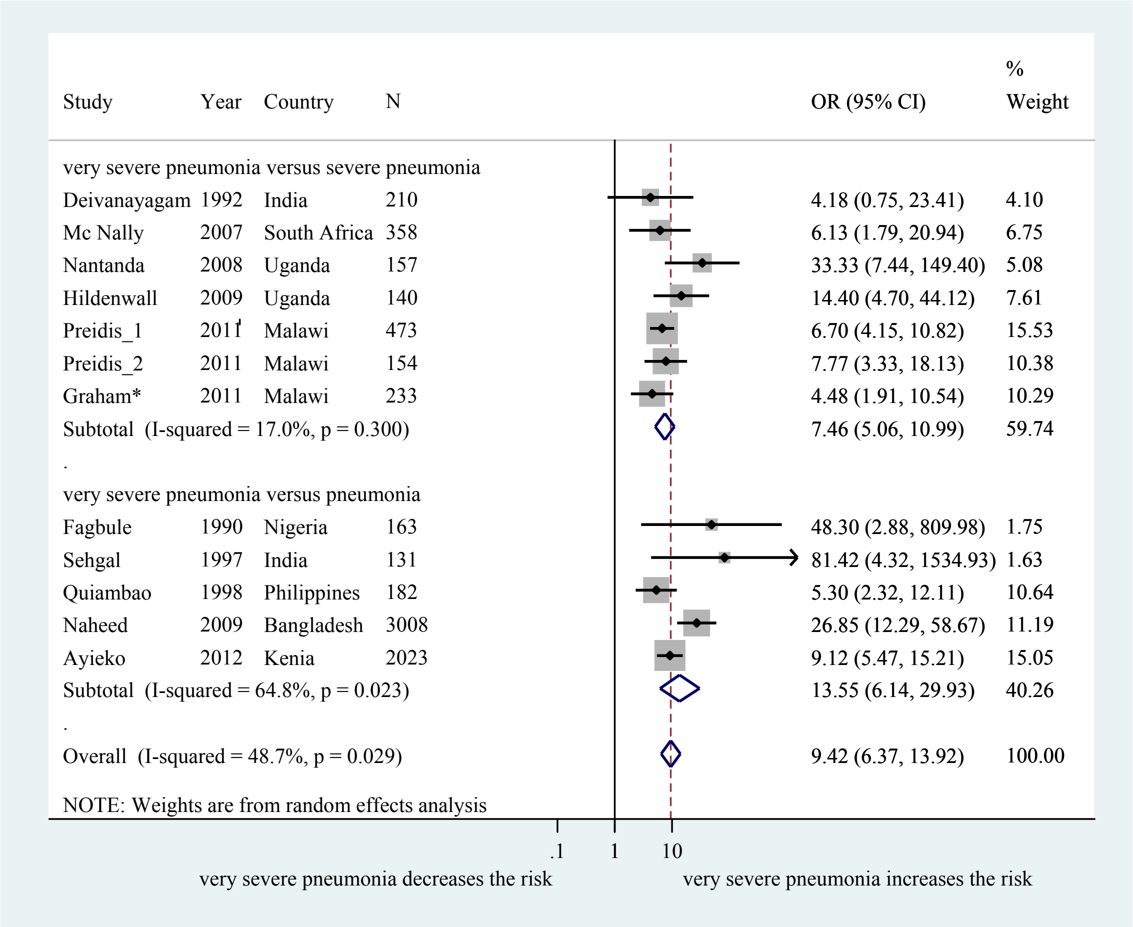

*includes Ramakhrishna 2012
Preidis_1: HIV negative
Preidis_2: HIV positive

Figure 18 Association between diagnosis of Respiratory Syncytial Virus (RSV) and death from ALRI (6 studies; 7,595 children)

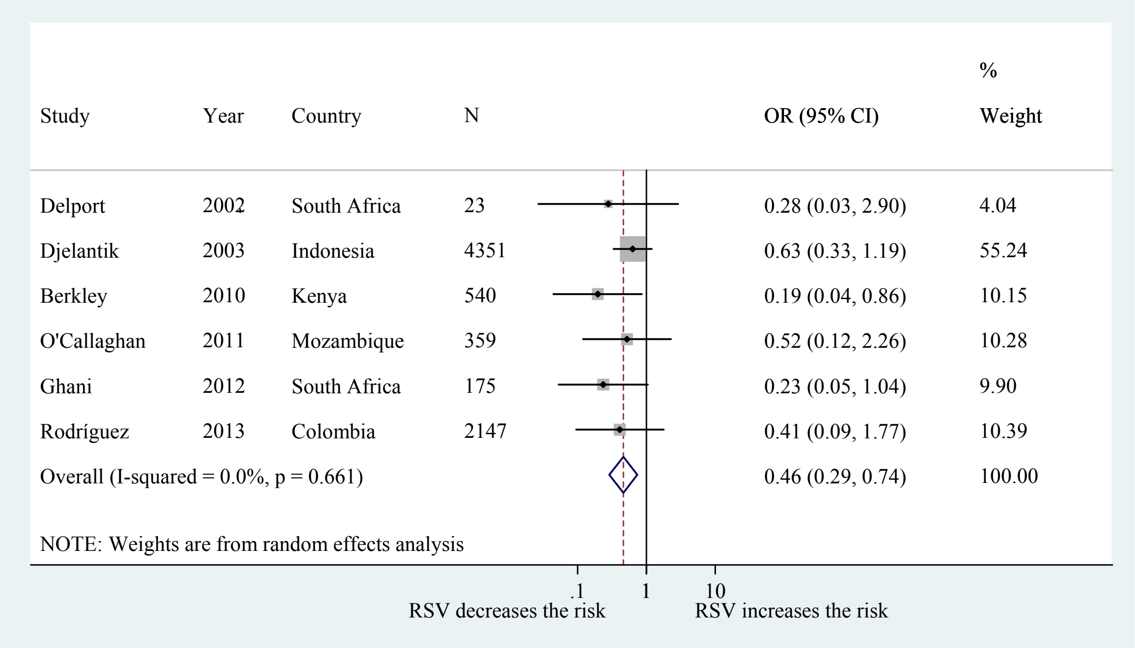

Figure 19 Association between diagnosis of Pneumocystis Carinii (PCP) and death from ALRI (5 studies; 952 children)

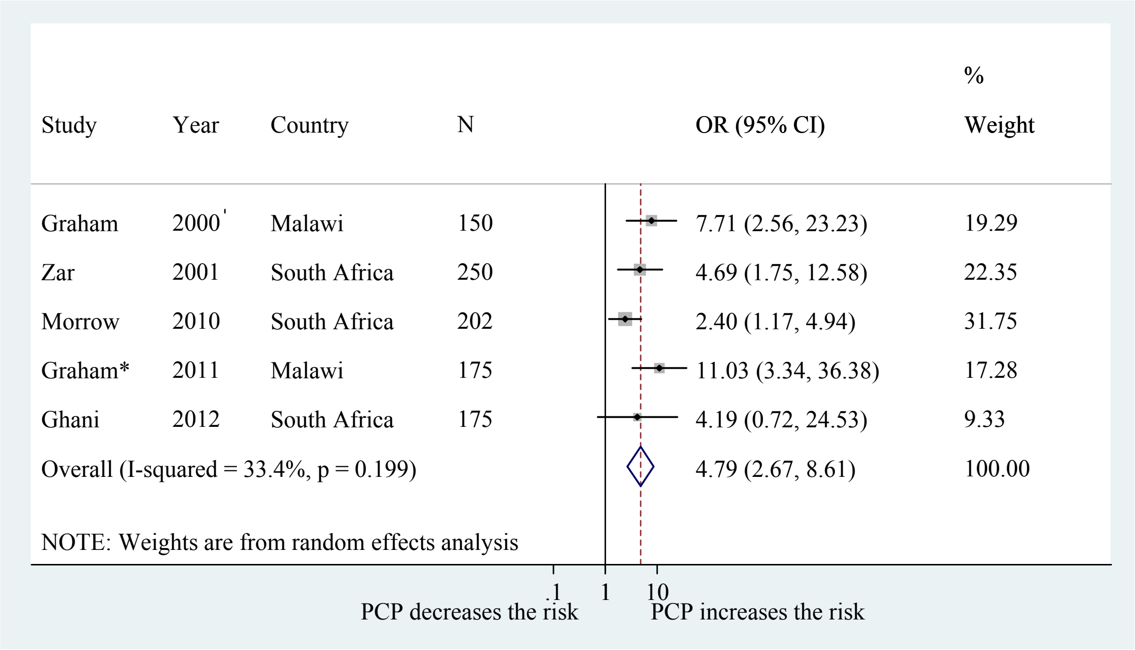

* includes Ramakrishna 2012

Figure 20 Association between mother's age and death from ALRI (4 studies; 1,225 children)

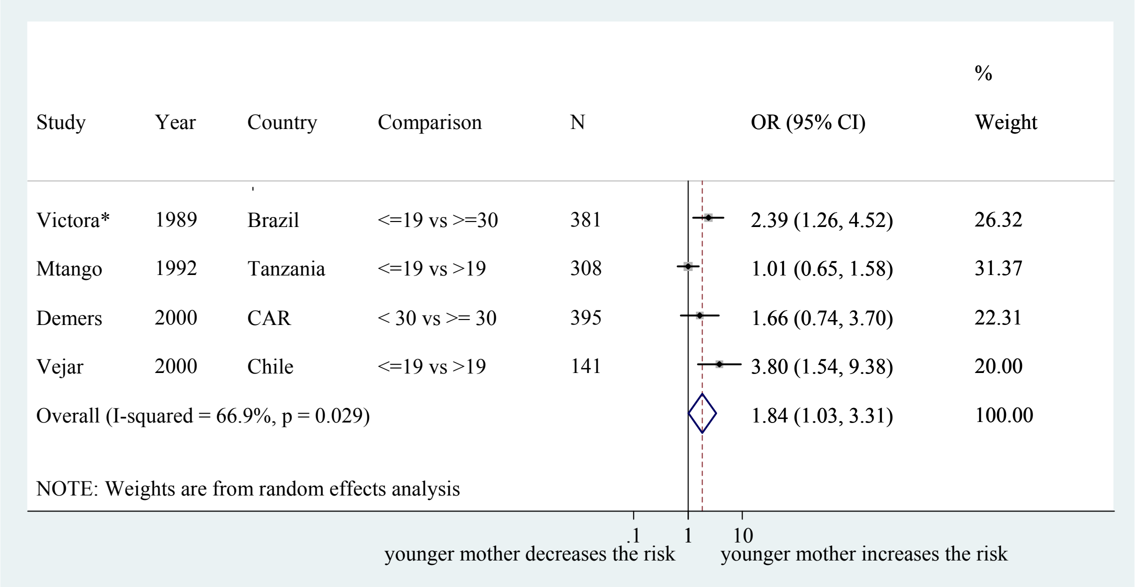

* includes Victora 1988

Other studies not included in the forest plot

- Shah 2012: OR for one year increase in mother's age: 0.99 (0.93-1.05)

Figure 21 Association between mother's low educational level and death from ALRI (14 studies; 26,130 children)

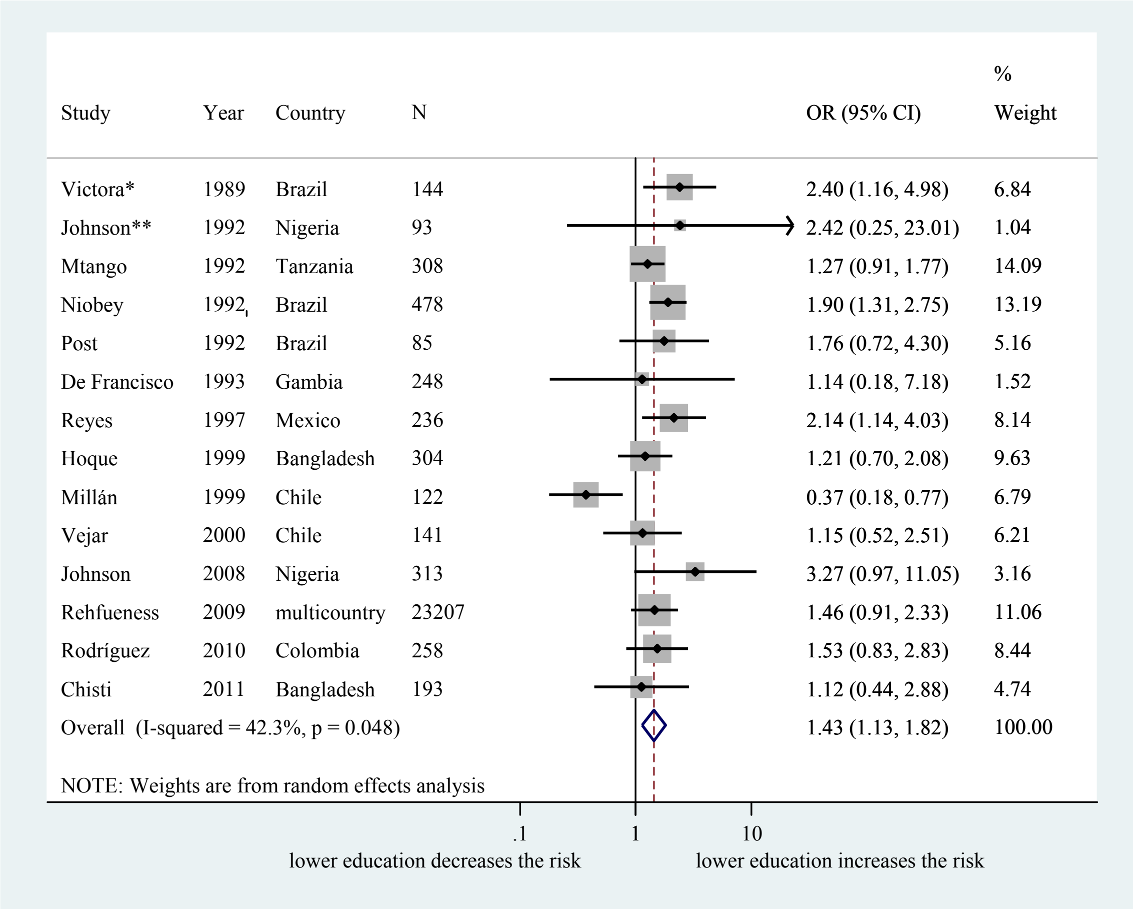

* includes Victora 1988
** includes Johnson "Host factors..." 1992

Other studies not included in the forest plot

- Arifeen 2013: any education vs none: RR:0.52 (0.32-0.86)

Figure 22 Association between mother’s paid job and death from ALRI (3 studies; 520 children)

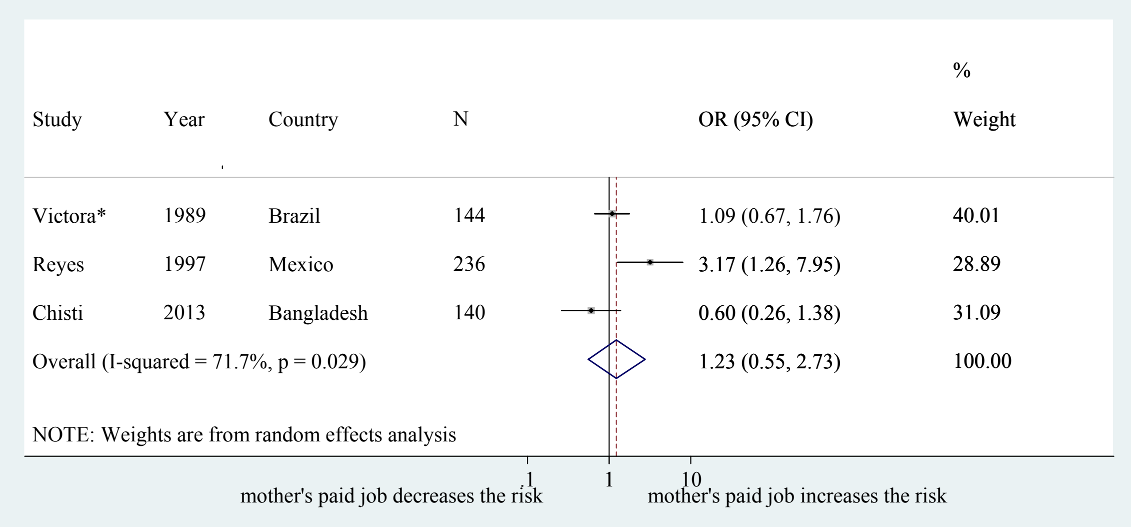

* includes Victora 1988

Other studies on maternal factors not included in the forest plot

- Mtango 1992. Maternal parity <=2 : OR=0.70 (0.49-1.0)
- Mc Nally 2007. Maternal TB: OR 4.36 (1.42-13.24)
- Victora 1989.
  - Race of the mother (ref = white):
    - black: OR 1.41(0.73-2.70);
    - mixed: OR 0.84 (0.48-1.48)
  - Cesarean delivery (vs vaginal): OR 0.72 (0.42-1.25)

- Millán 1999.
  - unwanted pregnancy: OR 0.60 (0.35-1.0)
  - diseases in pregnancy: OR 2.62 (1.41-4.85)

- Arifeen 2013: maternal height ≥150 cm vs < 145 cm RR 0.72 (0.46-1.12)

Figure 23 Association between socioeconomic status and death from ALRI (9 studies; 11 comparisons; 13,908 children)

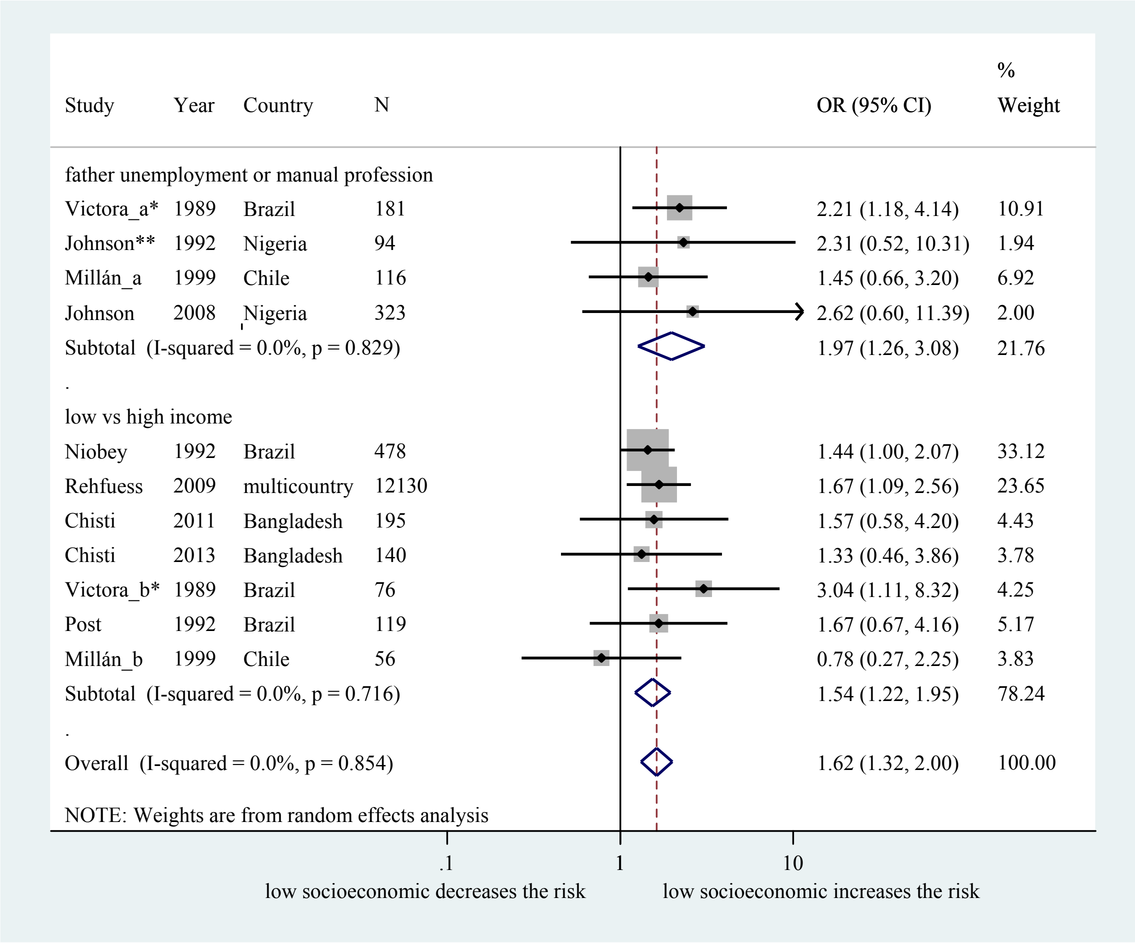

*includes Victora 1988
** includes Johnson "Host factors..." 1992

Other studies on socioeconomic factors not included in the forest plot

- Chisti 2011: Sleeping in a wooden bed with no covering: OR 1.7 (0.5-6.36)
- Rodríguez 2010: Humidity in the house OR: 2.75 (0.92-8.16)

Figure 24 Association between sanitation and water, and death from ALRI:
- lack of sewerage/latrine (3 studies; 904 children)
- poor water quality (3 studies; 1922 children)

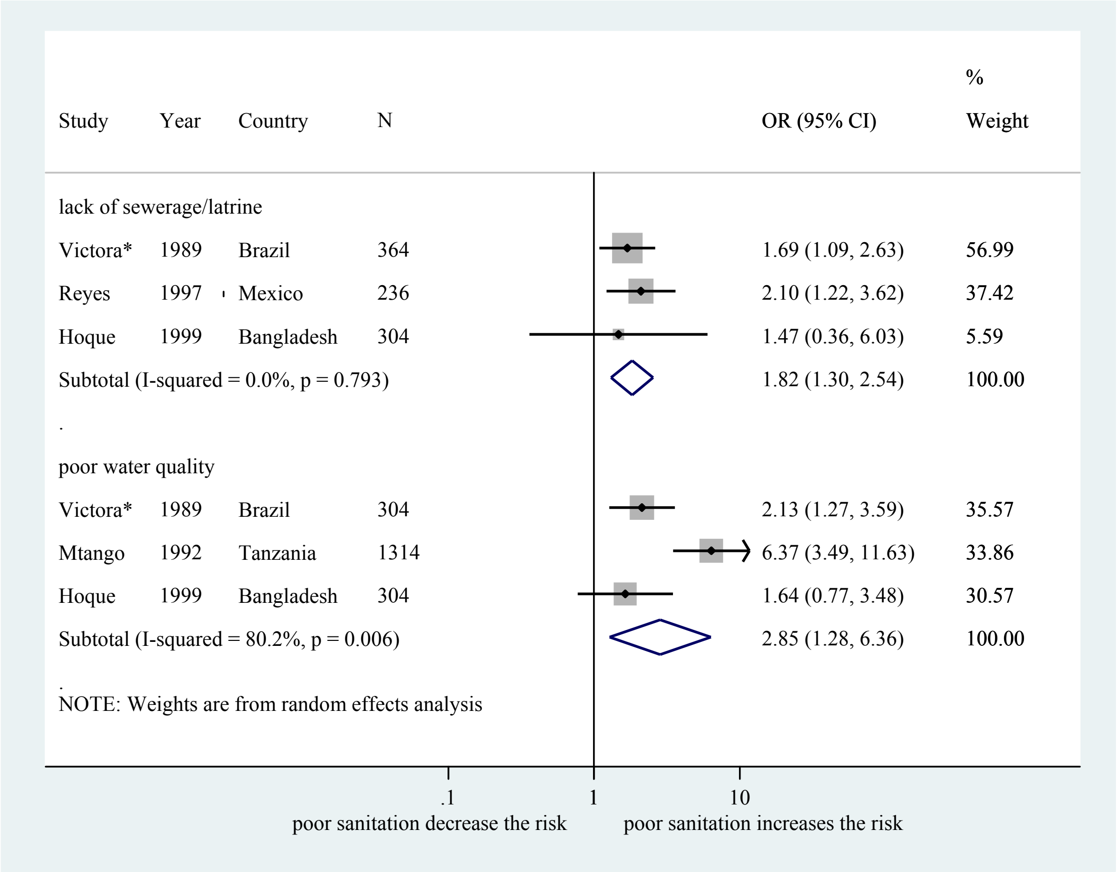

*includes Victora 1988

Other studies not included in the forest plot

- Hoque 1999.
  - Dirty latrine vs clean: OR 1.83 (0.91-3.60)
  - No agent used for washing hands : OR 1.27 (0.58-2.80)
  - Water used for washing hands (other vs tube well): OR 1.41 (0.76-2.63)

Figure 25 Association between crowding and death from ALRI (9 studies; 3,285 children)

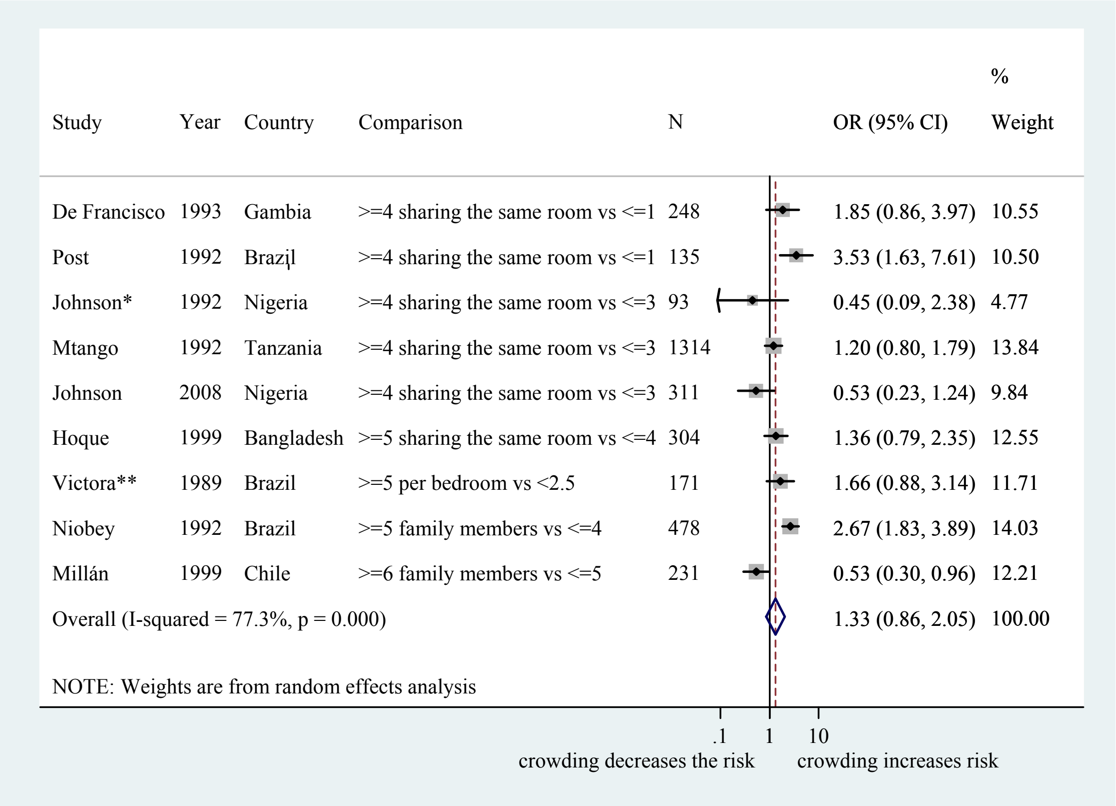

*includes Johnson "Host factors" 1992
**includes Victora 1988

Figure 26 Association between second-hand smoke and death from ALRI (8 studies; 3,044 children)

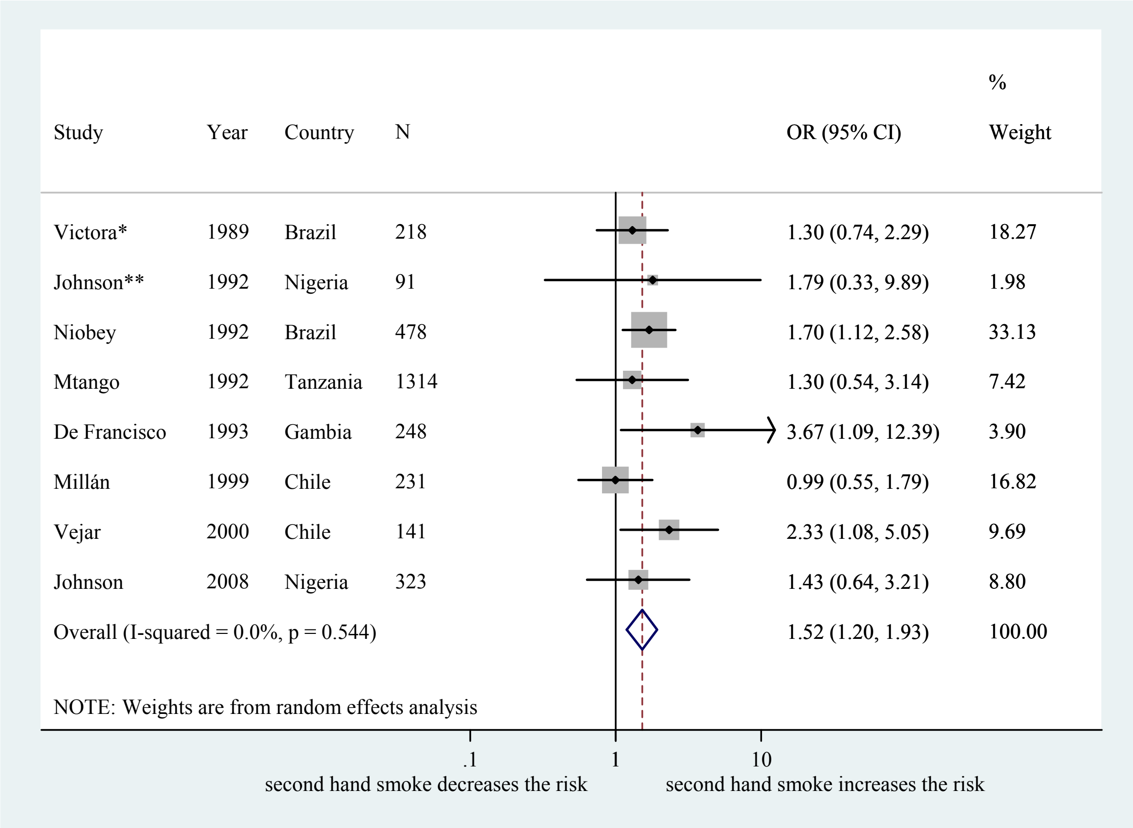

*includes Victora 1988
** includes Johnson "Host factors" 1992

Figure 27 Association between indoor pollution and death from ALRI (6 studies; 32,635 children)

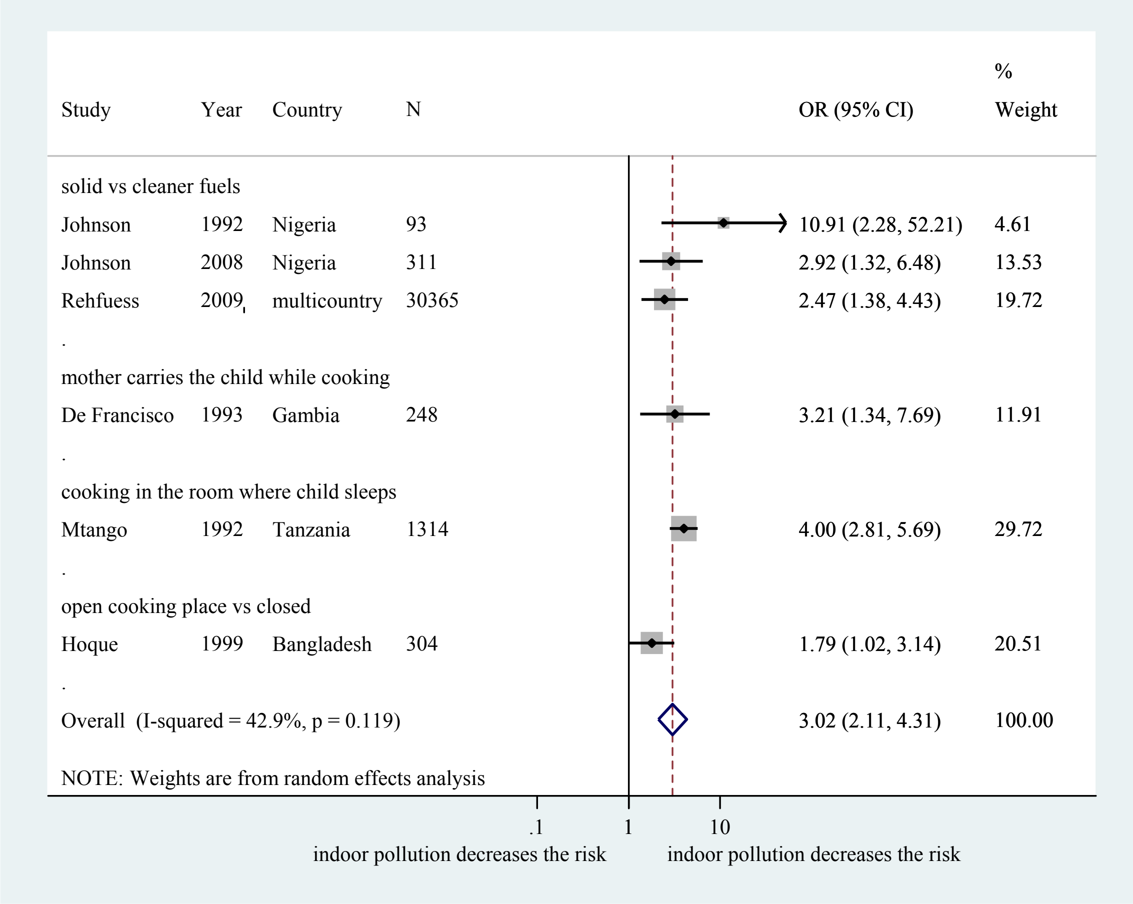

*includes Johnson “Host factors...” 1992

Figure 28 Association between seasonality (wet versus dry season) and death from ALRI (4 studies; 18,727 children)

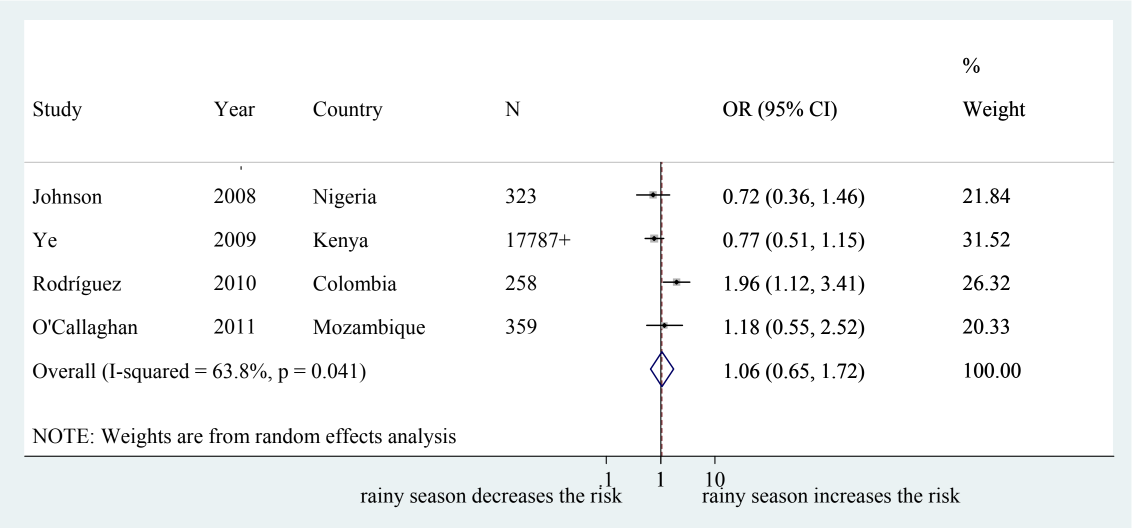

+children-year

Figure 29 Association between setting of residence (rural versus urban zone) and death from ALRI (3 studies; 78,752 children)

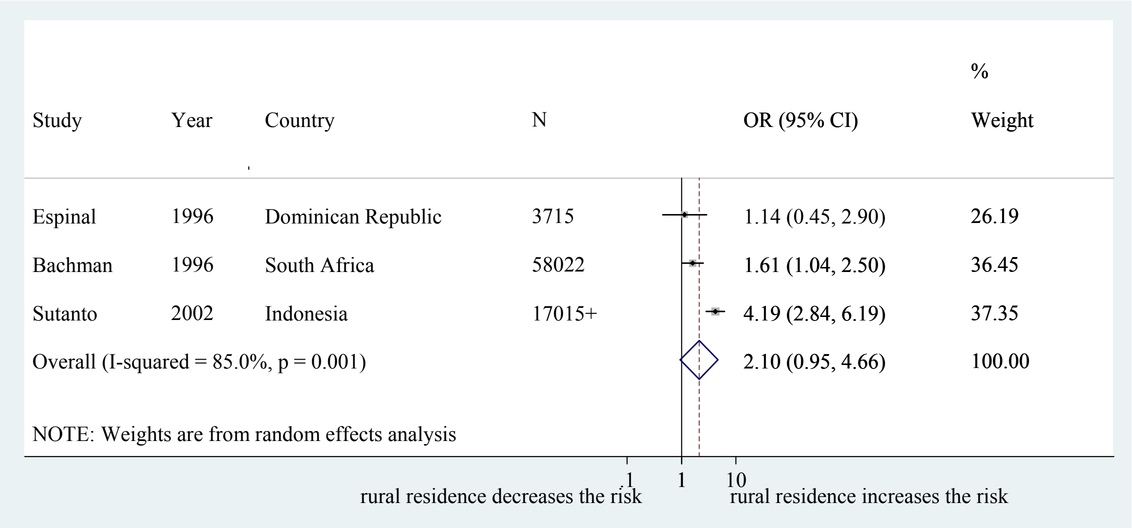

+ children-year

Figure 30 Association between immunization and death from ALRI (12 studies; 6,006 children)

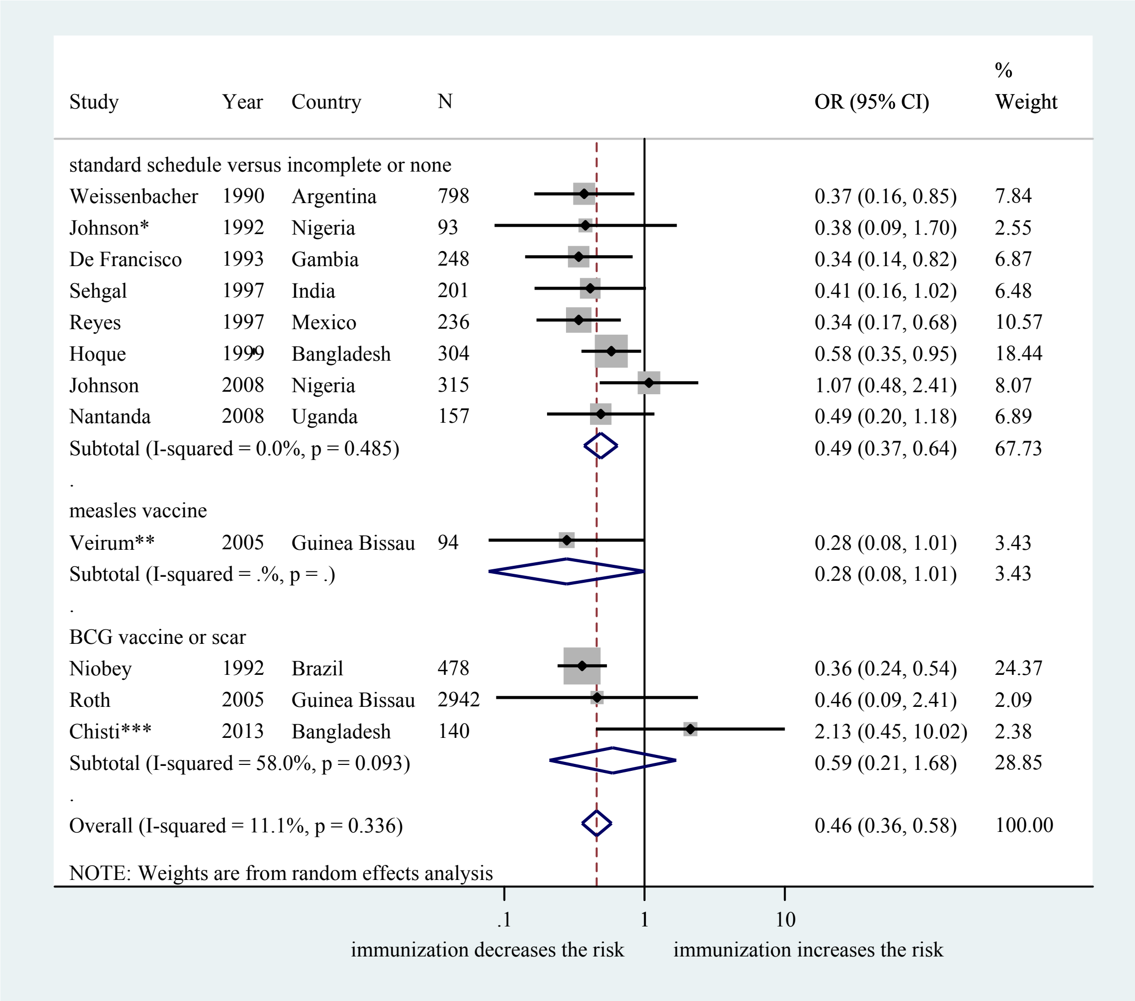

*includes Johnson "Host factors" 1992
** measles cases excluded
*** all severely malnourished children

Figure 31 Association between antenatal good practices and death from ALRI (3 studies; 4 comparisons; 646 children)

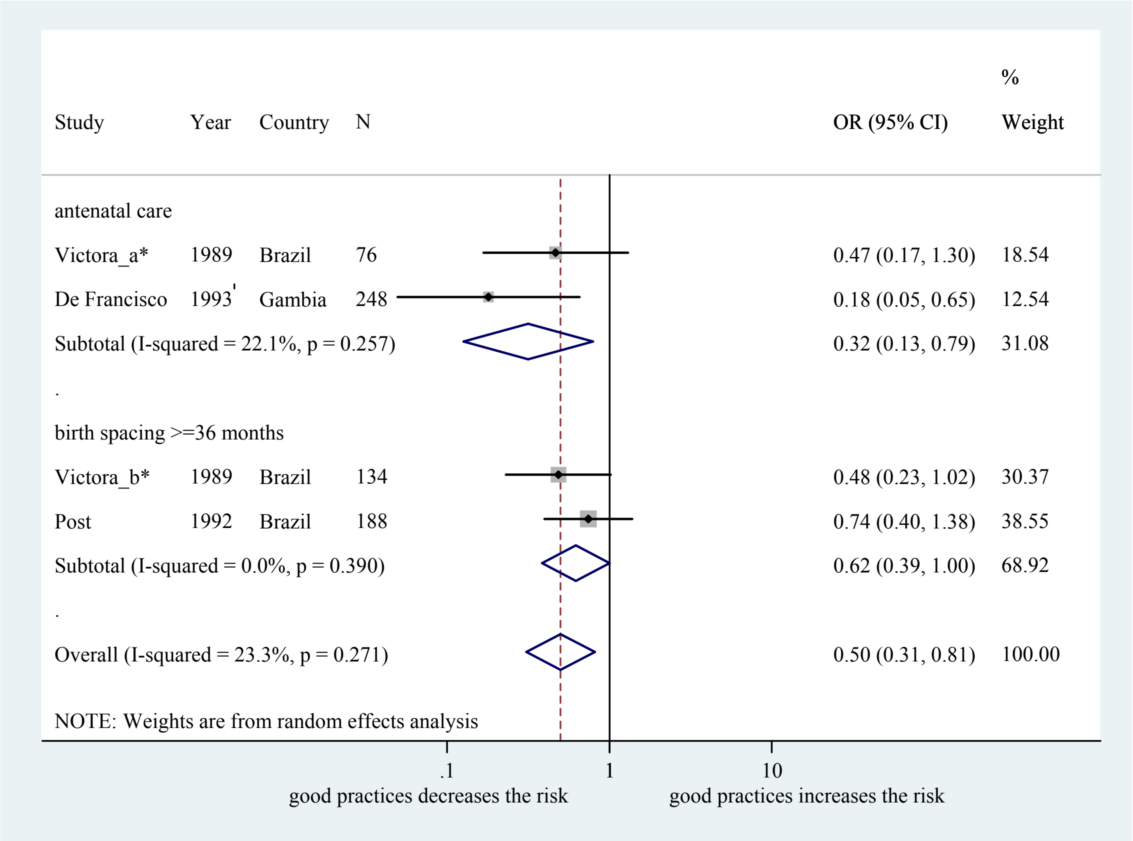

*includes Victora 1988

Other studies on preventive practices not included in the forest plot

- Mtango 1992: maternal child card present OR 0.02 (0.01- 0.04)
- De Francisco 1993: Child ever visited welfare clinic: OR 0.13 (0.05- 0.29)

Figure 32 Association between pre-hospital care and death from ALRI (3 studies; 4,824 children)

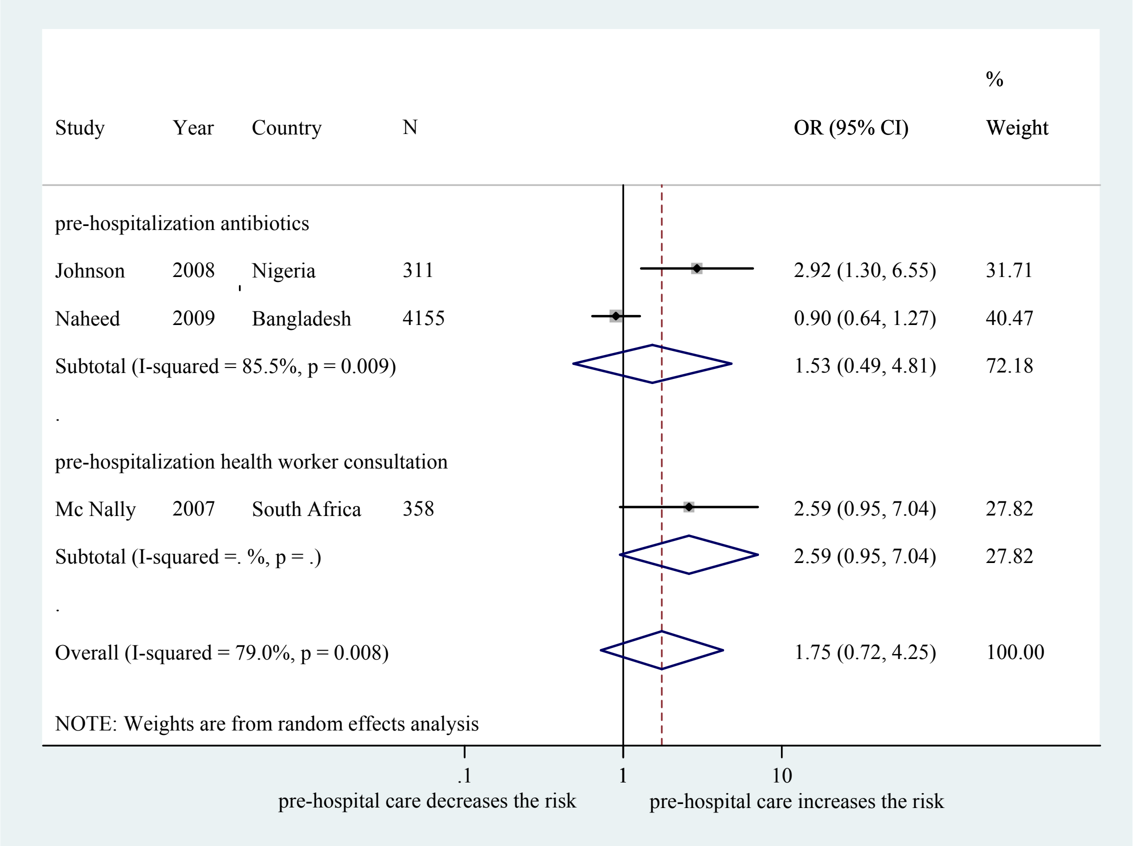

Other studies realted to health-care factors

- Chisti 2011: poor understanding of ALRI by caretakers: OR 0.60 (0.24-1.49)
- Mtango 1992: dispensary <=3km: OR 0.65 (0.60-1.19)
- Reyes 1997
    - lack of identification of ALRI by caretakers: OR 2.13 (1.15-3.94
    - late referral by caretakers: OR 20 (2.70-148.60):
    - non-institutional barriers (economical, geographical, cultural) to access to care: OR 3.12 (1.41-6.93);
    - late referral by primary care: OR 7.56 (3.78-15.13)
