# Supplementary material for: Risk Factors for Mortality from Acute Lower Respiratory Infections (ALRI) in Children under Five Years of Age in Low and Middle-Income Countries: A Systematic Review and Meta-Analysis of Observational Studies
Source: PLoS One. 2015 Jan 30;10(1):e0116380. doi: 10.1371/journal.pone.0116380 (PMC4312071; doi:10.1371/journal.pone.0116380)

S1 Funnel plots: Funnel plots of risk factors with significant Egger's test

Figure 1 Funnel plot for prematurity

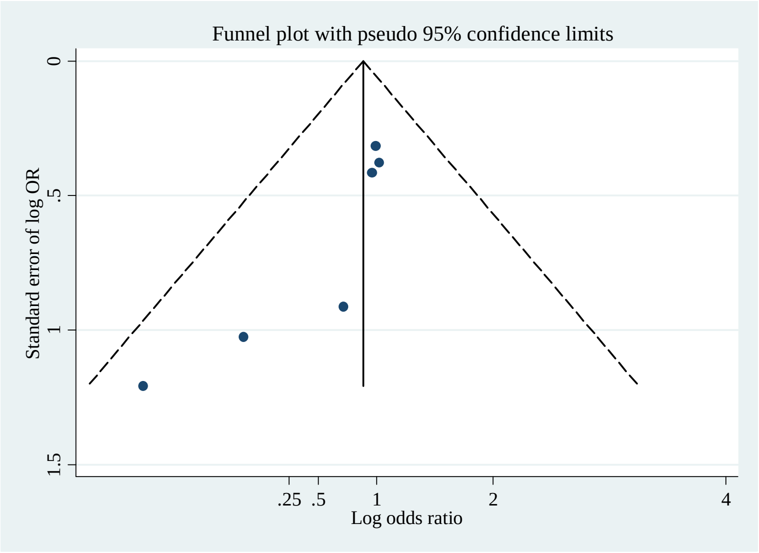

Figure 2 Funnel plot for severe malnutrition

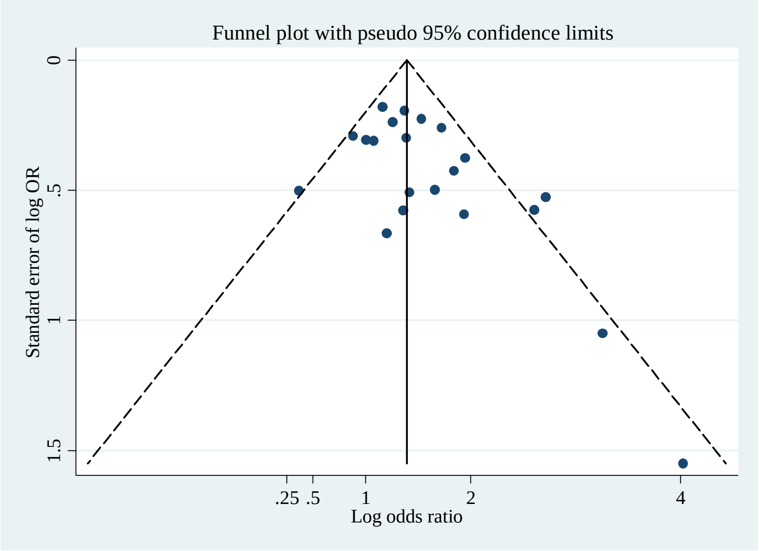

Figure 3 Funnel plot for comorbidity with chronic diseases

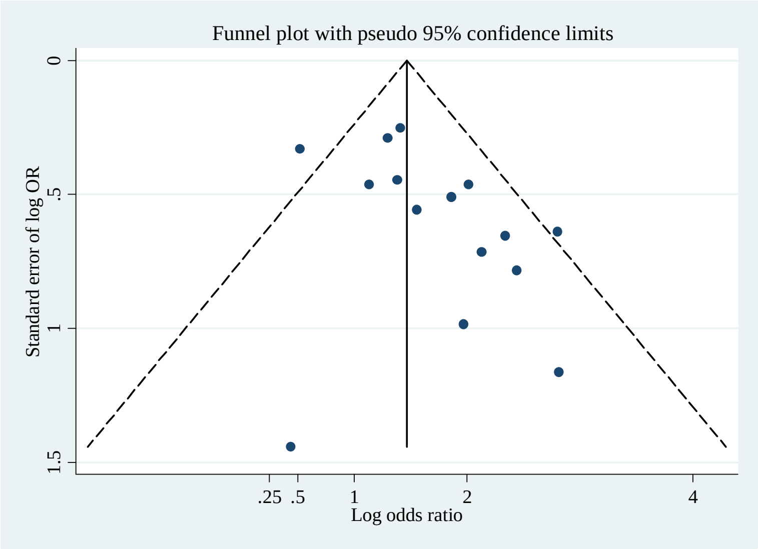

Figure 4 Funnel plot for measles

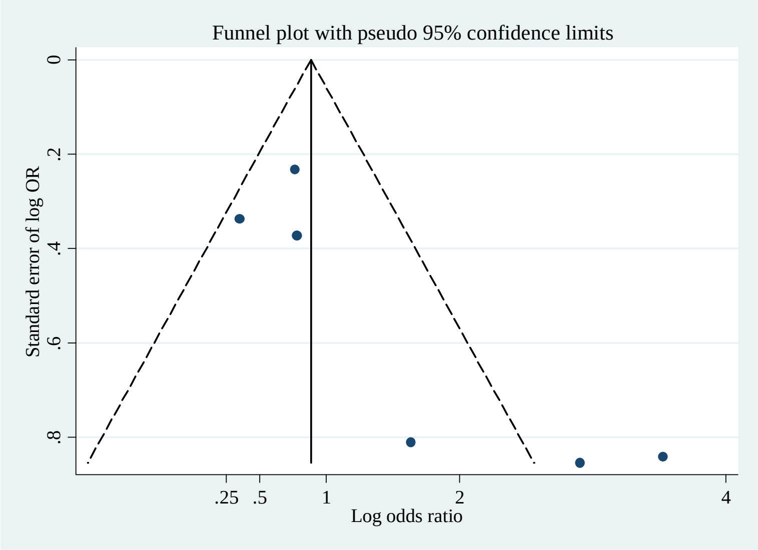

Figure 5 Funnel plot for severe pneumonia

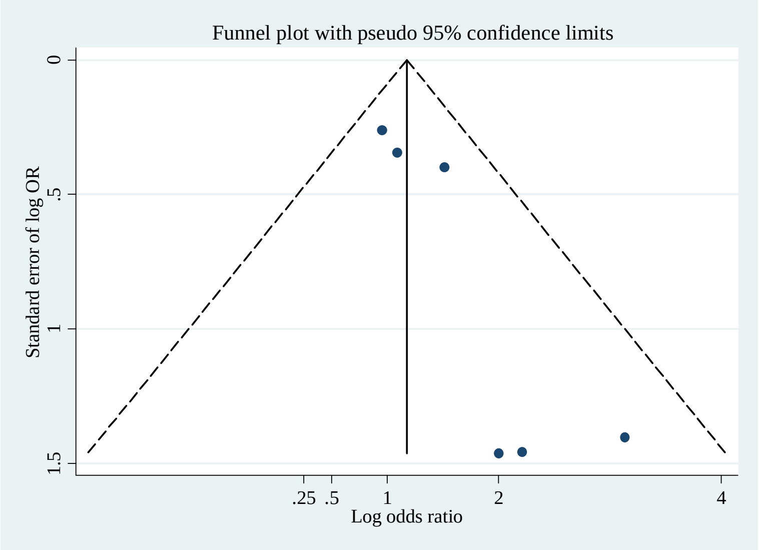

Supplement: S1 Funnel Plots — (PDF) [file pone.0116380.s003.pdf]
