# Supplementary material for: Risk Factors for Mortality from Acute Lower Respiratory Infections (ALRI) in Children under Five Years of Age in Low and Middle-Income Countries: A Systematic Review and Meta-Analysis of Observational Studies
Source: PLoS One. 2015 Jan 30;10(1):e0116380. doi: 10.1371/journal.pone.0116380 (PMC4312071; doi:10.1371/journal.pone.0116380)
Supplement: S1 Table — (PDF) [file pone.0116380.s004.pdf]

S1 Table. Characteristics of included studies

Author and year of publication	Country	Period of study	Setting (source population)	Type of study	Diagnostic criteria for ALRI	Age group (months)	Males (%)	Others characteristics of study population	ALRI mortality+	Nº of children evaluated	Analyzed risk factors
Agrawal 1995 [1]	INDIA	1993	Hospital (Urban)	CO	Clinical	2-60	57.5		11.8	127	S, A, M
Arifeen 2001 [2]	BANGLADESH	1993-1995	Community (Slums)	CO	Verbal autopsy	0-12			2.9	1677	BW, BF, MED
Ayieko 2012 [3]	KENYA	2007-2008	Nine Hospitals (Rural)	CO	Clinical WHO	6-23 (IQR)	53.2		5.9	3319	S, A, Malaria, Diarrhoea/dehydration, SEV
Bachmann 1996 [4]	SOUTH AFRICA	1992-1993	Community (Urban and rural)	CO	Death certificate	<12			0.25	58022	Rural/Urban zone
Bahl 2005 [5]	INDIA GHANA PERU	1995-1997	Community (Slums and rural)	CO	Clinical or verbal autopsy	1-6			0.58/100 children-years	9200	BF
Bahwere 2004 [6]	DRC	1987-1997	Hospital (Rural)	CO	Clinical and radiological	not stated	52.9		13.1	848	S, A, M, Dehydration
Banajeh 1997 [7]	YEMEN	1995-1996	Hospital (Rural)	CO	Clinical WHO and radiological	0-59	66.9	All severe ALRI	9.8	529	S, A, M
Banajeh 1998 [8]	YEMEN	1991-1995	Hospital (Rural)	CO	Clinical WHO and radiological	0-59	63.9	All severe ALRI	8.7	2554	A
Berkley 2010 [9]	KENYA	2007-2009	Hospital (Rural)	CO	Clinical WHO	0-144	59.0	All severe ALRI	3.2	759	RSV
Chisti 2011 [10]	BANGLADESH	2007	Hospital (Urban)	CO	Clinical WHO	0-59		All severe ALRI All ALRI and diarrhoea	12.1	198	A, M, BF, MED, SES
Chisti 2013 [11]	BANGLADESH	2011-2012	PICU (Urban)	CC	Radiological	0-59	60.7	All severe ALRI All severe acute malnutrition		140 (35 ca, 105 live co)	A, S, BF, Diarrhoea/dehydration, Mother’s employment, SES, I

Collings 1985 [12]	ZIMBABWE	1982-1983	Hospital (Urban and rural)	CO	Clinical	1-36	54.0		10.3	406	A, M, Measles
Cotes 2012 [13]	COLOMBIA	2000-2006	Three Hospitals (Urban)	CO	ICD-10 discharge codes for ALRI	0-23	53.6	ALRI only during flu season	3.5	535	Underlying diseases
De Francisco 1993 [14]	GAMBIA	not stated	Community (Rural)	CC	Verbal autopsy	0-23				248 (124 ca, 124 live co)	M,BF, MED, CW, Second-hand smoke, Indoor pollution, I, Preventive health practices
Deivanayagam 1992 [15]	INDIA	1989	Hospital (Urban)	CC	Clinical WHO and radiological	1-132				210 (70 ca, 140 live co)	A, M, Underlying diseases, SEV
Delport 2002 [16]	SOUTH AFRICA	1994-1995	PICU (Urban)	CO	Clinical and radiological	0-60		All severe ALRI	30.4	23	A, Prematurity, HIV, RSV
Demers 2000 [17]	CENTRAL AFRICAN REPUBLIC	1996-1997	Hospital (Urban)	CO	Clinical WHO	0-60	48.3		12.4	395	A, Birth order, BW, M, MAG, SEV
Djelantik 2003 [18]	INDONESIA	1999-2001	Three Hospitals (Rural)	CO	Clinical WHO	0-24		All severe ALRI	11.6	4351	S, A, M, RSV
Duke 2001 [19]	PNG	1998-1999	Hospital (Rural)	CO	Clinical WHO combined with PNG guidelines	1-59		All severe ALRI	6.5	648	M, Measles
El Kholy 2013 [20]	EGYPT	2010-2011	Hospital (Urban)	CO	Clinical	0-60	57.7	All severe RSV ALRI	5.0	240	S, A, Underlying diseases
Espinal 1996 [21]	DOMINICAN REPUBLIC	1996	Community (Urban and rural)	CO	Verbal autopsy	<12			0.7	3715	Rural/Urban zone
Fagbule 1990 [22]	NIGERIA	1985-1986	Hospital (Urban)	CO	Clinical	< 60 (9%>60)	62.4		9.7	330	A, Underlying diseases, Measles, SEV
Ferreira 2013 [23]	BRAZIL	1996-2000	Hospital (Urban)	CO	Clinical WHO and radiological	1-144	57.6		5.8	413	S, A, M, SEV
Ghani 2012 [24]	SOUTH AFRICA	2009	PICU (Urban)	CO	Clinical and laboratory proven viral infection	2-13 (IQR)	49.0	All severe viral ALRI	11.4	175	S, A, M, HIV, RSV, PcP

Graham 2000 [25]	MALAWI	1996	Hospital (Urban)	CO	Clinical WHO and radiological	2-59 (IQR)	53.3	All severe ALRI >50% HIV infected	22.0	150	A, HIV, PcP
Graham 2011 [26] (includes Ramakrishna 2012 [27])	MALAWI	2005-2006	Hospital (Urban)	CO	Clinical WHO	2-156 (12%>60)	54.1	All severe ALRI >50% HIV infected	10.1	327	A, M, HIV, PcP, SEV
Hildenwall 2009 [28]	UGANDA	2006	Hospital (Urban)	CO	Clinical WHO and radiological	2-59	47.9	All severe ALRI	12.9	140	SEV
Hoque 1999 [29]	BANGLADESH	1992-1994	Community (Rural)	CC	Verbal autopsy	1-59				304 (152 ca, 152 live co)	BF, MED, Sanitation, CW, I
Hussain 1999 [30]	BANGLADESH	1992	Community (Slums)	CO	Verbal autopsy	0-59	51.2		1.0	2351	S, A
Johnson 1992[31] (includes Johnson 'Host Factors' 1992[32])	NIGERIA	1985-1986	Hospital (Urban)	CO	Clinical	0-59	53.4		7.8	103	S, A, M, BF, MED, SES, CW, Second-hand smoke, Indoor pollution, I, Measles
Johnson 2008 [33]	NIGERIA	not stated	Hospital (Urban)	CO	Clinical and radiological Denny and Clyde defined ALRI	0-59	54.8		10.8	323	A, S, Birth order, M, MED, SES, CW, Second-hand smoke, Indoor pollution, Seasonality, I, Preconsultation
Kitchin 2011 [34]	SOUTH AFRICA	2007	Hospital (Ward+PICU) (Urban)	CO	Clinical WHO and radiological	not stated	53.0	All severe ALRI >50% HIV infected	25	132	HIV
Lehmann 1996 [35]	PNG	1979-1984	Community (Rural)	CO	Verbal autopsy	0-12	49.4		3.3	1711	BW
Lupisan 2007 [36]	PHILIPPINES	1994-2000	Hospital (Rural)	CO	Clinical WHO	2-59	58.3	All severe ALRI	2.4	1249	S, A, M
Mahdi 2000 [37]	SOUTH AFRICA	1997-1998	Hospital (Urban)	CO	Clinical WHO and/or p02<90%	2-60		All severe ALRI	7.3	1165	M, HIV
Man 1998 [38]	GAMBIA	1993-1995	Two Hospitals (Urban and rural)	CO	Clinical	0-60			7.0	2193	M

Mathur 2002 [39]	INDIA	not stated	PICU (Urban)	CO	Clinical or Radiological	<1		All newborns	31.1	103	A, Prematurity, BW
Mc Nally 2007 [40]	SOUTH AFRICA	2001-2002	Hospital (Urban)	CO	Clinical WHO	1-59	54.7	All severe ALRI >50% HIV infected	15.1	358	A, M, HIV, Underlying diseases, Diarrhoea, Preconsultation, SEV
Millán 1999 [41]	CHILE	1995	Community (Urban)	CC	ICD-9 discharge codes of ALRI	<12	53.8			231 (113 ca, 118 live co)	S, Prematurity, Birth order, BW, BF, Underlying diseases, MED, SES, CW, Second-hand smoke
Morrow 2010 [42]	SOUTH AFRICA	2006-2008	Hospital (Ward+PICU) (Urban)	CO	Clinical WHOand p02 < 90%	2.1-4.6 (IQR)	45.5	All severe ALRI >50% HIV infected	25.2	202	HIV, PcP
Mtango 1992 [43]	TANZANIA	1986-1987	Community (Rural)	CC	Verbal autopsy and/or medical record	<60	49.1			1314 (154 ca, 1160 live co)	S, A, BF, MAG, MED, Sanitation, CW, Second-hand smoke, Indoor pollution
Murtagh 2009 [44]	ARGENTINA	1998-2005	Hospital (Urban)	CO	Clinical and laboratory proven viral infection	<72	61.5	All Adenovirus ALRI	12.8	405	Measles
Naheed 2009 [45]	BANGLADESH	2004-2007	Seven Hospitals	CO	Clinical WHO	2-59	64.0		3.6	4155	A, M, Preconsultation, SEV
Nantanda 2008 [46]	UGANDA	2005-2006	Hospital (Urban)	CO	Clinical WHO	2-59		All severe ALRI	15.3	157	A, M, HIV, I, SEV
Nascimento-Carvalho 2002 [47]	BRAZIL	1997-1999	Two hospitals (Urban)	CO	Clinical WHO or radiological	0-59	54.1		1.2	1762	A, M, Underlying diseases
Nathoo 1993 [48]	ZIMBABWE	1989-1990	Hospital (Urban and rural)	CO	Clinical WHO	1-60	55.7		14.8	704	A, BW, M, HIV, Diarrhoea, Previous ALRI, SEV
Niobey 1992 [49]	BRAZIL	1986-1987	Community (Urban)	CC	ICD-9 death certificate for ALRI	1-12				478 (255 ca, 223 live co)	Birth order, BW, BF, Previous ALRI, MED, SES, CW, Second-hand smoke, I
O'Callaghan 2011 [50]	MOZAMBIQUE	2006-2007	Hospital (Rural)	CO	Clinical WHO and laboratory proven viral infection	<60	63.0	All severe viral ALRI	9.2	359	S, A, HIV, Malaria, Seasonality, RSV

Onyango 1993 [51]	KENYA	1989	Hospital (Urban)	CO	Clinical	0-36			10	256	A
Pérez 2007 [52]	CHILE	2004	Hospital (Urban)	CO	Laboratory proven viral infection	1-40 (IQR)	68.4	All Adenovirus ALRI	8.8	57	Underlying diseases, Previous ALRI
Post 1992 [53]	BRAZIL	1986-1987	Hospitals (Urban)	CC	Hospital records	<12				298 (152 ca 146 live co)	Prematurity, BW, M, BF, MED, SES, CW, Preventive health practices
Preidis 2011 [54]	MALAWI	2007-2008	Hospital (Urban)	CO	Clinical WHO	11.1-44.6 (IQR)	51.1	All severe ALRI All HIV infected or exposed	16.4	627	SEV
Quiambao 1998 [55]	PHILIPPINES	not stated	Hospital (Urban)	CO	Clinical WHO and/or radiological	4-59		Only measles-associated ALRI	17	182	SEV
Quiambao 2009 [56]	PHILIPPINES	1994-2000	Hospital (Rural)	CO	Clinical and/or radiological	0-2	60.0		14.9	301	A
Ramachandran 2012 [57]	INDIA	2006-2008	Hospital (Urban)	CC cohort nested	Clinical WHO and/or radiological	1-59	58.0		8.2	Cohort: 4375 CC: 1071 (357 ca, 714 live co)	S, A, M, Underlying diseases
Rehfuess 2009 [58]	SUBSAHARAN AFRICA (16 Countries)	2003	Community (Urban and rural)	CO	Verbal autopsy	0-59			0.7% live births under-5 mortality	30365	MED, Indoor pollution
Reyes 1997 [59]	MEXICO	1992-1993	Community (Urban and rural)	CC	Death certificate and verbal autopsy	<12				236 (118 ca, 118 live co)	Prematurity, Birth order, BF, MED, Mother's employment, Sanitation, I, Health care factors
Rodríguez 2010 [60]	COLOMBIA	2007-2008	Four Hospitals (Ward+PICU) (Urban)	CC	ICD-10 death certificate for ALRI and clinical records	<60				258 (79 ca, 179 live co)	A, M, MED, Seasonality, SES, Health care factors
Rodríguez 2013 [61]	COLOMBIA	2009-2011	Hospital (Urban)	CO	Clinical record and laboratory proven RSV infection	<36		All RSV ALRI	1.1	2147	A, Prematurity, Underlying diseases, Previous ALRI, RSV
Roth 2005 [62]	GUINEA - BISSAU	1996-1999	Community (Urban)	CO	Verbal autopsy	3-60	50.9		2.3	2942	I

Sehgal 1997 [63]	INDIA	1993-1994	Hospital (Urban)	CO	Clinical WHO and/or radiological	0-60	58.2		10.4	201	S, A, M, Underlying diseases, Diarrhoea, I, SEV
Shah 2012 [64]	ERITREA	2006	PICU (Urban)	CO	Clinical and/or radiological	<1		All severe ALRI	13.7	305	S, A, BW, Gestational age, MAG
Shann 1989 [65]	PNG	1979-1982	Three Hospitals (Urban and rural)	CO	Clinical	not given		All severe ALRI	14.7	748	A, M
Sigauque 2009 [66]	MOZAMBIQUE	2004-2006	Hospital (Rural)	CO	Clinical WHO and radiological	0-23	58.1	All severe ALRI	11.1	685	S, A, M, HIV, Malaria
Smyth 1998 [67] (includes Smyth 1997 [68])	ZAMBIA	1994-1995	Hospital (Rural)	CO	Clinical WHO	1-59	51.3	All severe ALRI	14.6	158	A, M, HIV, Malaria
Sutanto 2002 [69]	INDONESIA	1998-1999	Community (Rural)	CO	Clinical WHO or verbal autopsy	<24			1.9/100 children-year	9210	A, Rural/Urban zone
Tupasi 1988[70]	PHILIPPINES	1981-1983	Hospital (Urban)	CO	Clinical and radiological	<60			4.7	729	M, SEV
Tupasi 1990 [71] (includes Tupasi 'Etiology' 1990 [72])	PHILIPPINES	1984-1986	Hospital (Urban)	CO	Clinical WHO	0-60	54		16.6	537	S, M, Measles
Uriyo 2006 [73]	TANZANIA	2003	Hospital (setting not given)	CO	Clinical WHO	2-60	62.5	50% HIV positive	12.5	72	A, HIV
Veirum 2005 [74]	GUINEA-BISSAU	1990-1996	Hospital (Urban and rural)	CO	Clinical	6-17			10.6	94	I
Vejar 2000 [75]	CHILE	1990-1994	Community (Urban)	CC	Autopsy	1-59				141 (53 ca, 88 live co)	BW, M, BF, Underlying diseases, Previous ALRI, MAG, MED, Second-hand smoke
Victora 1987 [76]	BRAZIL	1982-1984	Community (Urban)	CO	Hospital record, death certificate, autopsy, verbal autopsy	<12			0.45/100 children-year	4931	BW

Victora 1989 [77] (includes Victora 1988 [78])	BRAZIL	1984-1985	Community (Urban)	CC	Hospital record, ICD-10 death certificate, autopsy, verbal autopsy	0-12	52.9			381(127 ca, 254 live co)	S, Birth order, BW, M, BF, MAG, MED, Mother’s employment, SES, Sanitation, CW, Second-hand smoke, Preventive health practices
Weissenbacher 1990 [79]	ARGENTINA	1984-1987	Three Hospitals (Urban)	CO	Clinical	0-60	55.8		3.8	805	S, A, M, I
Ye 2009 [80]	KENYA	2003-2005	Community (Urban)	CO	Verbal autopsy	<60			2.0/100 children-year	17787	A, Seasonality
Yoon 1997 [81] (includes Yoon 1996 [82])	PHILIPPINES	1988-1991	Community (Urban and rural)	CO	Verbal autopsy	<24		ALRI or ALRI and diarrhoea	-	9942	M, BF
Zar 2001 [83]	SOUTH AFRICA	1998	Four Hospitals (Ward+PICU) (Urban)	CO	Clinical WHO	3-16 (IQR)	57.2	60% HIV positive	15.6	250	HIV, PcP

+ Case fatality rate (%) in hospital studies; mortality (% children per year or % live births) in cohort community studies.

Abbreviations: A=age; BF=breastfeeding; BW= birth weight; CW=crowding; DRC= Democratic Republic of Congo; I= immunization; IQR= Interquartile Range; M=malnutrition; MAG=maternal age; MED=maternal education; PcP= Pneumocystis carinii (jirovecii); PICU=Pediatric Intensive Care Unit; PNG= Papua New Guinea; S=sex; SES= socioeconomic status; SEV= severity; Underlying diseases=chronic diseases including congenital heart diseases; WHO= World Health Organisation.
