# Supplementary material for: Risk Factors for Mortality from Acute Lower Respiratory Infections (ALRI) in Children under Five Years of Age in Low and Middle-Income Countries: A Systematic Review and Meta-Analysis of Observational Studies
Source: PLoS One. 2015 Jan 30;10(1):e0116380. doi: 10.1371/journal.pone.0116380 (PMC4312071; doi:10.1371/journal.pone.0116380)
Supplement: S2 Table — (PDF) [file pone.0116380.s005.pdf]

Table S2: appraisal of the risk of bias of the included studies

AUTHOR	1. Study participation	2. Study Attrition	3.Risk factors measurement	4. Outcome measurement	5. Study confounding	6. Statistical analysis	Overall risk of bias
Agrawal 1995							
Arifeen 2001							HIGH
Ayeko 2012							
Bachmann 1996							HIGH
Bahl 2005							
Bahwere 2004							HIGH
Banajee 1997							
Banajee 1998							HIGH
Berkley 2010							
Chisti 2011							
Chisti 2013							
Collings 1985							HIGH
Cotes 2011							HIGH
De Francisco 1993							HIGH
Deivanayagam 1992							HIGH
Delport 2002							
Demers 2000							
Djelantik 2003							HIGH
Duke 2001							
El Kohli 2013							
Espinal 1996							HIGH
Fagbule 1990							HIGH
Ferreira 2013							
Ghani 2012							
Graham 2000							
Graham 2011*							
Hildenwall 2009							
Hoque 1999							
Hussain 1999							HIGH
Johnson 1992**							HIGH
Johnson 2008							
Kitchin 2011							HIGH
Lehman 1996							HIGH
Lupisan 2007							
Madhi 2000							
Man 1998							HIGH
Mathur 2002							HIGH
McNally 2007							
Millán 1999							HIGH
Morrow 2010							
Mtango 1992							HIGH
Murtagh 2009							HIGH
Naheed 2009							HIGH
Nantanda 2008							
Nascimento-Carvalho 2002							
Nathoo 1993							
Niobey 1992							HIGH
O'Callaghan-Gordo 2011							
Onyango 1993							HIGH
Perez 2007							HIGH
Preidis 2011							
Post 1992							HIGH

Quiambao 1998							HIGH
Quiambao 2009							
Ramachandra 2012							
Rehfuess 2009							HIGH
Reyes 1997							HIGH
Rodríguez 2010							HIGH
Rodríguez 2013							
Roth 2005							
Shah 2012							
Sehgal 1997							
Shann 1989							HIGH
Sigauque 2009							
Smyth 1998***							HIGH
Sutanto 2002							
Tupasi 1988							HIGH
Tupasi 1990****							
Uriyo 2006							HIGH
Veirum 2004							HIGH
Vejar 2000							HIGH
Victora 1987							
Victora 1988*****							
Weissenbacher 1990							HIGH
Ye 2009							
Yoon 1997******							
Zar 2001							

* includes Ramakrishna 2012
** includes Johnson “Host factors” 1992
*** includes Smyth 1997
**** includes Tupasi “Etiology” 1990
***** includes Victora 1988
****** includes Yoon 1996

	**Rating of the risk of bias in the domain**
	Low
	Moderate
	High
	Unknown
