# Supplementary material for: Risk Factors for Mortality from Acute Lower Respiratory Infections (ALRI) in Children under Five Years of Age in Low and Middle-Income Countries: A Systematic Review and Meta-Analysis of Observational Studies
Source: PLoS One. 2015 Jan 30;10(1):e0116380. doi: 10.1371/journal.pone.0116380 (PMC4312071; doi:10.1371/journal.pone.0116380)
Supplement: S3 Table — (PDF) [file pone.0116380.s006.pdf]

S3 Table. Subgroup analysis and random effect metaregression analysis for ALRI definition and HIV setting

		ALRI DEFINITION						
		WHO DEFINITION			OTHER DEFINITIONS			
**Risk factors**	**Comparisons**	**Nº of studies**	**Pooled OR**	I^2 (%)	**Nº of studies**	**Pooled OR**	I^2 (%)	p from meta-regression
Age	Age < 1y vs > 1y	9	2.49 (1.78 - 3.49)	50.6	18	1.92 (1.33 - 2.77)	81.5	0.295
Female sex	Female vs male	4	1.22 (0.95 - 1.58)	51.9	19	1.14 (1.00 - 1.30)	3.6	0.782
Low birth weight	<2500g vs >2500	2	1.95 (0.96 - 3.97)	51.7	7	3.10 (2.20 - 4.36)	30.5	0.195
Malnutrition	Severe malnutrition vs non-malnourished	5	5.06 (3.31 - 7.75)	53.3	16	3.99 (3.15 - 5.06)	38.1	0.205
	Moderate malnutrition vs non-malnourished	5	1.62 (1.01 - 2.59)	56.1	13	2.89 (2.14 - 3.90)	63.0	0.081
HIV/AIDS	Yes vs No	7	3.85 (2.82 - 5.26)	0.0	7	5.93 (4.21 - 8.36)	0.0	0.068
Diarrhoea/dehydration	Yes vs No	3	1.89 (1.44 - 2.47)	0.0	3	4.56 (2.67 - 7.78)	26.9	0.001
Respiratory Syncitial Virus	Yes vs No	2	0.43 (0.14 - 1.26)	51.0	4	0.36 (0.16 - 0.80)	0.0	0.448
Severity of pneumonia as for WHO clincal definition	Severe pneumonia vs pneumonia	4	3.21 (2.15 - 4.78)	12.5	2	8.23 (1.09 - 62.30)	0.0	0.359
	Very severe pneumonia vs severe pneumonia or pneumonia	7	9.43 (5.91 - 15.04)	59.8	5	10.06 (4.19 - 24.16)	25.7	0.966

		HIV SETTING						
		LOW HIV SETTING			HIGH HIV SETTING			
		**Nº of studies**	**Pooled OR**	I^2 (%)	**Nº of studies**	**Pooled OR**	I^2 (%)	p from meta-regression
Age	Age < 1y vs > 1y	11	2.47 (1.40 - 4.34)	89.4	11	2.85 (1.76 - 4.62)	81.4	0.679
Female sex	Female vs male	13	1.08 (0.95 - 1.22)	0.0	5	1.22 (1.00 - 1.49)	0.0	0.296
Low birth weight	<2500g vs >2500	4	3.09 (2.09 - 4.56)	0.0	2	1.95 (0.96 - 3.97)	51.7	0.183
Malnutrition	Severe malnutrition vs non-malnourished	11	4.16 (3.46 - 5.00)	0.0	5	3.91 (2.21 - 6.92)	73.8	0.535
	Moderate malnutrition vs non-malnourished	5	2.62 (1.69 - 4.07)	65.8	5	1.53 (0.97 - 2.39)	59.6	0.097
Diarrhoea/dehydration	Yes vs No	2	5.77 (2.35 - 14.17)	40.2	4	2.24 (1.53 - 3.26)	40.2	0.037

Seasonality	Wet vs dry season	2	1.22 (0.46 - 3.23)	78.9	2	0.84 (0.59 - 1.20)	0.0	0.624
Respiratory Syncitial Virus	Yes vs No	3	0.56 (0.32 - 0.99)	0.0	3	0.29 (0.12 - 0.68)	0.0	0.200
Severity of pneumonia as for WHO clincal definition	Severe pneumonia vs pneumonia	3	3.14 (2.06 - 4.80)	0.0	2	5.25 (0.87 - 31.81)	50.2	0.882
	Very severe pneumonia vs severe pneumonia or pneumonia	4	12.43 (5.43 - 28.48)	70.9	6	7.79 (5.09 - 11.92)	26.7	0.379
