# Supplementary material for: Risk Factors for Mortality from Acute Lower Respiratory Infections (ALRI) in Children under Five Years of Age in Low and Middle-Income Countries: A Systematic Review and Meta-Analysis of Observational Studies
Source: PLoS One. 2015 Jan 30;10(1):e0116380. doi: 10.1371/journal.pone.0116380 (PMC4312071; doi:10.1371/journal.pone.0116380)
Supplement: S4 Table — (PDF) [file pone.0116380.s007.pdf]

S4 Table. Sensitivity analyses and results of Egger's test

Category of risk factor	Risk factors	Comparisons	Nº. of studies included in the meta-analysis	Nº of studies with adjusted OR available	Pooled OR after substituting the adjusted OR for the crude OR	Nº. of studies of good quality	Pooled OR when including in the meta-analysis only studies of good quality	Egger regression test: p-value
Child factors	Age	Age <1y vs >1	28	3	2.49 (1.79 - 3.48)	16	2.65 (1.67- 4.20)	0.880
		Age <6m vs >12m	9	0	–	6	2.28 (1.53 - 3.41)	0.136
		Age <6m vs 7-59m	4	3	2.88 (0.94 - 8.80)	2	2.64 (1.50- 4.66)	0.211
		Age <2m vs >12m	7	2	3.60 (2.14 - 6.11)	6	5.25 (1.50 - 18.37)	0.231
	Female sex	Female vs male	23	5	1.20 (1.03 - 1.40)	16	1.13 (0.96- 1.34)	0.501
	Prematurity	Yes vs no	6	2	2.52 (1.68- 3.78)	2	0.61 (0.13 - 2.8)	0.020
	Ranking of birth	Higher vs lower	6	3	1.26 (0.86 - 1.85)	3	0.90 (0.53 – 1.51)	0.082
	Birth weight <2500g	<2500g vs >2500	9	4	2.94 (2.15- 4.03)	5	2.52 (1.56 - 4.08)	0.309
	Malnutrition	Severe malnutrition vs non-malnourished	21	7	5.24 (3.84 - 7.17)	13	4.12 (3.19 - 5.31)	0.026
		Moderate malnutrition vs non-malnourished	18	5	2.34 (1.81-3.05)	12	2.27 (1.62 - 3.19)	0.144
	Breastfeeding	Inadequate breastfeeding vs adequate	12	3	1.77 (1.16 - 2.70)	5	1.94 (1.43 - 2.63)	0.101
	HIV/AIDS	HIV	14	3	4.61 (3.65 - 5.83)	11	4.90 (3.84 – 6.25)	0.881
	Chronic disease	Yes vs no	12	3	5.20 (3.33- 8.13)	7	3.50 (2.45- 5.02)	0.041
	Malaria	Yes vs no	3	1	1.26 (0.86 - 1.85)	3	1.46 (1.02-2.11)	0.053
	Diarrhoea/dehydration	Yes vs no	6	4	2.66 (1.56 - 4.54)	5	2.67 (1.58 -4.54)	0.208
	Previous ALRI	Yes vs no	5	2	2.98 (1.77- 5.01)	2	1.73 (1.15 - 2.60)	0.447
	Severity of pneumonia as defined by WHO	Severe pneumonia vs pneumonia	6	1	3.22 (2.27-4.58)	4	2.92 (1.96- 4.35)	0.028
		Very severe pneumonia vs severe pneumonia or pneumonia	11	3	11.04 (6.74-18.08)	8	7.87 (5.68- 10.95)	0.448
	Respiratory Syncytial Virus	Yes vs no	6	1	0.51(0.29- 0.88)	5	0.31 8 (0.15- 0.63)	0.065
	Pneumocystis Carinii (jirovecii)	Yes vs no	5	1	4.95 (2.64-9.30)	5	4.79 (2.67-8.61)	0.283
Mother factors	Mother's age	Younger vs older	4	2	1.78 (0.99- 3.20)	2	2.07 (1.26- 3.42)	0.136
	Mother's educational level	Lower vs higher	14	4	1.38 (1.09 - 1.75)	5	1.53 (1.13-2.07)	0.977

	Mother's paid job	Yes vs no	3	2	1.13 (0.48- 2.65)	2	0.89 (0.52 -1.54)	0.815
**Socio-economic and environmental factors**	Socio-economic status	Lower income or father's social class vs higher	9	2	1.45 (1.17- 1.79)	4	1.95 (1.13 - 3.34)	0.530
	Water, sanitation and hygiene	Lack of sewerage/latrine vs presence	3	1	1.46 (1.01- 2.11)	2	1.67 (1.09-2.54)	0.850
		Lower quality of drinking water	3	1	2.10 (1.40 - 3.14)	2	1.96(1.28 - 3.01)	0.909
	Crowding	More people vs less	9	3	1.36 (0.88- 2.11)	3	1.14 (0.63- 2.05)	0.452
	Second-hand smoke exposure	Yes vs no	8	3	1.52 (1.13-2.03)	2	1.34 (0.85 - 2.13)	0.459
	Indoor pollution from solid fuels	Yes vs no	6	2	3.28 (2.33-4.62)	2	2.11 (1.33 - 3.33)	0.933
	Setting of residence	Rural vs urban	3	0	–	2	2.61 (1.02 - 6.68)	0.577
	Seasonality	Wet vs dry season	4	1	1.04 (0.63 -1.70)	3	0.82 (0.59-1.12)	0.702
**Health care factors**	Routine immunization	Yes vs no	12	4	0.53 (0.40- 0.70)	6	0.64 (0.44- 0.92)	0.241
	Good ante-natal practices	Antenatal care and birth-spacing vs absence	4	2	0.55 (0.35- 0.87)	1 (2 com-parisons)	0.45 (0.24- 0.82)	0.338
	Pre-hospital care	Pre-hospital antibiotics or community health worker consultation vs no	3	0	–	2	2.78 (1.49- 5.22)	0.447
