# Supplementary material for: Risk Factors for Mortality from Acute Lower Respiratory Infections (ALRI) in Children under Five Years of Age in Low and Middle-Income Countries: A Systematic Review and Meta-Analysis of Observational Studies
Source: PLoS One. 2015 Jan 30;10(1):e0116380. doi: 10.1371/journal.pone.0116380 (PMC4312071; doi:10.1371/journal.pone.0116380)
Supplement: S1 References — (PDF) [file pone.0116380.s008.pdf]

S1 References: References of the included studies

1 Agrawal PB, Shendumikar N, Shastri NJ (1995) Host factors and pmeumonia in hospitalized children. J Indian Med Assoc 93: 271-2.

2 Arifeen S, Black RE, Antelman G, Baqui A, Caulfield L, et al. (2001) Exclusive breastfeeding reduces acute respiratory infection and diarrhea deaths among infants in Dhaka slums. Pediatrics 108: e67.

3 Ayieko P, Okiro EA, Edwards T, Nyamai R, English M (2012) Variations in Mortality in Children Admitted with Pneumonia to Kenyan Hospitals. PLoS One 7: e47622.

4 Bachmann M, London L, Barron P (1996) Infant mortality rate inequalities in the Western Cape Province of South Africa. Int J Epidemiol 25: 966-72.

5 Bahl R, Frost C, Kirkwood BR, Edmond K, Martines J, et al. (2005) Infant feeding patterns and risks of death and hospitalization in the first half of infancy: multicentre cohort study. Bulletin of the World Health Organization 83: 418-26.

6 Bahwere P, De Mol P, Donnen P, Dramaix-Wilmet M, Butzler JP, et al. (2004) Improvements in nutritional management as a determinant of reduced mortality from community-acquired lower respiratory tract infection in hospitalized children from rural central Africa. Pediatr Infect Dis J 23: 739-47.

7 Banajeh SM, al-Sunbali NN, al-Sanahani SH (1997) Clinical characteristics and outcome of children aged under 5 years hospitalized with severe pneumonia in Yemen
63. Ann Trop Paediatr 17: 321-6.

8 Banajeh SM (1998) Outcome for children under 5 years hospitalized with severe acute lower respiratory tract infections in Yemen: a 5 year experience. J Trop Pediatr 44: 343-6.

9 Berkley JA, Munywoki P, Ngama M, Kazungu S, Abwao J, et al. (2010) Viral etiology of severe pneumonia among Kenyan infants and children. JAMA 303: 2051-7.

10 Chisti MJ, Duke T, Robertson CF, Ahmed T, Faruque AS, et al. (2011) Co-morbidity: exploring the clinical overlap between pneumonia and diarrhoea in a hospital in Dhaka, Bangladesh. Ann Trop Paediatr 31: 311-9.

11 Chisti MJ, Salam MA, Ashraf H, Faruque AS, Bardhan PK, et al. (2013) Clinical risk factors of death from pneumonia in children with severe acute malnutrition in an urban critical care ward of Bangladesh. Plos One 8: e73728.

12 Collings DA, Martin KS (1985) A retrospective analysis of childhood pneumonia in a district hospital. The Central African journal of medicine 31: 152-56.

13 Cotes K, Moreno-Montoya J, Porras-Ramírez A, Rico-Mendoza A, de la Hoz-Restrepo F (2012) Clinical characteristics of patients hospitalized with severe respiratory illness during influenza seasons in the cities of Bogota and Manizales, Colombia 2000-2006. Rev Salud Publica (Bogota) 14: 129-42.

14 De Francisco A., Morris J, Hall AJ, Armstrong Schellenberg JR, Greenwood BM (1993) Risk factors for mortality from acute lower respiratory tract infections in young Gambian children. Int J Epidemiol 22: 1174-82.

15 Deivanayagam N, Nedunchelian K, Ramasamy S, Sudhandirakannan, Ratnam SR (1992) Risk factors for fatal pneumonia: a case control study. Indian Pediatr 29: 1529-32.

16 Delport SD, Brisley T (2002) Aetiology and outcome of severe community-acquired pneumonia in children admitted to a paediatric intensive care unit. S Afr Med J 92: 907-11.

17 Demers AM, Morency P, Mberyo-Yaah F, Jaffar S, Blais C, et al. (2000) Risk factors for mortality among children hospitalized because of acute respiratory infections in Bangui, Central African Republic. Pediatr Infect Dis J 19: 424-32.

18 Djelantik IG, Gessner BD, Sutanto A, Steinhoff M, Linehan M, et al. (2003) Case fatality proportions and predictive factors for mortality among children hospitalized with severe pneumonia in a rural developing country setting. J Trop Pediatr 49: 327-32.

19 Duke T, Mgone J, Frank D (2001) Hypoxaemia in children with severe pneumonia in Papua New Guinea. Int J Tuberc Lung Dis 5: 511-9.

20 El Kholy AA, Mostafa NA, El-Sherbini SA, Ali AA, Ismail RI, et al. (2013) Morbidity and Outcome of Severe Respiratory Syncytial Virus Infection. Pediatr Int 55: 283-8.

21 Espinal MA, Báez J (1996) Mortalidad infantil intradomiciliaria por IRA en una región de salud de la República Dominicana. CENISMI Publicaciones técnicas V. Santo Domingo 1996.

22 Fagbule D, Adedoyin MA (2001) Clinical predictors of outcome in childhood pneumonia. Nigerian Journal of Paediatrics 17: 37-41.

23 Ferreira S, Sant'anna CC, March MD, Santos MA, Cunha AJ (2013) Lethality by pneumonia and factors associated to death. Jornal de Pediatria 90: 92-7.

24 Ghani AS, Morrow BM, Hardie DR, Argent AC (2002) An investigation into the prevalence and outcome of patients admitted to a pediatric intensive care unit with viral respiratory tract infections in Cape Town, South Africa. Pediatr Crit Care Med 13: e275-e281.

25 Graham SM, Mtitimila EI, Kamanga HS, Walsh AL, Hart CA, et al. (2000) Clinical presentation and outcome of Pneumocystis carinii pneumonia in Malawian children. Lancet 355: 369-73.

26 Graham SM, Mankhambo L, Phiri A, Kaunda S, Chikaonda T, et al. (2011) Impact of human immunodeficiency virus infection on the etiology and outcome of severe pneumonia in Malawian children. Pediatr Infect Dis J 30: 33-8.

27 Ramakrishna B, Graham SM, Phiri A, Mankhambo L, Duke T (2012) Lactate as a predictor of mortality in Malawian children with WHO-defined pneumonia. Arch Dis Child 97: 336-42.

28 Hildenwall H, Nantanda R, Tumwine JK, Petzold M, Pariyo G, et al. (2009) Care-seeking in the development of severe community acquired pneumonia in Ugandan children. Annals of Tropical Paediatrics 29: 281-9.

29 Hoque BA, Chakraborty J, Chowdhury JT, Chowdhury UK, Ali M, et al. (1999) Effects of environmental factors on child survival in Bangladesh: a case control study. Public Health 113: 57-64.

30 Hussain A, Ali SM, Kvåle G (1999) Determinants of mortality among children in the urban slums of Dhaka city, Bangladesh. Trop Med Int Health 4: 758-64.

31 Johnson AW, Aderele WI (1992) The association of household pollutants and socio-economic risk factors with the short-term outcome of acute lower respiratory infections in hospitalized pre-school Nigerian children. Ann Trop Paediatr 12: 421-32.

32 Johnson WBR, Aderele WI, Gbadero DA (1992) Host Factors and Acute Lower Respiratory-Infections in Preschool-Children. Journal of Tropical Pediatrics 38: 132-6.

33 Johnson AW, Osinusi K, Aderele WI, Gbadero DA, Olaleye OD, et al. (2008) Etiologic agents and outcome determinants of community-acquired pneumonia in urban children: a hospital-based study. J Natl Med Assoc 100: 370-85.

34 Kitchin OP, Wessels F, Masekela R, Becker P, Green RJ (2011) Costs of admission for paediatric pneumonia in a setting of human immunodeficiency virus infection. International Journal of Tuberculosis and Lung Disease 15: 1702-6.

35 Lehmann D, Heywood P (1996) Effect of birthweight on pneumonia-specific and total mortality among infants in the highlands of Papua New Guinea. P N G Med J 39: 274-83.

36 Lupisan SP, Ruutu P, Erma Abucejo-Ladesma P, Quiambao BP, Gozum L, et al. (2007) Predictors of death from severe pneumonia among children 2-59 months old hospitalized in Bohol, Philippines: implications for referral criteria at a first-level health facility. Trop Med Int Health 12: 962-71.

37 Madhi SA, Petersen K, Madhi A, Khoosal M, Klugman KP (2000) Increased disease burden and antibiotic resistance of bacteria causing severe community-acquired lower respiratory tract infections in human immunodeficiency virus type 1-infected children. Clin Infect Dis 31: 170-6.

38 Man WDC, Weber M, Palmer A, Schneider G, Wadda R, et al. (1988) Nutritional status of children admitted to hospital with different diseases and its relationship to outcome in The Gambia, West Africa. Tropical Medicine and International Health 3: 678-86.

39 Mathur NB, Garg K, Kumar S (2002) Respiratory distress in neonates with special reference to pneumonia. Indian Pediatr 39: 529-37.

40 McNally LM, Jeena PM, Gajee K, Thula SA, Sturm AW, et al. (2007) Effect of age, polymicrobial disease, and maternal HIV status on treatment response and cause of severe pneumonia in South African children: a prospective descriptive study. Lancet 369: 1440-51.

41 Millán T, Serani F, Vargas NA, Solange VM (1999) Biological ad social characteristics of infants who died from pneumonia in the Metropolitan Region of Chile, 1995. Rev Panam Salud Pública 6: 333-41.

42 Morrow BM, Hsaio NY, Zampoli M, Whitelaw A, Zar HJ (2010) Pneumocystis pneumonia in South African children with and without human immunodeficiency virus infection in the era of highly active antiretroviral therapy. Pediatr Infect Dis J 29: 535-9.

43 Mtango FD, Neuvians D, Broome CV, Hightower AW, Pio A (1992) Risk factors for deaths in children under 5 years old in Bagamoyo district, Tanzania. Trop Med Parasitol 43: 229-33.

44 Murtagh P, Giubergia V, Viale D, Bauer G, Pena HG (2009) Lower respiratory infections by adenovirus in children. Clinical features and risk factors for bronchiolitis obliterans and mortality. Pediatr Pulmonol 44: 450-6.

45 Naheed A, Saha SK, Breiman RF, Khatun F, Brooks WA, et al. (2009) Multihospital surveillance of pneumonia burden among children aged <5 years hospitalized for pneumonia in Bangladesh. Clin Infect Dis 48 Suppl 2: S82-9.

46 Nantanda R, Hildenwall H, Peterson S, Kaddu-Mulindwa D, Kalyesubula I, et al. (2008) Bacterial aetiology and outcome in children with severe pneumonia in Uganda. Ann Trop Paediatr 28: 253-60.

47 Nascimento-Carvalho CM, Rocha H, Santos-Jesus R, Benguigui Y (2002) Childhood pneumonia: clinical aspects associated with hospitalization or death. Braz J Infect Dis 6: 22-8.

48 Nathoo KJ, Nkrumah FK, Ndlovu D, Nhembe M, Pirie DJ, et al. (1993) Acute lower respiratory tract infection in hospitalized children in Zimbabwe. Ann Trop Paediatr 13: 253-61.

49 Niobey FM, Duchiade MP, Vasconcelos AG, de Carvalho ML, Leal MC, et al. (1992) Risk factors for death caused by pneumonia in children younger than 1 year old in a metropolitan region of southeastern Brazil. A case- control study. Rev Saude Publica 26: 229-38.

50 O'Callaghan-Gordo C, Bassat Q, Morais L, Diez-Padrisa N, Machevo S, et al. (2011) Etiology and epidemiology of viral pneumonia among hospitalized children in rural Mozambique: a malaria endemic area with high prevalence of human immunodeficiency virus. Pediatr Infect Dis J 30: 39-44.

51 Onyango FE, Steinhoff MC, Wafula EM, Wariua S, Musia J, et al. (1993) Hypoxaemia in young Kenyan children with acute lower respiratory infection. BMJ 306: 612-5.

52 Pérez P, Kogan A, Maggi C, Mendoza N (2007) Clinical evaluation and risk factors analysis in children with adenovirus respiratory infections. Rev Chil Pediatr 78: 261-7.

53 Post CL, Victora CG, Valente JG, Leal MC, Niobey FM, et al. (1992) Prognostic factors of hospital mortality from diarrhea or pneumonia in infants younger than 1 year old. A case-control study. Rev Saude Publica 26: 369-78.

54 Preidis GA, McCollum ED, Mwansambo C, Kazembe PN, Schutze GE, et al. (2011) Pneumonia and malnutrition are highly predictive of mortality among African children hospitalized with human immunodeficiency virus infection or exposure in the era of antiretroviral therapy. J Pediatr 159: 484-9.

55 Quiambao BP, Gatchalian SR, Halonen P, Lucero M, Sombrero L, et al. (1998) Coinfection is common in measles-associated pneumonia. Pediatr Infect Dis J 17: 89-93.

56 Quiambao BP, Ruutu PJ, Ladesma EA, Gozum LS, Inobaya MT, et al. (2009) Pneumonia among young infants in rural Southeast Asia (Bohol Island, Philippines). Trop Med Int Health 14: 1457-66.

57 Ramachandran P, Nedunchelian K, Vengatesan A, Suresh S (2012) Risk factors for mortality in community acquired pneumonia among children aged 1-59 months admitted in a referral hospital. Indian Pediatr 49: 889-95.

58 Rehfuess EA, Tzala L, Best N, Briggs DJ, Joffe M (2009) Solid fuel use and cooking practices as a major risk factor for ALRI mortality among African children. J Epidemiol Community Health 63: 887-92.

59 Reyes H, Perez-Cuevas R, Salmeron J, Tome P, Guiscafre H, et al. (1997) Infant mortality due to acute respiratory infections: the influence of primary care processes. Health Policy and Planning 12: 214-23.

60 Rodríguez CE, Hernández LJ, Aristizábal R, Guzmán MC, Castillo C, et al. (2010) Factors affecting mortality from acute respiratory infections in a population of children under five years living in the city of Bogota. Investig segur soc salud 12: 21-38.

61 Rodríguez DA, Rodríguez-Martínez CE, Cárdenas AC, Quilaguy IE, Mayorga LY, et al. (2014) Predictors of severity and mortality in children hospitalized with respiratory syncytial virus infection in a tropical region. Pediatr Pulmonol 49: 269-76.

62 Roth A, Gustafson P, Nhaga A, Djana Q, Poulsen A, et al. (2005) BCG vaccination scar associated with better childhood survival in Guinea-Bissau. Int J Epidemiol 34: 540-7.

63 Sehgal V, Sethi GR, Sachdev HP, Satyanarayana L (1997) Predictors of mortality in subjects hospitalized with acute lower respiratory tract infections. Indian Pediatr 34: 213-9.

64 Shah SI, Zemichael O, Meng HD (2012) Predictors of mortality, length of stay and co-morbid hypothermia in hospitalized neonates with pneumonia in Eritrea, Africa. J Trop Pediatr 58: 238-41.

65 Shann F, Barker J, Poore P (1989) Clinical signs that predict death in children with severe pneumonia. Pediatr Infect Dis J 8: 852-5.

66 Sigauque B, Roca A, Bassat Q, Morais L, Quinto L, et al. (2009) Severe pneumonia in Mozambican young children: clinical and radiological characteristics and risk factors. J Trop Pediatr 55: 379-87.

67 Smyth A, Carty H, Hart CA (1998) Clinical predictors of hypoxaemia in children with pneumonia. Ann Trop Paediatr 18: 31-40.

68 Smyth A, Tong CY, Carty H, Hart CA (1997) Impact of HIV on mortality from acute lower respiratory tract infection in rural Zambia. Arch Dis Child 77: 227-30.

69 Sutanto A, Gessner BD, Djlantik I, Steinhoff M, Murphy H, et al. (2002) Acute respiratory illness incidence and death among children under two years of age on Lombok Island, Indonesia. Am J Trop Med Hyg 66: 175-9.

70 Tupasi TE, Velmonte MA, Sanvictores ME, Abraham L, de Leon LE, et al. (1988) Determinants of morbidity and mortality due to acute respiratory infections: implications for intervention. J Infect Dis 157: 615-23.

71 Tupasi TE, Mangubat NV, Sunico ME, Magdangal DM, Navarro EE, et al. (1990) Malnutrition and acute respiratory tract infections in Filipino children. Rev Infect Dis 12 Suppl 8: S1047-54.

72 Tupasi TE, Lucero MG, Magdangal DM, Mangubat NV, Sunico ME, et al. (1990) Etiology of acute lower respiratory tract infection in children from Alabang, Metro Manila. Rev Infect Dis 12 Suppl 8: S929-39.

73 Uriyo J, Gosling RD, Maddox V, Sam NE, Schimana W, et al. (2006) Prevalences of Pneumocystis jiroveci, Mycobacterium tuberculosis and Streptococcus pneumoniae

infection in children with severe pneumonia, in a tertiary referral hospital in northern Tanzania. Annals of tropical medicine and parasitology 100: 245-9.

74 Veirum JE, Sodemann M, Biai S, Jakobsen M, Garly ML et al. (2005) Routine vaccinations associated with divergent effects on female and male mortality at the paediatric ward in Bissau, Guinea-Bissau. Vaccine 23: 1197-204.

75 Véjar L, Casterán JC, Navarrete P, Sánchez S, LeCerf P, et al. (2000) Risk factors for home deaths due to pneumonia among low socioeconomic level Chilean children, Santiago de Chile (1994). Rev Med Chil 128: 627-32.

76 Victora CG, Barros FC, Vaughan JP, Teixeira AM (1987) Birthweight and infant mortality: a longitudinal study of 5914 Brazilian children. Int J Epidemiol 16: 239-45.

77 Victora CG, Smith PG, Barros FC, Vaughan JP, Fuchs SC (1989) Risk factors for deaths due to respiratory infections among Brazilian infants. Int J Epidemiol 18: 918-25.

78 Victora CG, Smith PG, Vaughan JP, Nobre LC, Lombardi C, et al. (1988) Influence of birth weight on mortality from infectious diseases: a case-control study. Pediatrics 81: 807-11.

79 Weissenbacher M, Carballal G, Avila M, Salomon H, Harisiadi J, et al. (1990) Etiologic and clinical evaluation of acute lower respiratory tract infections in young Argentinian children: an overview. Rev Infect Dis 12 Suppl 8: S889-98.

80 Ye Y, Zulu E, Mutisya M, Orindi B, Emina J, et al. (2009) Seasonal pattern of pneumonia mortality among under-five children in Nairobi's informal settlements. Am J Trop Med Hyg 81: 770-5.

81 Yoon PW, Black RE, Moulton LH, Becker S (1997) The effect of malnutrition on the risk of diarrheal and respiratory mortality in children < 2 y of age in Cebu, Philippines. Am J Clin Nutr 65: 1070-7.

82 Yoon PW, Black RE, Moulton LH, Becker S (1996) Effect of not breastfeeding on the risk of diarrheal and respiratory mortality in children under 2 years of age in Metro Cebu, The Philippines. Am J Epidemiol 143: 1142-8.

83 Zar HJ, Hanslo D, Tannenbaum E, Klein M, Argent A, et al. (2001) Aetiology and outcome of pneumonia in human immunodeficiency virus-infected children hospitalized in South Africa. Acta Paediatr 90: 119-25.
